# Nanopore sequencing identifies high-frequency somatic structural variations in laryngeal squamous cell carcinoma genomes

**DOI:** 10.1101/gr.281046.125

**Published:** 2026-11

**Authors:** Xuyan Liu, Lin Xia, Yixin Qiao, Yang Li, Yan Huang, Bingyan Yue, Xi Liang, Xin Yang, Honghui Zhang, Jiaxun Zhang, Xiao Chen, Dan Xie, Jifeng Liu

**Affiliations:** 1 Laboratory of Omics Technology and Bioinformatics, Frontiers Science Center for Disease-Related Molecular Network, State Key Laboratory of Biotherapy, West China Hospital, Sichuan University, Chengdu, Sichuan 610041, China;; 2 Department of Otolaryngology–Head and Neck Surgery, West China Hospital, Sichuan University, Chengdu, Sichuan 610041, China;; 3 Deep Underground Space Medical Center, West China Hospital, Sichuan University, Chengdu, Chengdu, Sichuan 610041, China

## Abstract

Laryngeal squamous cell carcinoma (LSCC) is an aggressive cancer associated with poor quality of life. Understanding the somatic mutations in its genome is crucial for elucidating its occurrence and progression. Although somatic structural variations (SVs) have been documented in LSCC, conventional short-read sequencing lacks the sensitivity to effectively detect high-frequency SVs, variants that play a crucial role in tumorigenesis, shared across multiple samples. Here, we present SomaGauss-SV, a somatic SV detection workflow leveraging nanopore long-read sequencing data. Benchmarking against five paired tumor cell line data sets shows SomaGauss-SV consistently achieves a balanced high precision and recall. SomaGauss-SV applied to 15 paired LSCC tumor-blood samples uncovers a comprehensive SV landscape and reveals a significant positive correlation between somatic deletion burden and smoking intensity. Furthermore, a high-frequency somatic simple repeat expansion is identified in 28/39 (71.79%) of LSCC patients, which upregulates the expression of genes *TP53BP2* and *FBXO28* through spatial proximity. These findings underscore the potential of long-read sequencing and SomaGauss-SV for uncovering recurrent somatic SVs in LSCC, providing valuable resources for biomarker discovery.

Laryngeal squamous cell carcinoma (LSCC) is the second most common subtype of head and neck squamous cell carcinoma (HNSCC), characterized by marked gender differences and regional variability in both incidence and mortality rates. The incidence rate in males is approximately eight times that of females (Han et al. 2024). LSCC pathogenesis is closely related to modifiable risk factors, such as tobacco exposure and alcohol consumption (Johnson et al. 2020; Sung et al. 2021; Bray et al. 2024; Chen et al. 2024). However, the molecular mechanisms by which these exogenous factors induce genomic instability remain unclear. Early symptoms of LSCC are often overlooked, resulting in >50% of patients being diagnosed at advanced stages, particularly those with supraglottic tumors (Steuer et al. 2017; Nocini et al. 2020). This delay in diagnosis typically requires aggressive treatments that severely impair patients’ respiratory and vocal functions, thereby significantly diminishing their quality of life (Obid et al. 2019). Despite advances in treatment regimens, combining surgery with radiotherapy and chemotherapy, the 5-year overall survival rate has shown limit improvement (Wang et al. 2020; Cavaliere et al. 2021), highlighting the urgent need for molecular biomarkers specific to LSCC. Compared with other HNSCC subtypes, LSCC has unique biological characteristics and epidemiological features (Cavaliere et al. 2021; Pan et al. 2025), making specialized research essential to address its unique diagnostic challenges and the critical need for organ function preservation.

In recent years, pancancer genomic studies have highlighted the pivotal role of structural variants (SVs) in the occurrence and progression of cancer (Cosenza et al. 2022). SVs, which encompass large genomic rearrangements exceeding 50 bp, include deletions (DELs), insertions (INSs), duplications (DUPs), inversions (INVs), and translocations (TRAs) (van Belzen et al. 2021). These variants drive oncogenesis through mechanisms such as gene disruption and fusion, copy number alterations, and three-dimensional genome reorganization (Beroukhim et al. 2017). Large-scale cancer cohort studies have shown that somatic SVs are the most prevalent class of driver mutations in cancer, vastly outnumbering single-nucleotide variants (SNVs) and small insertions/deletions (indels) (Cosenza et al. 2022). Given their abundance and diverse roles in cancer genomes, a comprehensive understanding of somatic SVs’ scope and mechanisms is essential for uncovering key cancer mutations in patient tumors.

However, despite the widespread clinical application of somatic SV detection methods based on second-generation short-read sequencing, these approaches have inherent limitations in sensitivity for smaller SVs (≤10 kb) and in resolving repetitive sequence variants (ICGC/TCGA Pan-Cancer Analysis of Whole Genomes Consortium 2020; Choo et al. 2023). The advent of nanopore long-read sequencing technology offers a promising solution to overcome this bottleneck. With read length >10 kb, nanopore sequencing can span SV breakpoint regions in their entirety, enabling comprehensive mapping of somatic SV landscapes. Recently, several somatic SV algorithms, such as nanomonsv (Shiraishi et al. 2023), Severus (Keskus et al. 2026), and SVision-pro (Sahlin et al. 2023), have been developed to harness this potential. However, existing tools still face challenge in correcting alignment biases in repetitive regions, distinguishing mixed signals of multiscale INSs and DELs, and their practical application in clinical samples of sufficient scale remains limited (Sahlin et al. 2023). Therefore, developing detection workflows suitable for different research scenarios is a key step for advancing the clinical interpretation of SVs.

Here, we present SomaGauss-SV, a nanopore-based somatic SV caller developed to address this limitation. We rigorously benchmark its performance in paired tumor–normal cell lines using complementary, orthogonal validation strategies to establish robustness before clinical deployment. We then apply this framework to delineate the somatic SV landscape of primary LSCC and interrogate potential biological and etiological associations. Ultimately, this study aims to deliver a high-performance workflow for somatic SV discovery, elucidate recurrent SV-linked mechanisms in LSCC, and generate a comprehensive, clinically annotated SV resource to enable future mechanistic and translational investigations.

## Results

### SomaGauss-SV demonstrates excellent performance in detecting somatic SVs

Current long-read sequencing algorithms for detecting SVs often exhibit reduced accuracy in repetitive genomic regions. This limitation arises from inherent biases in traditional seed-chain alignment methods (Supplemental Fig. S1; Sahlin et al. 2023). The lack of systematic alignment error correction in existing SV detection tools further constrains their utility for somatic SV calling. To address these challenges, we present SomaGauss-SV, a novel workflow (Fig. 1A) that integrates a mixed Gaussian distribution model with alignment error correction to improve the accuracy of somatic SV detection. Its core innovation lies in applying tailored Gaussian modeling for different SV types coupled with stringent population-based filtering to distinguish somatic events (for full details, see Methods).

**Figure 1.**
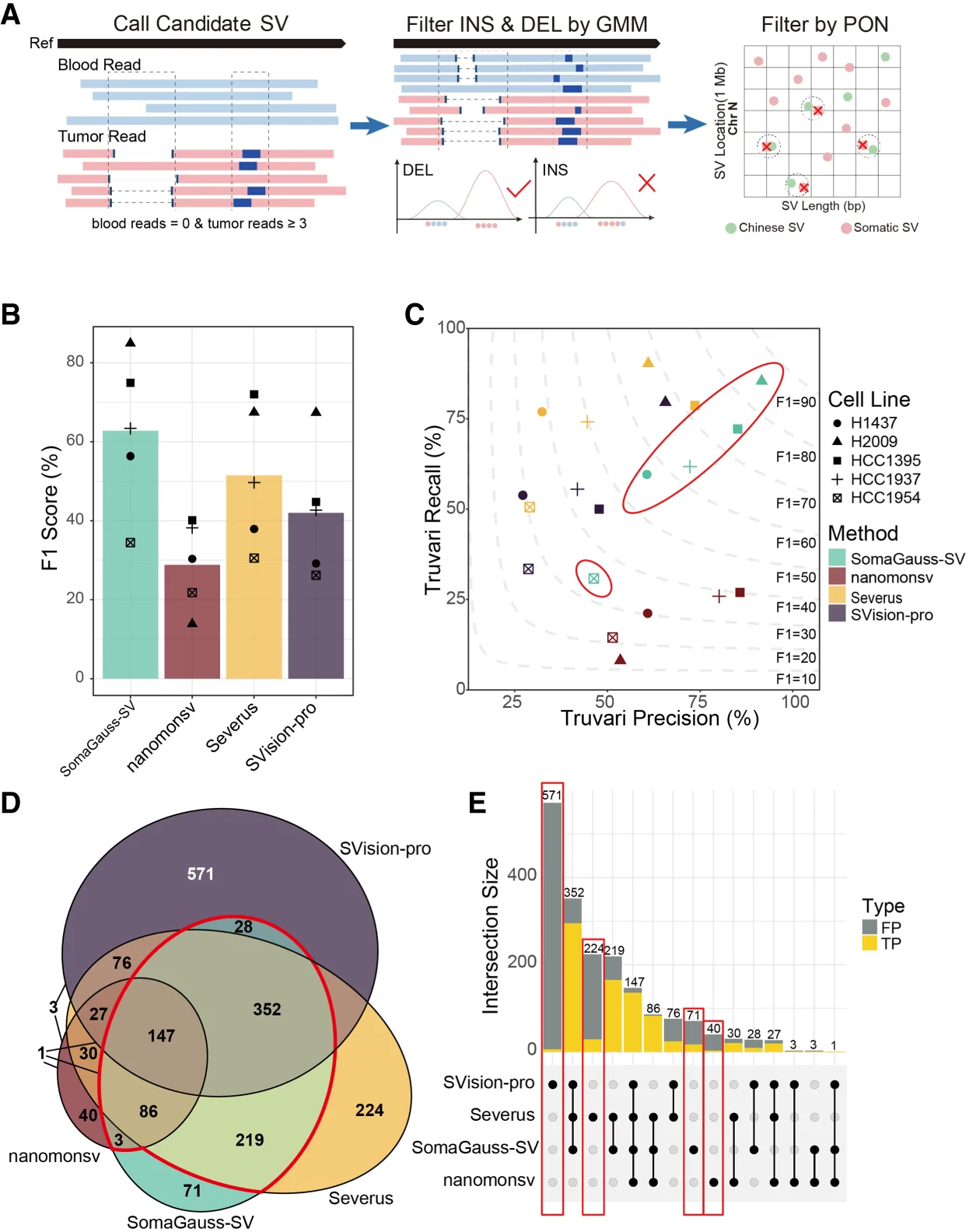
Overview and evaluation of somatic SV detection software. (*A*) Schematic of the SomaGauss-SV workflow, which consists of three main steps: (1) detection of candidate somatic SVs, screening of somatic SVs through a merge method; (2) filtering of insertions (INS) and deletions (DEL) using a Gaussian mixture model (GMM); and (3) filtering of SVs using data from the Chinese normal population. (*B*) Bar chart showing the F1-scores for different software. The height of the bars represents the average F1-score for each software, and different shapes of points indicate the F1-scores for different cell lines. (*C*) Contour plot of software evaluation. The *x*-axis represents precision, the *y*-axis represents recall, the contours in the plot represent F1-scores, and software and cell lines are distinguished by the color and shape, respectively, of the points. (*D*) Venn diagram of software detection results in the HCC1395 cell line. Numbers indicate the number of SVs in the intersections between software, and red lines indicate the intersections involving SomaGauss-SV with other software. (*E*) UpSet plot of true-positive (TP) proportions for different software in the HCC1395 cell line. Yellow indicates TP, gray indicates false-positive (FP), red boxes indicate the proportion of TP unique to each software, and black circles below indicate intersections between software.

To systematically assess the performance of SomaGauss-SV in somatic SVs detection, we utilized a public resource that provides five tumor–normal cell line pairs (HCC1395/HCC1395BL, HCC1937/HCC1937BL, HCC1954/HCC1954BL, H1437/H1437BL, H2009/H2009BL) (Supplemental Table S1) with matched sequencing data and a curated SV ensemble as the ground truth (Keskus et al. 2026). We employed two independent evaluation strategies, Truvari (English et al. 2022) and minda (Keskus et al. 2026), to compare SomaGauss-SV against established somatic SV callers: nanomonsv (Shiraishi et al. 2023), SVision-pro (Wang et al. 2025), and Severus (Keskus et al. 2026). For a fair comparison in this controlled setting, we omitted the panel-of-normal (PON) filtration step.

SomaGauss-SV consistently achieved the highest F1-scores across all five cell line pairs using the Truvari evaluation strategy, achieving a mean F1-score of 62.8% (±19.2% SD), significantly outperforming Severus (51.5% ± 18.0%), SVision-pro (42.0% ± 16.3%), and nanomonsv (28.9% ± 11.1%) (Fig. 1B). Notably, SomaGauss-SV achieved peak performance (84.9% F1-score) in the H2009 cell line, which is characterized by INS-dominant SV profiles (mean comparator performance: 57.3%) (Supplemental Fig. S2A), highlighting its particular strength in resolving this challenging variant class. In contrast, the HCC1395 cell line, with its high SV burden, showed narrower performance differentials between SomaGauss-SV (74.9%) and Severus (72.0%), both substantially outperforming the other tools (mean: 42.5%). The genome-doubled HCC1954 cell line exhibited universally lower F1-scores across all platforms (mean: 28.2% vs. 50.8% in nonpolyploid lines), suggesting that polyploidization introduces systematic challenges in SV detection. A critical analysis revealed the precision–recall trade-offs of each tool (Fig. 1C): nanomonsv exhibited higher precision (59.1% vs. 50.7% for competitors’ mean precision), whereas Severus and SVision-pro prioritized recall (64.3% vs. 40.6% for competitors’ mean recall). SomaGauss-SV uniquely balanced both metrics (mean precision: 64.0%; mean recall: 61.9%). Considering practical application, we also evaluated the computational space and time complexity. SomaGauss-SV exhibited the smallest maximum resident set size, and its elapsed real time was significantly less than that of Severus, despite their similar F1-scores (Supplemental Fig. S2B).

The intercell line performance variation strongly correlated with the composition of somatic SV types. To explore this relationship further, we conducted stratified analyses by SV types using Truvari with type-specific ensemble settings (Supplemental Fig. S2C). SomaGauss-SV consistently outperformed other tools across all SV types. Notably, in INS detection, SomaGauss-SV achieved an F1-score of 59.3%, outperforming comparator tools by 13.9%–93.1% (mean F1-score of comparators: 25.5%). For DUP, SomaGauss-SV exhibited performance comparable to that of Severus (67.7% vs. 69.1%). Although all tools showed relatively higher efficacy in DEL detection (mean F1-scores: 45.4%–70.2%), both breakend (BND) and INV detection proved challenging across platforms (mean F1 < 5.7%). This limitation was most pronounced in the BND-rich HCC1954 data set, in which BNDs constituted 64.2% of SVs (Supplemental Fig. S2C). To ensure a robust and unbiased evaluation, we conducted a complementary assessment using minda (for full details, see Supplemental Table S2), which matches variants based on breakpoint coordinates and length, independent of SV type annotation. Re-evaluation with minda yielded two key insights. First, under this more lenient and type-agnostic matching scheme, F1-scores for all tools increased, with the most substantial improvements (15.56%–48.47%) observed in the BND-rich HCC1954 data set (Supplemental Fig. S2D), highlighting the inherent difficulty in computationally distinguishing BND. Second, and more importantly, SomaGauss-SV's advantage in precision became even more pronounced. It achieved the highest average precision (79.20%) across four cell lines (Supplemental Fig. S2E), demonstrating its ability to generate a highly reliable, low false-positive call set, a critical feature for downstream biological discovery. In light of the inherent difficulty in computationally detecting complex SVs like INV and BND, our final analysis pipeline incorporates a mandatory manual curation step for these variant types in tumor–normal pairs to ensure accuracy (see Methods). Beyond type-specific performance, we analyzed the concordance between different SV detection tools using the HCC1395/HCC1395BL cell line pair (Fig. 1D). SomaGauss-SV demonstrated superior consensus validity (92.2% overlap) and the highest true-positive rate (23.94%) among its unique calls (Fig. 1E). Although consensus calling improved true-positive rates, it incurred substantial variant loss (52.3%) and systematic class biases (e.g., the proportion of DEL detection increased by 29.0%) (Supplemental Fig. S2F), underscoring its limitations for comprehensive SV profiling. Collectively, these findings position SomaGauss-SV as the optimal solution for our LSCC study, balancing precision and recall while preserving both high-confidence consensus variants and biologically relevant unique calls, critical advantages for downstream clinical interpretation.

### Pathological information and sequencing quality data display of ONT whole-genome sequencing samples

To interrogate the Somatic SVs landscape of LSCC, we included tumor samples, comprised with matched blood samples from 15 treatment-naive individuals with primary, HPV-negative LSCC (Supplemental Table S3; Fig. 2A). These tumor samples represented a broad spectrum of clinical stages and anatomical subtypes (Fig. 2B,C). All patients were male, with a mean age of 63 years (median: 60). Smoking exposure was assessed using the smoking index, calculated as the number of cigarettes per day multiplied by years of smoking (Zhou et al. 2023). Fourteen of the fifteen patients had a history of smoking, with an average smoking index of 648 (median: 610) (Supplemental Table S3; Fig. 2D). These cohort characteristics align with previous studies, demonstrating that LSCC predominantly affects middle-aged and elderly males and is strongly associated with smoking (Johnson et al. 2020; Lechien et al. 2022), reflecting the typical demographic profile of LSCC patients.

**Figure 2.**
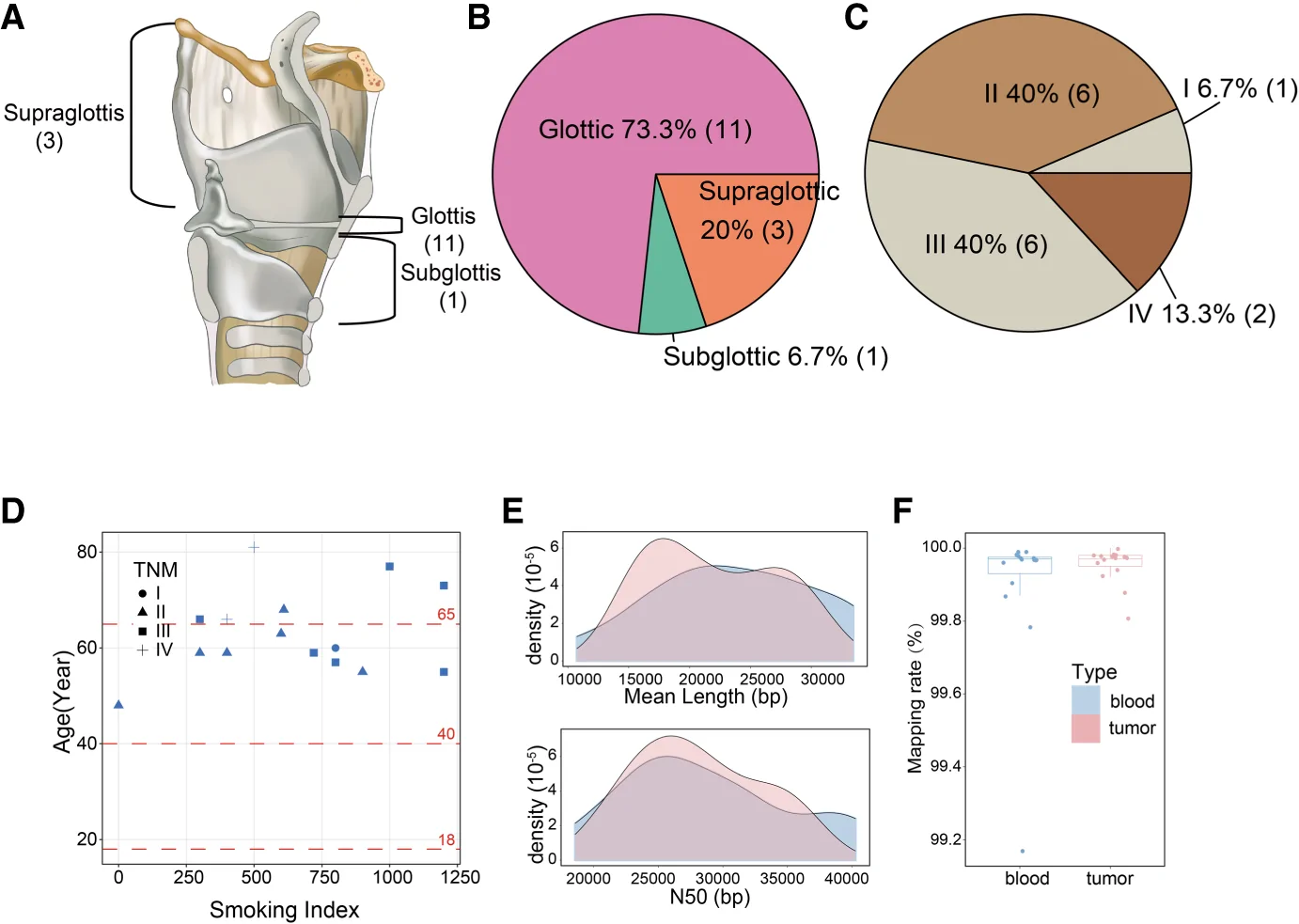
Overview of pathological information and sequencing data. (*A*) Display of sampling sites for LSCC. There are three cases of supraglottic type, 11 cases of glottic type, and one case of subglottic type. (*B*) Pie chart of the proportion of sampling sites for LSCC. (*C*) Pie chart of the proportion of TNM staging for LSCC patients. (*D*) Pathological scatter plot for LSCC patients. The *x*-axis represents the smoking index of patients (calculated as the number of cigarettes per day × smoking years), the *y*-axis represents patient age, and the shape of the points indicates TNM staging. (*E*) Read-length distribution plot of nanopore sequencing. The *upper* part shows the distribution of average read lengths (in base pairs) for tumor (pink) and blood (blue), and the *lower* part shows the N50 distribution for tumor and blood. (*F*) Box plot of read mapping rates. Blue represents blood, and pink represents tumor.

Whole-genome sequencing (WGS) of paired tumor and blood samples was performed using the PromethION platform (R9: nine samples, R10: six samples) (Supplemental Table S3), generating 2.10 Tb of data. After quality control (sequencing quality > 7, read length > 1 kb), tumor samples yielded an average N50 of 28 kb (range: 20 kb to 36 kb) and an average of 94 billion bases per sample, equating to a mean sequencing depth of 30× (range: 28× to 33×) (Fig. 2E). Matched blood samples generated an average N50 of 28 kb (range: 19 kb to 40 kb) and an average of 49 billion bases per sample, resulting in a mean sequencing depth of 15× (range: 12× to 18×) (Fig. 2E). Alignment of the clean long-read sequencing data to the GRCh38 (hg38) reference genome using minimap2 yielded an average alignment rate of 99.95% for tumor samples and 99.90% for blood samples (Supplemental Table S4; Fig. 2F).

### Discovery of the positive relation between somatic DEL burden and tobacco exposure

Using SomaGauss-SV, 8128 somatic SVs were identified across the 15 paired samples, with an average of 541 somatic SVs per tumor samples. Somatic INSs were the most common variant, comprising 54.7% of the total, followed by DELs (19.7%), breakends (BND; 18.0%), INVs (4.0%), and DUPs (3.6%) (Supplemental Fig. S3A). The distribution of SV types in individual samples was consistent with this overall pattern (Fig. 3A). Notably, samples with higher smoking indices displayed a significantly greater somatic SV burden (Pearson correlation coefficient, *P* < 0.05, *r* = 0.54) (Supplemental Fig. S3B). DEL contributed most prominently to this trend, exhibiting the strongest correlation with the smoking index among all SV types (Pearson correlation coefficient, *P* < 0.05, *r* = 0.67) (Fig. 3B). To dissect this relationship more rigorously, we performed negative binomial regression, treating the burden of specific SV types as the dependent variable while controlling for TNM stage, age, and sequencing depth. This multivariate analysis identified the smoking index as a highly significant, independent positive predictor for DEL burden specifically (*P* = 0.000596) (Supplemental Fig. S3C). To strengthen the generalizability of this finding, we validated the association in an independent HNSCC cohort from the ICGC (*n* = 44, 19 of whom had no smoking history). After controlling for TNM stage, age, and sex via negative binomial regression, the smoking index remained significantly associated with DEL burden (*P* = 0.0498) (Supplemental Fig. S3D).This finding similar with previous studies indicating that smoking increases the somatic mutation burden in bronchial epithelial cells and LSCC (Degawa et al. 1994; Yoshida et al. 2020) suggested that tobacco exposure may promote somatic DEL by introducing DNA double-strand breaks (Degawa et al. 1994; Yoshida et al. 2020; Caliri et al. 2021).

**Figure 3.**
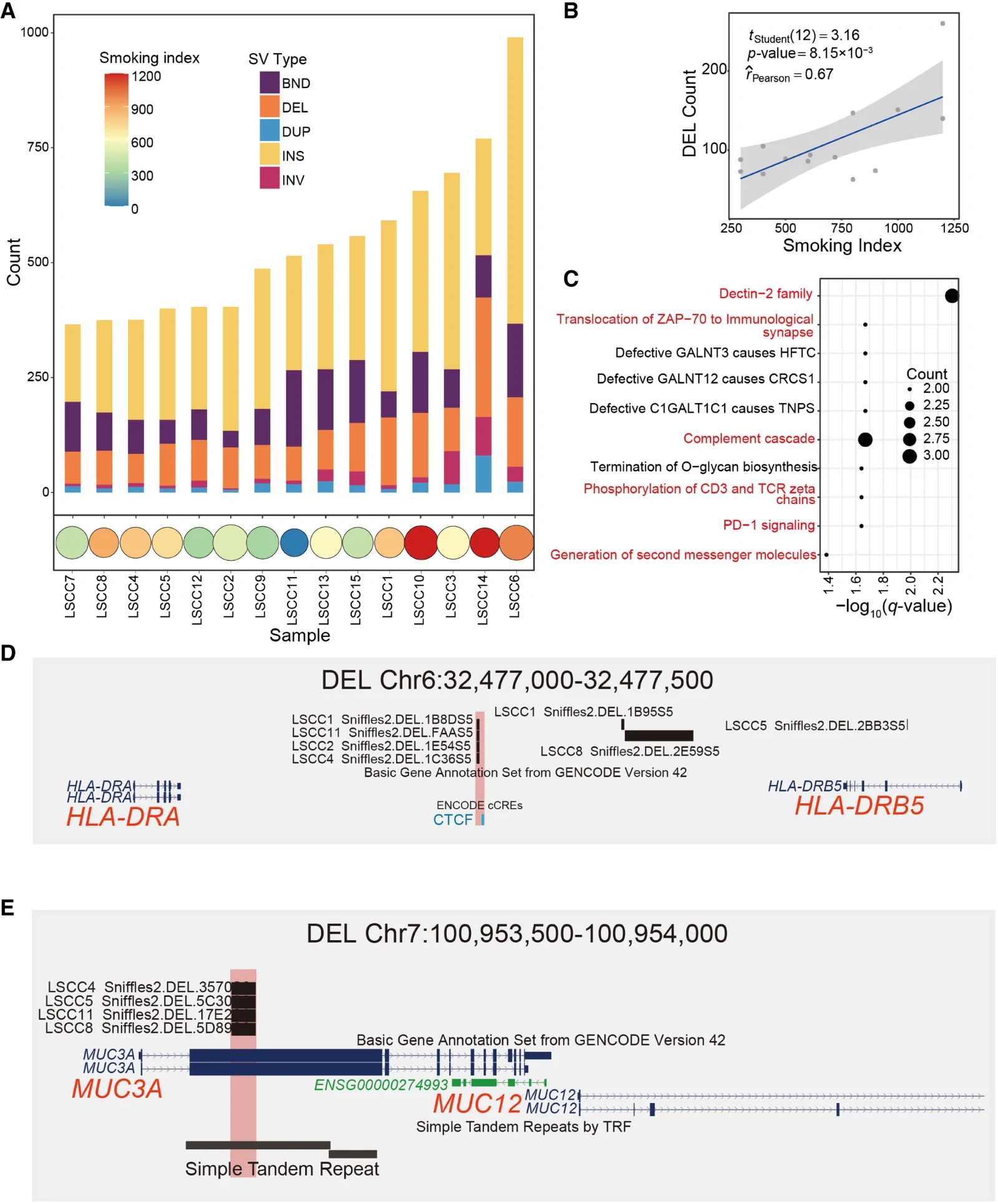
Relationship between somatic DEL and tobacco exposure. (*A*) Stacked bar chart of somatic structural variation counts and smoking index distribution. The *upper* stacked bar chart shows the number of different SVs for each sample, and the *lower* bubble chart shows the smoking index for corresponding samples, in which higher indices are represented by redder colors. (*B*) Correlation plot of somatic DEL counts and smoking index. The *x*-axis represents the smoking index, and the *y*-axis represents the number of DEL, with degrees of freedom, *P*-value, and Pearson correlation coefficient marked on the plot. (*C*) Reactome pathway enrichment plot for genes affected by common DEL in multiple samples from smokers (*Q*-value < 0.05). The *x*-axis represents −log_10_(*Q*-value), and the *y*-axis represents the names of enriched pathways, with immune-related pathways highlighted in red. The size of the circles indicates the number of enriched genes. (*D*) Browser plot of somatic DELs (Chr 6: 32,477,000–32,477,500) illustrating the following epigenomic signals: sample DEL location, ENCODE-annotated gene transcript, and candidate *cis*-regulatory elements (cCREs) predicted by ENCODE. (*E*) Browser plot of somatic DELs (Chr 7: 100,953,500–100,954,000) illustrating the following epigenomic signals: sample DEL location, ENCODE-annotated gene transcript, and simple tandem repeats identified by TRF.

To investigate the functional impact of somatic DELs in smoking-related LSCC, we focused on the 42 somatic DEL regions (after merging adjacent windows, as described in Methods, Sample Enrichment Analysis) that were shared by three or more samples from smokers. Using GREAT (McLean et al. 2010), these loci were mapped to 49 putative distal regulatory target genes. Pathway enrichment analysis revealed that six of the top 10 enriched pathways (*q* < 0.05) were immune related, including the Dectin-2 family (Graham and Brown 2009), translocation of ZAP70 to immunological synapse (Blanchard et al. 2002), and complement cascade (Fig. 3C; Afshar-Kharghan 2017). GO analysis further supported this immune-focused pattern, with nine of the top 10 biological processes (*q* < 0.05) related to lymphocyte activation and cytokine responses (Supplemental Fig. S3E). These findings supported the role of smoking in tumorigenesis through immune-inflammatory responses (Caliri et al. 2021), suggesting that somatic DEL frequently carried by high-smoking LSCC patients may regulate the smoking-associated immune microenvironment.

Among the immune-related somatic DEL regions, a particular somatic DEL, shared by four smoking-related LSCC samples, may affect the expression of *HLA-DRA* and *HLA-DRB5*, which are key immune modulators involved in tumor immune evasion. This high-frequency DEL is located near a CTCF binding site, identified in the MCF-7 and A673 cell lines, 265 bp apart (Fig. 3D). The CTCF site is predicted to regulate *HLA-DRA* and *HLA-DRB5* (The ENCODE Project Consortium 2012). We propose that the somatic DEL disrupts CTCF binding, leading to altered chromatin architecture and dysregulated expression of *HLA-DRA* and *HLA-DRB5*. Additionally, we identified a somatic DEL in the *MUC3A* gene that encodes mucin and has been associated with smoking-induced changes in chronic obstructive pulmonary disease (COPD) (Merikallio et al. 2023). *MUC3A* expression is reported to be higher in smokers than in nonsmokers owing to tobacco exposure (Gendler and Spicer 1995). The somatic DEL, located at exon 2 of *MUC3A* gene (DEL length: 1120 bp to 1123 bp), contains a simple tandem repeat sequence (motif: ACC) (Fig. 3E). PCR analysis of cDNA from clinical samples demonstrated that this genomic deletion results in a deletion within the *MUC3A* transcript, resulting in a shorter band (∼346–349 bp) compared with the expected full-length product (1469 bp) (Supplemental Fig. S3F). This exon encodes a peptide rich in threonine (Thr) and serine (Ser), which are essential for glycosylation and for mucin 3A's role in protecting the epithelial barrier from environmental damage (Kufe 2009; Bhatia et al. 2019). Therefore, the somatic DEL is likely to cause truncation of this critical peptide domain, potentially impairing mucin 3A function.

### Characteristics of somatic SVs

We characterized somatic SVs in LSCC by analyzing SV size, sequence context, and breakpoint distribution. Our results highlight distinct length preferences across SV types (Fig. 4A). Somatic DEL and INS were characterized by shorter lengths and narrower distributions. DEL (mean: 1493 bp, range: 50 bp to 30 kb) had a peak at size of 70 bp, whereas INS (mean 1107 bp, range: 50 bp to 24 kb) exhibited a trimodal distribution with peaks at 70 bp, 170 bp, and 300 bp. In contrast, DUP and INV displayed larger, more dispersed length distributions (DUP mean: 39 kb; INV mean: 5.3 Mb). These patterns are similar with prior study on somatic SVs in HCC (Zeng et al. 2025).

**Figure 4.**
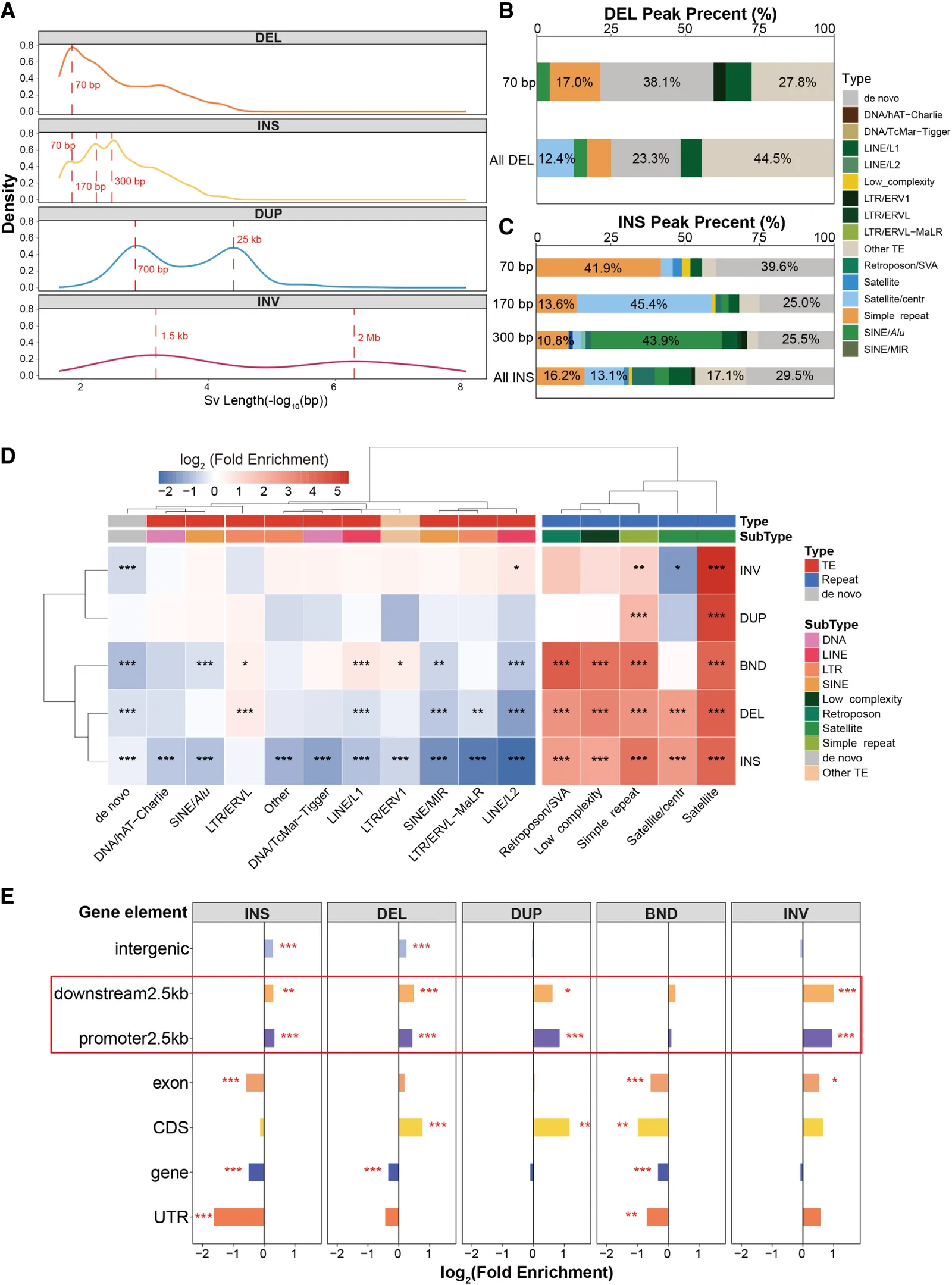
Overview of SV length and site enrichment. (*A*) Length distribution plot for different SV types. From *top* to *bottom* are DEL, INS, DUP, and INV, with red dashed lines marking the peaks for each SV type. (*B*) Stacked bar chart of sequence annotation proportions at DEL peaks. (*C*) Stacked bar chart of sequence annotation proportions at INS peaks. (*D*) Heatmap of somatic SVs site enrichment in repeat regions. Each row represents a different SV type, and each column represents a different repeat region type, with color intensity indicating log_2_(fold enrichment). Repeat regions are categorized into transposable elements region (TE), repeat region (Repeat), and nonrepetitive regions (de novo). (*E*) Bar chart of somatic SVs site enrichment for gene elements. The *x*-axis represents log_2_(fold enrichment), the *y*-axis represents gene element names, and facets represent different SV types. Fisher's exact test, (*) *P*-value < 0.05, (**) *P*-value < 0.01, (***) *P*-value < 0.001.

To investigate the sequence determinants of somatic SVs, we analyzed the sequence feature of SVs at characteristic size peaks. Given the relatively uniform length distributions of DUP and INV (Fig. 4A; Supplemental Fig. S4A), we focused on DEL and INS (Fig. 4B,C). At the 70 bp peak, both somatic DEL and INS were significantly enriched in simple repeat sequences. Notably, AAAG motifs predominated in somatic DEL (30%) (Supplemental Fig. S4B), whereas somatic INS exhibited a strong AT-richness (58.6% AT) (Supplemental Fig. S4C). The 170 bp INS peak showed a notable association with centromeric regions. Specifically, 45.4% of these 170 bp INS harbored satellite/CEN (monomers: ∼170 bp [Aldrup-MacDonald et al. 2016], Pearson's chi-squared test, *P*-value = 1.1 × 10^−6^). Among these satellite/CEN-containing INS, 55.6% were directly located within centromeres, a significant enrichment compared with the genome-wide background of 12.3% (Pearson's chi-squared test, *P*-value < 0.001) (Supplemental Fig. S4D). Even those not residing inside centromeres clustered in close proximity, with 62.7% found within 1 Mb of centromeric regions (Wilcoxon test, *P*-value < 0.001) (Supplemental Fig. S4D). This pericentromeric enrichment pattern was further confirmed by realignment of Chromosome 20 reads to the complete T2T-CHM13 assembly (Wilcoxon test, *P*-value < 0.001) (Supplemental Fig. S4E). At the 300 bp peak, SINE/*Alu* elements, primarily the *Alu*Y subclass, were predominant in INS, comprising 43.9% of the total (Pearson's chi-squared test, *P*-value = 3 × 10^−10^) (Supplemental Fig. S4F). This observation may be attributed to the *Alu*Y subfamily, the youngest and most active member of the *Alu* family (Witherspoon et al. 2010; Hormozdiari et al. 2011), whose high activity has been identified in both cancer and healthy populations (Batzer and Deininger 2002; Wu et al. 2021; Zeng et al. 2025).

Somatic SVs breakpoints were nonuniformly distributed across the genome, with significant enrichment in repetitive elements (TE enrichment score: 0.85; repeat enrichment score: 10.20; nonrepetitive regions enrichment score: 0.82). Hyperrepetitive regions (including satellite, simple repeats, low complexity regions, and SVA elements) showed substantial enrichment (mean enrichment score > 6.56, Fisher's exact test, *P*-value < 0.05) (Fig. 4D). Notably, somatic DELs and INSs were more frequently observed in centromeric satellite sequences (enrichment score > 6.4, Fisher's exact test, *P*-value < 0.05), whereas larger SVs, such as INVs and DUPs, were underrepresented in these regions (enrichment score < 0.54). This pattern may be because of satellite sequences’ susceptibility to replication fork slippage or mispairing, combined with the compact chromatin structure of centromeric regions that limits DNA accessibility and favors conserved repair mechanisms, reducing the occurrence of large-scale SVs (Kashi and King 2006). Sequence annotation revealed that >60% of INSs inserted in satellite/center, satellite, and simple repeat regions harbored inserted sequences identical to flanking repeats (Supplemental Fig. S4G). A representative case in simple repeats demonstrated a 12-fold amplification of the reference TTCT motif in tumor samples (Supplemental Fig. S4H), indicating that somatic INSs preferentially propagate existing repetitive architectures through replication- or repair-associated templated insertion.

To elucidate the regulatory consequences of somatic SVs, we performed an enrichment analysis of SV breakpoints across functional genomic regions. Somatic INSs, DUPs, DELs, and INVs exhibited significant enrichment within proximal transcriptional regulatory domains (Fig. 4E), particularly in promoter-proximal (2.5 kb) and termination regulatory regions (downstream 2.5 kb), suggesting that these SVs may alter gene expression through *cis*-regulatory interference (Cosenza et al. 2022). Specifically, INS and BND breakpoints were broadly localized to *cis*-regulatory elements, with the strongest enrichment observed at CTCF-binding sites (enrichment score > 3.19, Fisher's exact test, *P*-value < 0.05) (Supplemental Fig. S4I), suggesting potential disruption of topologically associating domain boundary. In contrast, DUP and DEL breakpoints preferentially disrupted enhancers and promoters near transcription/termination sites, suggesting they modify the regulatory relationships between elements and genes (enrichment score > 1.40, Fisher's exact test, *P*-value < 0.05) (Supplemental Fig. S4I).

### Identification of high-frequency somatic SV regions in LSCC

To detect recurrent somatic SVs across genome, we performed a nonoverlapping sliding window approach for sample carrier rate quantification. We identified 226 high-frequency SV regions (sample carrier rate > 26%) exhibiting nonrandom chromosomal distribution (Fig. 5; Circos plot; Krzywinski et al. 2009) and significant colocalization with known tumor-related genes (chi-squared test, *P*-value < 0.05) (Supplemental Table S5; Tate et al. 2019). To assess robustness, we performed orthogonal validation using an independent SV caller (Severus) on the same data set. Notably, two INS hotspots identified by SomaGauss-SV were concordantly confirmed by Severus, underscoring their biological relevance beyond algorithmic artifacts (Supplemental Fig. S5).

**Figure 5.**
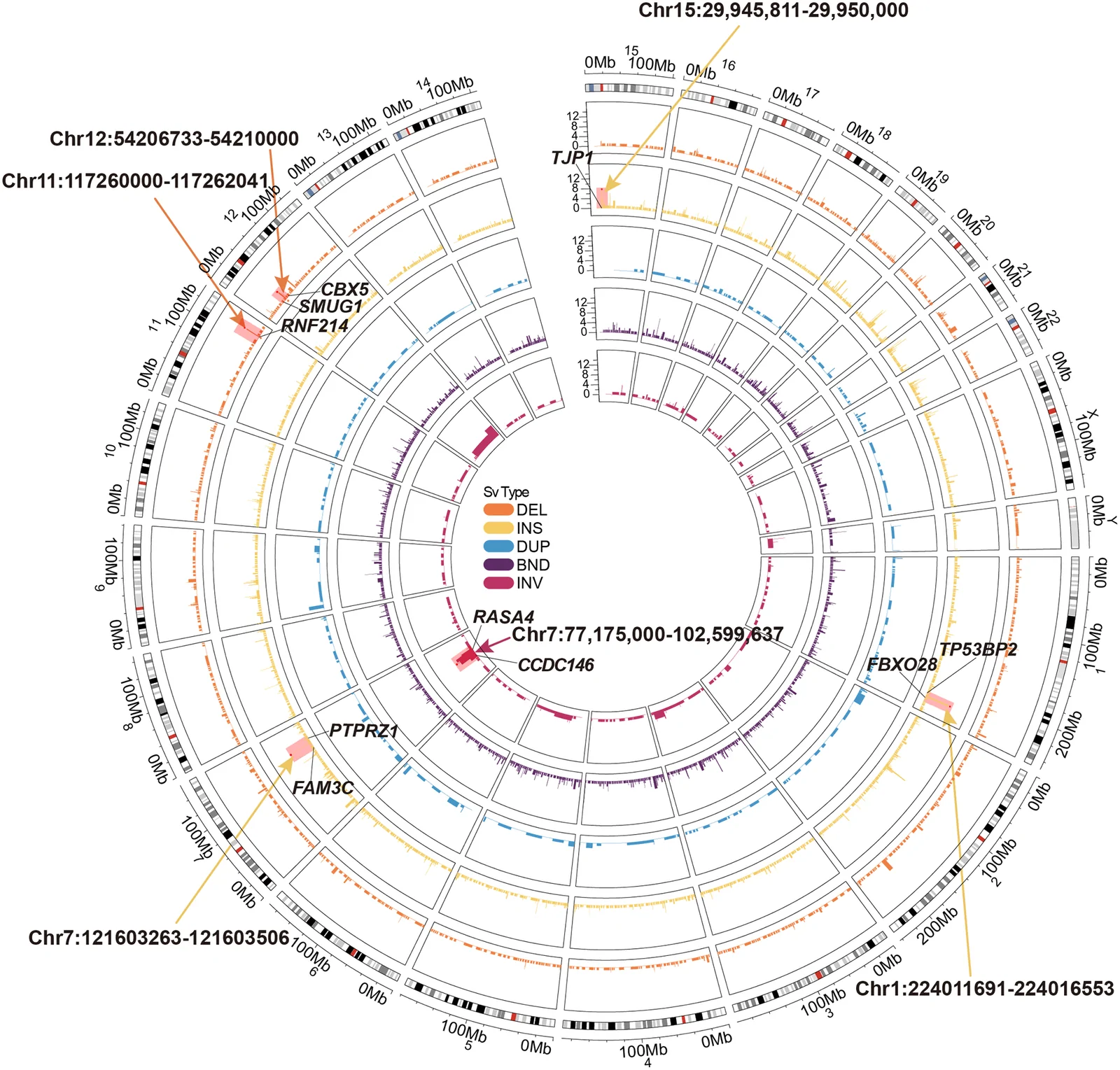
Circos plot of genome-wide sample enrichment. The tracks in the figure represent the enrichment of different SV types in the samples, from the *innermost* to the *outermost* being INV, BND, DUP, INS, and DEL. The plot highlights regions with the top two sample counts for INS and DEL that may affect genes, the top three regions for INV sample counts, and regions intersecting with those reported in previous studies.

We further characterized two high-frequency somatic INS regions, Chr 7: 121,603,263–121,603,506 (shared by nine LSCC samples) (Supplemental Fig. S6A) and Chr 15: 29,945,811–29,950,000 (shared by seven LSCC samples) (Supplemental Fig. S6B), which overlapped with recurrent repeat expansions (rREs) previously identified in the TCGA pancancer cohort (Erwin et al. 2023). The former INSs aligned with a rRE specifically detected in lung squamous cell carcinoma, whereas the latter corresponds to a rRE characteristic of prostate cancers. The inserted sequences of these somatic INSs matched the motifs described in previous study, suggesting that these somatic INSs have the potential to serve as pancancer biomarker across multiple malignancies. Moreover, we identified several high-frequency DELs that may influence nearby genes by disrupting regulatory elements. For instance, the region Chr 11: 117,260,000–117,262,041 harbored somatic DELs (235–353 bp) in six tumors (Supplemental Fig. S6C). This region is located within an intron of *RNF214* and overlapped with an enhancer element predicted by ENCODE (Moore et al. 2020). This enhancer is predicted to regulate *RNF214* expression in PC-3 prostate cancer cells (Moore et al. 2020). RNF214 is an E3 ubiquitin ligase. Its overexpression promotes tumor cell proliferation, migration, and invasion (Lin et al. 2024). Additionally, in Chr 12: 54,206,733–54,210,000, somatic DELs (163–178 bp) in five samples overlapped with an enhancer predicted to regulate *SMUG1* expression in Panc1 pancreatic cancer cells (Supplemental Fig. S6D; Moore et al. 2020). *SMUG1* is involved in base excision repair and genomic stability maintenance (Iakovlev et al. 2019). High *SMUG1* expression correlates with poorer survival in the TCGA HNSCC cohort (log-rank test, *P*-value = 0.003) (Supplemental Fig. S6E).

We also identified eight somatic INV shared by more than four samples (Supplemental Fig. S6F). The most frequent event occurred in region Chr 7: 77,175,000–102,599,637 (nine of the 15 samples). This interval encompasses 197 protein-coding genes, including 11 annotated proto-oncogenes. Pathway enrichment analysis revealed significant enrichment for the cytochrome P450 metabolic pathway (*q* < 0.05), which includes genes within this region such as *CYP3A5* and *CYP3A7*. Notably, CYP family enzymes can be induced by polycyclic aromatic hydrocarbons in tobacco smoke, enhancing the metabolic activation of procarcinogens and thereby promoting tumorigenesis (Zevin and Benowitz 1999). Analysis of INV breakpoints showed clustering at similar genomic positions in seven of the nine positive samples, resulting in INV fragments of 18–30 Mb. These clustered into two distinct groups: LSCC1, -3, -6, -14 and LSCC5, -7, -11 (Supplemental Fig. S7). Of particular note, the breakpoints in the first group (LSCC1, -3, -6, -14) localized within an intron of *CCDC146* and exon 7 of *RASA4* (Supplemental Fig. S7). *RASA4* encodes a calcium-dependent GTPase-activating protein that inactivates RAS and suppresses the MAPK pathway, functioning as a tumor suppressor. Its loss of function is implicated in malignant progression across various cancers (Prior et al. 2020; Chen et al. 2021). Because large INVs can disrupt gene architecture (Xu et al. 2023), this recurrent event may contribute to LSCC pathogenesis by altering *RASA4* transcription or function.

### Analysis and validation of a somatic SRE in 71.8% of LSCC patients

A somatic INS, observed in 66% (10/15) of LSCC tumors, was the most frequent recurrent SV in our study (Fig. 6A). It was located at Chr 1: 224,011,691–224,016,553, a simple repeat region annotated by RepeatMasker (Tarailo-Graovac and Chen 2009). The region initially consisted of a short tandem repeat with the (AATGG) repeat motif in normal samples, whereas the somatic alteration in nine of ten LSCC samples involved an expansion of this pattern, specifically a simple repeat expansion (SRE) (Fig. 6B). The LSCC11 sample differed, harboring Human Satellite II (HSATII; a satellite DNA derived from the (CATTC)n repeat) (Altemose et al. 2022) as its repeat motif. The somatic SREs exhibited low supporting read ratios in six samples (10%–30%), whereas four exhibited stronger support (>50%) (Supplemental Fig. S8A). SRE lengths varied among tumors (median: 581 bp) (Fig. 6C; Supplemental Fig. S8A). LSCC15 had a 3610 bp INS, whereas other samples showed peak SRE lengths at ∼450 bp (mean: 517 bp; SD: 382 bp). Additionally, two patients harbored multiple INS of different lengths. For example, the LSCC1 sample harbored 390 bp, 650 bp, and 1030 bp INSs (Supplemental Fig. S8A; Fig. 6A), indicating more tumor heterogeneity.

**Figure 6.**
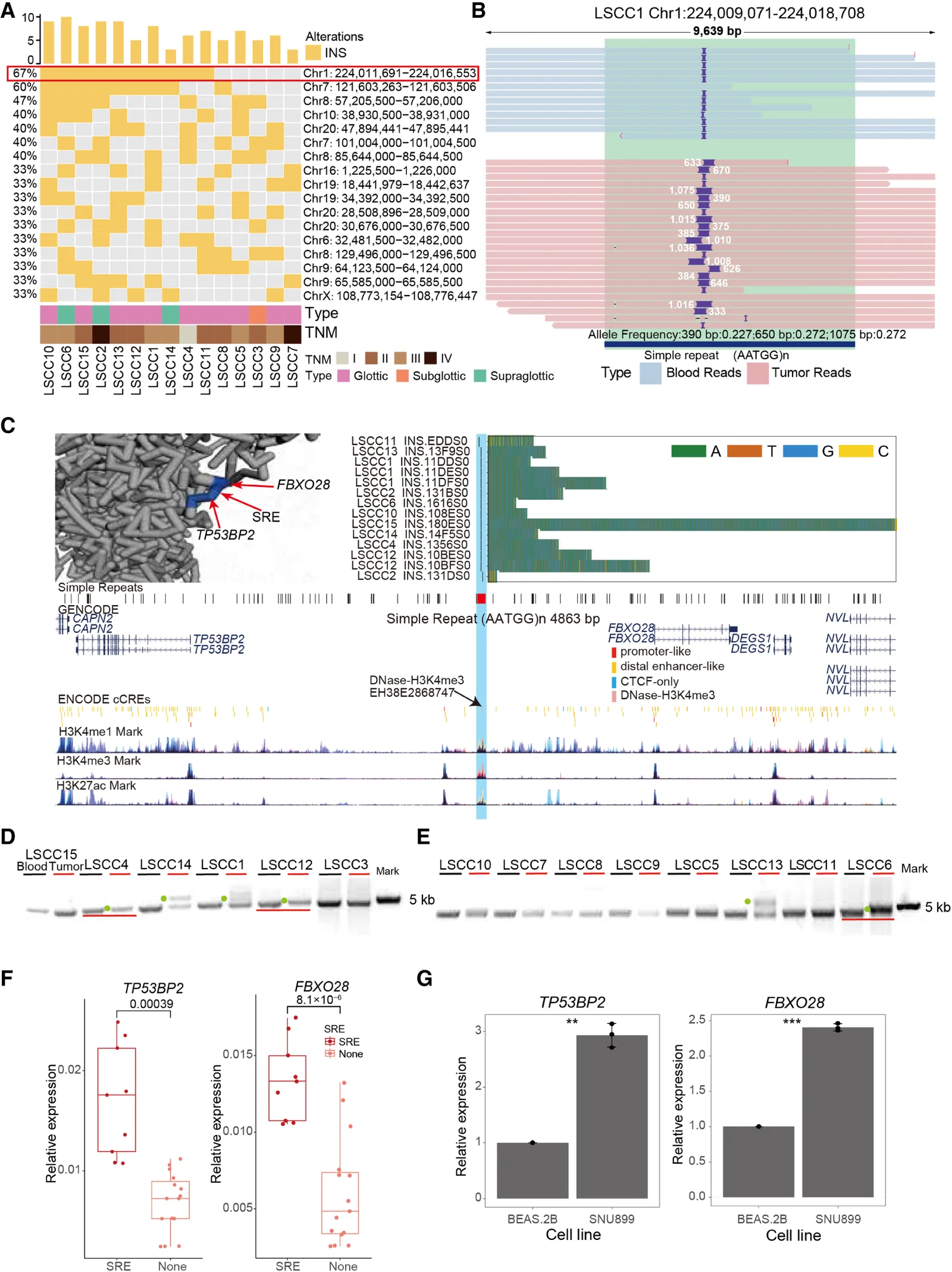
Overview of somatic SRE shared by 66% of samples. (*A*) Oncoplot of INS sample enrichment regions. Rows represent samples, and columns represent regions. (*B*) IGV plot of somatic SREs (LSCC 13, Chr 1: 224,009,042–224,018,679). Light blue represents reads from blood samples, pink represents reads from tumor samples, and dark blue indicates the simple repeat region with the AATGG motif. (*C*) Browser plot of somatic SREs (Chr 1: 224,009,042–224,018,679) illustrating the following epigenomic signals: a stacked bar chart of SRE positions and inserted sequences, with different colors representing different bases; the plot concludes with the AATGG motif of the repeat, the ENCODE-annotated gene transcript, and candidate *cis*-regulatory elements (cCREs) predicted by ENCODE. Also included are H3K4me1, H3K4me3, and H3K27ac marks predicted by ENCODE. In the *upper left* corner, the predicted 4D nucleosome structure around the LSCC-enriched SRE, *TP53BP2*, and *FBXO28* is displayed. (*D*) Horizontal gel electrophoresis images showing DNA fragments from blood and tumor tissues of LSCC samples sequenced using nanopore technology. Each sample's blood and tumor are labeled separately, and green dots and red lines mark the longer bands in the tumor relative to the blood. (*E*) Horizontal gel electrophoresis images showing DNA fragments from blood and tumor tissues of LSCC samples sequenced using nanopore technology. Each sample's blood and tumor are labeled separately, and green dots and red lines mark the longer bands in the tumor relative to the blood. (*F*) Relative expression levels of *TP53BP2* and *FBXO28* determined by QCPCR in SRE and no SRE clinical samples (*t*-test). (*G*) Relative expression levels of *TP53BP2* and *FBXO28* determined by QCPCR in the BEAS 2B and SNU899 cell lines. *t*-test, (*) *P*-value < 0.05, (**) *P*-value < 0.01, (***) *P*-value < 0.001.

To validate the presence of somatic SRE identified through LRS WGS, target PCR amplification of the SRE region was performed on DNA from available 14 paired tumor and blood samples (including nine tumors with INSs identified by LRS WGS). Gel electrophoresis indicated that six of nine tumors displayed longer fragments compared with blood samples (Fig. 6D,E). The remaining three samples presented challenges owing to a low SRE supporting ratio (20% and 30% for LSCC10 and LSCC11) and a long insertion length in LSCC15 (3610 bp) (Supplemental Fig. S8A), complicating the gel electrophoresis validation. Despite these challenges, our results confirm the authenticity and reliability of these high-frequency somatic SRE.

To assess the prevalence of the high-frequency somatic SRE in LSCC tumors, we further collected 24 paired LSCC samples for PCR validation and ONT sequence (Supplemental Figs. S8B–G, S9–S19). Gel electrophoresis and LRS data of the target PCR products revealed that 18/24 LSCC samples carried the somatic SRE with an (AATGG) repeat motif, with insertion lengths ranging from 130 bp to 3693 bp (Supplemental Figs. S9–S19; Supplemental Table S6). Furthermore, this SRE was detected in the peritumoral tissues of 15/21 (71.42%) LSCC tumors (Supplemental Fig. S8G; Supplemental Table S6). Together, the high prevalence of this somatic SRE in peritumoral tissue suggested it is an early genomic event, whereas its significantly elevated allele frequency in tumor compared with peritumoral tissue (*t*-test, *P*-value = 0.0046) (Supplemental Fig. S8H) indicates positive selection of the SRE-harboring clone during tumor evolution.

To further explore the functional role of the high-frequency somatic SRE, we analyzed the chromatin environment surrounding the SRE. In the A673, A549, and SK-N-MC cell lines, the SRE region overlapped with DNase-H3K4me3 signals, which are known to mark transcriptional start sites (Fig. 6C; Moore et al. 2020). Although the target genes regulated by this DNase-H3K4me3 mark have not been extensively characterized, Hi-C data from the hypopharyngeal cancer FaDu cell line suggested that the region resides within the same topological domain (TAD) as the nearby coding genes *TP53BP2* and *FBXO28* (Supplemental Fig. S20A). Furthermore, data from the 4D Nucleosome Consortium (Zhu et al. 2022) supported the presence of three-dimensional chromatin interactions between this DNase-H3K4me3 region and the genes *TP53BP2* and *FBXO28* (Fig. 6C). Critically, analysis of matched TCGA LSCC samples confirmed that, among genes near the SRE, the expression of *FBXO28* and *TP53BP2* exhibited the most significant tumor-specific upregulation (*t*-test, *P*-value < 0.001) (Supplemental Fig. S20B). These convergent lines of evidence raised the possibility that this somatic SRE may alter the regulation of these two genes. Consistent with this hypothesis, quantitative real-time PCR (qPCR) analysis of our clinical LSCC samples revealed that tumors harboring the SRE exhibited significantly higher expression levels of both *TP53BP2* and *FBXO28* compared with SRE-negative tumors (*t*-test, *P*-value = 0.00039 and *P*-value = 8.1 × 10^−6^) (Fig. 6F), directly linking the presence of the SRE to upregulated expression of its putative target genes. *TP53BP2* encodes the apoptosis-stimulating protein, which interacts with P53 family members to modulate cell apoptosis and growth (Huo et al. 2023). *FBXO28*, which encodes a ubiquitin ligase, has been reported to be aberrantly overexpressed in human epithelial cancer cell lines (Song et al. 2024).

We next examine the regulatory effect of the high-frequency somatic SRE on gene expression using cell line models. With gel electrophoresis, we identified longer fragments in two LSCC cell lines (SNU46 and SNU899) compared with a normal human bronchial epithelial cell line (BEAS-2B) (Supplemental Fig. S8B). Nanopore sequencing of the PCR products further confirmed that both LSCC cell lines harbored an expected INS with AATGG repeat motif. Specifically, SNU46 and SNU899 carried homozygous INSs with lengths of 165 bp and 240 bp, respectively (Supplemental Fig. S20C,D). In contrast, no significant sequence length variation was observed in BEAS-2B cells compared with the reference genome. We then selected the SNU899 cell line, which has a homozygous SRE with longer insertion, and the BEAS-2B cell line for qPCR to assess whether the SRE affects the expression levels of *TP53BP2* and *FBXO28*. Consistent with findings in primary tumors, we found that *TP53BP2* and *FBXO28* expression levels were significantly higher in the SNU899 cell line than in the BEAS-2B cell line (*t*-test, *TP53BP2*: *P*-value < 0.01; *FBXO28*: *P*-value < 0.001) (Fig. 6G). To rule out tissue-specific baseline differences as the cause, we analyzed normal tissue data from TCGA (LSCC vs. LUSC) and cell line data from DepMap. This confirmed that expression of these genes in laryngeal contexts was not inherently higher than in pulmonary contexts, supporting the SRE's specific role in upregulation (Supplemental Fig. S20E,F). To directly test whether the SRE alters local chromatin architecture, we performed 3C-PCR with an anchor primer (Anchor-S) placed downstream from the simple repeat region where the SRE occurs. In SNU899 (SRE-positive), the Anchor-S locus exhibited strong, specific spatial proximity to the F1 regulatory site upstream of *FBXO28*. In BEAS-2B (SRE-negative), the same Anchor-S locus showed a markedly weaker interaction with *FBXO28*-F1. Instead, its predominant interactions were with sites near *TP53BP2* (T3/T8) (Supplemental Fig. S20G). This reciprocal pattern demonstrates that the presence of the SRE is associated with a distinct three-dimensional chromatin conformation, specifically promoting spatial proximity to the regulatory region of *FBXO28*. Together, these findings suggest that the somatic SRE may upregulate *TP53BP2* and *FBXO28* expression in LSCC by altering the local three-dimensional chromatin architecture.

## Discussion

Somatic SVs are critical hallmarks of tumorigenesis (Cosenza et al. 2022). However, technical limitations of SRS in sensitively detecting somatic SVs, compounded by the tissue heterogeneity characteristic of HNSCC, have left the landscape of high-frequency somatic SVs in LSCC underexplored. This gap in knowledge has hindered the identification of potential LSCC-specific molecular biomarkers for effective cancer management. To address these challenges, we developed SomaGauss-SV, a LRS-optimized computational framework for somatic SV detection. Benchmarking against established tools revealed that SomaGauss-SV offered a superior balance of sensitivity and specificity, with an F1-score improvement of 11.3%–33.9%. SomaGauss-SV excels in two key areas: (1) it reduces alignment errors in repetitive genomic regions through a multistep filtering approach, and (2) it accurately distinguishes true somatic INSs, DUPs, and DELs in the presence of size-variable INSs/DUPs/DELs using a Gaussian mixture model (GMM). Despite these advances, significant technological challenges persist. Current LRS platforms still struggle with resolving complex breakpoint architectures, particularly those involving nested rearrangements and ultralong SVs (>100 kb) (Keskus et al. 2026), limitations shared across existing detection tools, including SomaGauss-SV. Furthermore, the current benchmarking landscape relies heavily on consensus-based “gold standard” sets derived from multiple sequencing platforms and computational tools rather than orthogonal experimental validation. This approach may introduce methodological bias, especially for complex SVs, which remain difficult and costly to validate experimentally, resulting in limited and potentially incomplete ground-truth data sets (e.g., the COLO829 benchmark provides only 68 gold-standard SVs) (Espejo Valle-Inclan et al. 2022). We also observed that the choice of benchmarking strategy (minda vs Truvari) itself significantly impacts performance evaluation. There is currently no consensus in the field on whether SV type should be incorporated as a matching criterion during benchmarking. Although classifying complex SVs can be ambiguous, applying a consistent typing standard is necessary for the robust evaluation of simple somatic variants (e.g., INSs, DELs, DUPs), as type-agnostic matching may misclassify genuine events. However, recent innovations in high-throughput ultralong-read sequencing technologies (Wang et al. 2021) and graph-based or assembly-based long-read alignment algorithms (Li and Durbin 2024) offer promise for overcoming these obstacles.

We systematically analyzed the LRS WGS data of 15 paired LSCC samples using SomaGauss-SV, revealing the distribution and sequence features of somatic SVs. We identified a dose-dependent positive correlation between smoking intensity and somatic DEL burden in LSCC. Previous studies indicated tobacco exposure is the primary driver of HNC, with tobacco users exhibiting higher frequencies of somatic SNVs and indels (Alexandrov et al. 2016; Torrens et al. 2025). Notably, tobacco-related mutational signatures show higher burdens and frequencies in laryngeal cancers compared with other HNC subsites (Alexandrov et al. 2016; Torrens et al. 2025). However, the link between tobacco and somatic SVs remains less clearly defined (Alexandrov et al. 2016; Jethwa and Khariwala 2017; Yoshida et al. 2020; Torrens et al. 2025). Our study fills this gap by demonstrating the role of chemical carcinogens in chromosomal instability. Specifically, we suggested that the accumulation of somatic DEL may result from the synergistic effects of multiple carcinogens in tobacco smoke. DNA adducts from polycyclic aromatic hydrocarbons and nitrosamines can block replication forks and cause DNA double-strand breaks (Yoshida et al. 2020). These breaks, when repaired poorly, may lead to chromosomal deletions. Also, free radicals and reactive oxygen species from tobacco can cause more DNA damage through oxidative stress, promoting deletions during faulty repair (Yoshida et al. 2020). Tobacco components might also disrupt cell division or cycle regulation (Caliri et al. 2021), increasing chromosomal instability. Although high expression of *CYP1A1*, the key enzyme involved in the metabolism of benzopyrene and nitrosamines, has been proposed as a potential cause of the elevated tobacco-related mutation signatures in laryngeal tumors (Yamazaki et al. 1992; Degawa et al. 1994; Hecht and Hatsukami 2022), the specific tobacco carcinogens responsible for the observed DEL burden remain to be identified in vitro. Clinically, DEL burden could serve as a potential biomarker for risk stratification and therapeutic target screening in LSCC. Further investigation into high-frequency deletions associated with smoking may provide evidence for preventive interventions targeting high-dimensional genomic regions. For example, somatic DEL might disrupt CTCF binding in 3D chromatin architecture, influencing the expression of immune genes like *HLA-DRB5* and *HLA-DRA*. However, the limited sample size in this study restricts the generalizability of the observed association between somatic SV burden and smoking. Other potential confounders, such as alcohol consumption and dietary factors, may also influence the results. Future studies with larger cohorts and the incorporation of multivariable models or in vivo/vitro experiments are needed to confirm these findings.

In addition, 226 high-frequency SV loci were identified, exhibiting selective advantages during tumor clonal evolution. Cross-cancer comparison analysis revealed that high-frequency SVs at Chr 7: 121,603,263–121,603,506 and Chr 15: 29,945,811–29,950,000 overlap with hotspot somatic repeat expansions reported in pancancer studies (Erwin et al. 2023), suggesting their involvement in conserved carcinogenic pathways across various cancer types. Significant CNV regions in laryngeal cancer from the TCGA database also overlap with the high-frequency large DELs, DUPs, and INSs, further validating the unique strength of LRS technology in resolving multiscale genomic rearrangements. A key issue to explore further is how to define the boundary between CNVs identified through sequencing depth and DELs/INSs/DUPs detected by long reads. Furthermore, we investigated co-occurring SV patterns within individual reads, which may represent complex structural rearrangements. For instance, in sample LSCC1, a single read simultaneously contained an insertion of a simple repeat (AT), a deletion within a LINE/L1 element, and an insertion of a SINE/*Alu*Y sequence (Supplemental Fig. S21). In LSCC3, we identified an event structurally resembling an “inverted duplication” as previously reported (Schloissnig et al. 2025), involving both INV and DUP on the same allele (Supplemental Fig. S22). These examples illustrate the capacity of long-read sequencing to resolve complex, multivariant rearrangements.

Notably, analysis of both WGS and an independent validation cohort identified a somatic high-frequency SRE in 71.79% (28/39) of LSCC patients. Among the 21 patients with available peritumoral tissue, the SRE was detected in both tumor and matched peritumoral samples in 15 individuals. The allele frequency of the SRE was significantly higher in tumor tissue than in peritumoral tissue, suggesting that this alteration may arise early during tumorigenesis and undergo subsequent positive selection. We also observed heterogeneity in SRE lengths within the same tumor, indicative of intratumoral diversity and potentially distinct selective advantages associated with different expansion sizes. This dynamic evolution was particularly evident in a patient with lymph node metastasis, in which the SRE was detected in the primary tumor (400 bp), peritumoral tissue (125 bp), and metastatic lymph node (200 bp). This suggested that the SRE evolves dynamically throughout tumor progression and metastasis, conferring varying selective advantages in different microenvironments. However, the mechanisms linking SRE length variation to phenotypic diversity in tumor cells require further investigation.

The recurrent somatic SRE identified in this study holds significant potential as a biomarker for LSCC. Integrating 3C-PCR and qPCR results from our cell line models, we propose a hypothesis in which the SRE may regulate the expression of neighboring genes by altering local three-dimensional chromatin architecture. Specifically, the SRE-mediated sequence elongation could increase spatial proximity between a distal regulatory element and the promoters of *TP53BP2* and *FBXO28*, thereby enhancing their transcription. Although external data suggested that baseline expression of these genes in laryngeal contexts is not inherently higher compared with pulmonary tissues, the inherent tissue-of-origin heterogeneity between the lung epithelial cell line BEAS-2B and the LSCC-derived SNU899 line warrants caution in interpreting cross-tissue comparisons. Nevertheless, the functional relevance of these genes in cancer is well supported: Elevated *FBXO28* expression is linked to poor prognosis in ovarian cancer, in which it activates the TGFB1/SMAD2/3 pathway to promote proliferation and invasion (Song et al. 2024). *TP53BP2*, a member of the ASPP family, regulates apoptosis, proliferation, and autophagy, and its upregulation correlates with poor prognosis in colorectal cancer and other malignancies (Huo et al. 2023). To definitively test our mechanistic hypothesis, future studies employing CRISPR-Cas9 to delete the SRE in relevant models would be essential to directly assess its impact on *TP53BP2* and *FBXO28* expression and downstream phenotypes, thereby excluding the influence of tissue-of-origin heterogeneity.

In conclusion, the bioinformatic pipeline developed in this study achieves an optimal balance between sensitivity and specificity, offering a robust tool for future research on tumor somatic SVs. This study represents the first comprehensive genome-wide survey of somatic SVs in LSCC using LRS, with the generated WGS data and identified SVs offering a valuable resource for clinical research in the field.

## Methods

### Sample and patient information collection

This study involved 39 patients with LSCC who underwent surgical resection at West China Hospital, Sichuan University. Tumor samples and matched blood samples were collected from these patients during surgery and venipuncture. Throughout the tissue collection and utilization process, this study strictly adhered to the Declaration of Helsinki and received formal approval from the ethics review committee of West China Hospital, Sichuan University. All patients participating in this study have signed written informed consent forms. In addition, key clinical information, including clinical staging, smoking history, age, and gender, was thoroughly documented for each patient.

### Cell culture

Human cell lines, obtained from Procell (Wuhan Pricella Biotechnology) were utilized in this study. The cells were grown in DMEM medium (Gibco C11995500BT) or RPMI 1640 medium (Gibco C11875500BT), supplemented with 10% fetal bovine serum (FBS) and 100 U/mL penicillin–streptomycin (P/S). All cell lines were maintained at 5% CO_2_ and 37°C. Periodic mycoplasma testing was conducted using the mycoplasma detection kit (Vazyme D101). Cells were passaged at 70%–90% confluency with 0.25% trypsin-EDTA (Gibco 25200-072).

### Gene expression, chromatin conformation, and genomic validation assays

For gene expression analysis, total RNA was extracted, reverse-transcribed, and quantified by qRT-PCR using gene-specific primers. To investigate chromatin interactions between the SRE region and target genes (*FBXO28* and *TP53BP2*), Chromatin Conformation Capture (3C) libraries were generated from cross-linked SNU899 cells via EcoRI digestion and intramolecular ligation, and specific interactions were quantified by 3C-qPCR. Additionally, genomic DNA was used to validate the presence of the SRE in clinical samples and cell lines by PCR amplification followed by Sanger sequencing. The primer sequences are listed in the oligonucleotide table (Supplemental Table S7). Detailed protocols, including all primer sequences and reaction conditions, are provided in the Supplemental Methods.

### Nanopore sequencing library preparation

For WGS, high-molecular-weight genomic DNA was extracted and used for library preparation with Oxford Nanopore Technologies (ONT) ligation kits (SQK-LSK109 or SQK-LSK114), followed by sequencing on PromethION flow cells. For targeted sequencing of the SRE locus, PCR amplicons were prepared into libraries using either the ONT or Qitan Tech nanopore sequencing platforms and sequenced on GridION X5 or QNome-3841 instruments, respectively. Detailed protocols for library preparation, including specific reagents, reaction conditions, and purification steps, are provided in the Supplemental Methods.

### Preprocessing of long-read sequencing data

For data preprocessing, raw long-read sequencing data were quality-filtered and assessed using NanoFilt (version 2.8.0) (Lee et al. 2021) and NanoPlot (version 1.39.0) (De Coster and Rademakers 2023), respectively. Filtered reads were aligned to the hg38 reference genome using minimap2 (version 2.26) (Li 2018). Resulting SAM files were converted, sorted, and read-tagged using SAMtools (version 1.9) (Danecek et al. 2021) to generate final BAM files. For the analysis of SRE-targeted PCR products, a similar preprocessing pipeline was applied, with specific length filtering to ensure complete spanning of the repeat region. The processed alignments were visualized using the Integrative Genomics Viewer (IGV) (Robinson et al. 2011). Detailed parameters and steps are provided in the Supplemental Methods.

### Acquisition and preprocessing of nanopore sequencing data and gold-standard somatic SV data from cell lines

According to the reference (Keskus et al. 2026), we downloaded the nanopore sequencing data for five paired cell lines, HCC1395/HCC1395BL, H1437/H1437BL, H2009/H2009BL, HCC1937/HCC1937BL, and HCC1954/HCC1954BL, as well as the corresponding gold-standard sets of somatic SVs for these cell lines. To ensure the accuracy of the analysis, we filtered the gold-standard data, retaining only those somatic SVs that could be detected by the nanopore sequencing platform, totaling 4058, including DEL 1493, INS 906, DUP 385, INV 48, and BND 1226. Subsequently, following the standard workflow for nanopore sequencing data preprocessing, we aligned and processed these data, ultimately obtaining BAM files with “blood” and “tumor” tags, respectively. The LRS WGS data and benchmark SV sets of five cell line pairs were downloaded from the NCBI BioProject database (https://www.ncbi.nlm.nih.gov/bioproject/) under accession number PRJNA1086849.

### SomaGauss-SV workflow

The SomaGauss-SV workflow for somatic SV detection involved four key stages. First, merged tumor–normal BAM files were analyzed with Sniffles2 (version 2.0.6) (Smolka et al. 2024) to call candidate variants. Second, a type-specific screening strategy was applied: Candidate DEL were validated using a GMM on locally extracted reads, whereas candidate INSs and DUPs were reanalyzed using Straglr (Chiu et al. 2021), a length-based Gaussian modeling tool optimized for repetitive regions. Third, for clinical samples, variants were filtered against a PON derived from Chinese population SV data (Wu et al. 2021). using Jasmine (version 1.1.5) (Kirsche et al. 2023) to remove common germline polymorphisms. Finally, complex variants (INVs and BNDs) were manually validated through visualization using the IGV (Robinson et al. 2011). Detailed parameters and scripts are provided in the Supplemental Methods.

To validate our foundational tool selection and demonstrate the incremental value of each step in the SomaGauss-SV pipeline, we conducted systematic benchmarking. First, Sniffles2 was empirically selected over cuteSV (Jiang et al. 2020) as the initial variant caller owing to its superior performance (mean F1-score: 68.7% vs. 53.4%) (Supplemental Fig. S23A,B). Subsequently, a stepwise comparison of three workflows: (1) original Sniffles, the merged VCFs obtained by running Sniffles2 alone; (2) filter reads, the SV set after further filtering out variants with any supporting reads in the matched normal (blood) sample; and (3) SomaGauss (full pipeline): The final output after applying our GMM-based filtering step to the “filter reads” set. It significantly enhanced precision (from 14.5% to 71.51%) and F1-scores (overall +39.63%, with a more pronounced improvement of +47.86% in challenging VNTR regions) by effectively removing subtle false positives, particularly those arising from alignment errors in repetitive sequences. This confirms that each component of SomaGauss-SV adds substantial value, culminating in a highly accurate and robust somatic SV detection workflow (Supplemental Fig. S23C–F).

### Benchmarking and performance evaluation of SV detection tools

For benchmarking comparisons, somatic SVs were independently detected using three established calls, nanomonsv (Shiraishi et al. 2023), Severus (Keskus et al. 2026), and SVision-pro (Wang et al. 2025), following each call's recommended parameters and workflow. The performance of all callers was then evaluated against the gold-standard variant set using two evaluation tools: Truvari (English et al. 2022) and minda (Keskus et al. 2026). Detailed command-line parameters are provided in the Supplemental Methods.

### Evaluating software runtime efficiency

To evaluate the runtime efficiency of each software tool, we used the command “/usr/bin/time -v” followed by the respective software command line to measure both execution time and maximum memory usage. Most tools involve multiple distinct steps in their workflows; for example, Severus includes separate phases for SNP calling and phasing of the normal BAM, haplotagging of normal and tumor BAMs, and somatic SV calling, whereas SomaGauss-SV comprises steps such as merging BAM files, calling SVs from the merged BAM, and running Straglr. We adopted the following practical approach to summarize resource consumption. For total elapsed real time, we summed the elapsed real time from each step of a given tool to represent its overall runtime. For maximum resident set size, we recorded the maximum resident set size observed across all steps as the tool's peak memory footprint. This method provides a consolidated and realistic view of computational resource requirements for each somatic SV detection pipeline under evaluation.

### Variant sequence annotation and genomic context analysis

For sequence annotation and refinement, the consensus sequences of somatic INS were first corrected using Iris (version 1.0.4) (Kirsche et al. 2023) and then annotated for repetitive elements using RepeatMasker (version 4.1.2) (Tarailo-Graovac and Chen 2009). To characterize the genomic context of somatic DEL, their breakpoint regions were intersected with a RepeatMasker-annotated genome using BEDTools (version 2.30.0) (Quinlan and Hall 2010) to identify overlaps with repetitive sequences. Detailed parameters for each tool are provided in the Supplemental Methods.

### Genomic enrichment and recurrence analysis of somatic SVs

To assess genomic enrichment of somatic SVs, we performed two complementary analyses. First, SV breakpoints were intersected with functional genomic elements (genes and candidate *cis*-regulatory elements from UCSC) (Lee et al. 2020) using BEDTools. Enrichment fold and statistical significance were calculated using the Fisher's exact test based on a contingency table of variant counts within and outside these elements relative to genomic background. Second, to address alignment ambiguity in repetitive regions (Supplemental Fig. S1; Ahsan et al. 2023; Sahlin et al. 2023), we constructed a nonuniform window BED file by combining annotated simple repeat regions with 500 bp/5 kb windows in nonrepetitive regions. Somatic SV loci were then intersected with these windows using BEDTools to calculate sample carrier rates, and adjacent regions with shared variants were merged prior to downstream analysis. Detailed procedures are provided in the Supplemental Methods.

### Code availability

SomaGauss-SV is available at GitHub (https://github.com/XuYan000131/SomaGauss-SV) and as Supplemental Code.

## Data access

The BAM files generated in this study have been submitted to the Genome Sequence Archive (Zhang et al. 2025) in the National Genomics Data Center (CNCB-NGDC Members and Partners 2025), China National Center for Bioinformation/Beijing Institute of Genomics, Chinese Academy of Sciences (GSA-Human; https://ngdc.cncb.ac.cn/gsa-human/) under accession number HRA017212.

## Supplemental Material

Supplement 1

Supplement 2

Supplement 3

Supplement 4
