# Supplementary material for: Nanopore sequencing identifies high-frequency somatic structural variations in laryngeal squamous cell carcinoma genomes
Source: Genome Res. 2026 Nov;36(11):2307–21. doi: 10.1101/gr.281046.125 (PMC13739014; doi:10.1101/gr.281046.125)
Supplement: Supplementary file 1 [file Supplemental_Methods.pdf]

Supplementary Methods for

Nanopore Sequencing Unveils Somatic Structural Variations as Biomarkers in Laryngeal squamous cell carcinoma Genomes

Xuyan Liu et al.

*Corresponding author. Email: danxie@scu.edu.cn

Contents

Real-Time quantitative PCR 2
3C library generation 2
3C-PCR analysis 3
Validation of the SRE insertion in tissues and cell lines using PCR and sanger sequencing 3
Oxford nanopore library preparation and sequencing for HMW gDNA 4
Oxford nanopore library preparation and sequencing for SRE PCR products 5
Library preparation for Qitan Tech nanopore sequencing 6
Long-read whole-genome sequencing data preprocessing 7
Analysis and visualization of nanopore sequencing data for PCR products 7
SomaGauss-SV workflow 8
Detection of somatic structural variations with nanomonsv 11
Detection of somatic SVs with Severus 11
Detection of somatic SVs with SVision-pro 12
Evaluating somatic SVs software 12
Correction and annotation of somatic INS consensus sequences 12
Annotation of somatic DEL sequences 13
Statistical analysis of enrichment site regions 14
Sample enrichment analysis 14

Real-Time quantitative PCR

Total RNA was extracted using TRIzol reagent (Invitrogen, 15596026CN) and reverse-transcribed into cDNA with PrimeScript FAST RT reagent Kit (Takara, RR092A) following manufacturer's protocols. Gene-specific primers (designed using NCBI and validated for specificity) were utilized with TB Green Premix Ex Taq II (Takara, RR820A) on a Bio-Rad system. All sequences were synthesized by Sangon (Chengdu, China) and are listed in the oligonucleotide table (Supplementary Table S7). Relative quantification was calculated using the $2^{-\Delta\Delta Ct}$ method with GAPDH normalization.

3C library generation

Here, 3C experiments were performed in SNU899 cell lines. Formaldehyde crosslinking and 3C library preparation were performed as described previously. Briefly, 1×107 cells were cross-linked with 1% formaldehyde for 10 minutes at room temperature and quenched with glycine. Cross-linked cells were lysed with 5 mL 1× cold lysis buffer (10 mM Tris-HCl, pH 7.5, 10 mM NaCl, 0.2% NP-40) containing 1× protease inhibitor (Beyotime, P1050) for 10 minutes on ice. Cell nuclei were pelleted and resuspended in 0.5 mL of 1.2× restriction enzyme buffer with 0.3% SDS, followed by incubation for 1 hour at 37 °C. Then, 1% Triton X-100 was added to the sample, and the sample was incubated for another 1 hour to sequester the SDS. Each sample was digested overnight with 400 U EcoRI (NEB, R0101T) restriction enzymes at 37 °C. To terminate the restriction digestion, 1.6% SDS (final concentration) was added, and the samples were incubated at 65 °C for 20 minutes. Ligation was carried out at 16 °C for 4 hours, followed by 30 minutes at room temperature in a 50 mL falcon tube containing 6.125 mL of 1.15× T4 ligation buffer, 1% Triton-X 100 (final concentration), and 100U T4 ligase (NEB, M0202T). The crosslinks were reversed with 300 μg Proteinase K (QIAGEN) at 65 °C overnight. The 3C samples were purified using phenol chloroform extraction and quantified by Qubit dsDNA HS

Assay (Thermo Fisher, Q33230).

3C-PCR analysis

The 3C-PCR primers were designed to detect the interactions between *FBXO28* and *TP53BP2* and the SRE-inserted promoter regions, respectively. The anchor primers were designed near the SRE-inserted region, while the test primers were designed around the EcoRI cutting sites in the regions of target gene. Each test primer was paired with the anchor primer. The primer sequences are listed in the oligonucleotide table (Supplementary Table S7). All PCRs were performed with TB Green Premix Ex Taq II (Takara, RR820A). Each 25 μL reaction consisted of 12.5 μL of TB Green Premix Ex Taq II (2X), 2 μL of 10 μM anchor primer, 2 μL of 10 μM test primer, and 100 ng of 3C DNA. The PCR protocol included an initial denaturation at 95 °C for 30 seconds, followed by 39 cycles of 95 °C for 5 seconds and 60 °C for 30 seconds. Negative controls (no-ligation) and replicate reactions were included. Interaction frequencies were quantified by qPCR and normalized to control loci.

Validation of the SRE insertion in tissues and cell lines using PCR and sanger sequencing

Genomic DNA was isolated from tumor tissues, blood and LSCC cell lines using the QIAamp DNA Mini Kit (QIAGEN, 51306), followed by purity and concentration assessment via NanoDrop and agarose gel electrophoresis. The forward and reverse primers were designed according to the 500 bp upstream and downstream sequences of the insertion position and synthesized by Sangon Biotech (Supplementary Table S7). PCR amplification was performed in a Thermal Cycler using Phanta Max Super-Fidelity DNA Polymerase (Vazyme, P505).Each 50-μL reaction consisted of 2× Phanta Max Buffer 25 μL, 2 μL 10 μM forward primer, 2 μL 10 μM reverse primer, and 50-150 ng DNA. PCR was performed as follows: an initial step of 3 minutes at 95 °C, 30 cycles of 10 seconds at 95 °C, 15 seconds at 58 °C and 2 minutes

30 seconds at 72 °C. The PCR products were sent to Sangon Biotech for Sanger sequencing.

Oxford nanopore library preparation and sequencing for HMW gDNA

High-molecular-weight (HMW) genomic DNA (gDNA) was extracted from tissue or blood samples using the MagAttract HMW DNA Kit (QIAGEN, 67563), and quantified using the Qubit® DNA Assay Kit and Qubit® 3.0 Fluorometer (Life Technologies, CA, USA). For library preparation, 9 samples (60%) were processed with the Ligation Sequencing Kit 1D (SQK-LSK109), and 6 samples (40%) with the Ligation sequencing DNA V14 kit (Oxford Nanopore Technologies, SQK-LSK114), following the manufacturer's protocols (Supplementary Table S7). For the 60% of samples undergoing library preparation with SQK-LSK109, each sample was subjected to DNA end repair by combining 2 μg gDNA with 6.5 μL of the corresponding Buffer, 2 μL of NEBNext FFPE DNA Repair Mix (NEB, M6630L), and 3 μL of NEBNext Ultra II End Prep Enzyme Mix (NEB, E7546L), and the volume was adjusted to 60 μL with nuclease-free water. The mixture was gently mixed by pipetting, then incubated at 20 °C for 5 minutes followed by 65 °C for 5 minutes. The End-prepped DNA was purified with 1× volume of AMPure XP beads (Beckman Coulter, A63881) and washed twice with 80% ethanol. For adapter ligation, the purified DNA was mixed with 25 μL of Ligation Buffer, 10 μL of NEBNext Quick T4 DNA Ligase (NEB, E6056L) and 5 μL of Adapter Mix, then incubated at 25 °C for 20 minutes. Subsequently, 0.4× volume of AMPure XP beads was added, and the DNA pellet was resuspended in 25 μL of Elution Buffer. The remaining 40% of samples were prepared following the library preparation steps outlined in the SQK-LSK114 protocol. Libraries prepared with SQK-LSK109 were loaded onto R9.4.1 flow cells, while those prepared with SQK-LSK114 were loaded onto R10.4.1 flow cells. Sequencing was performed on the PromethION platform in accordance with the manufacturer's instructions. Basecalling was conducted in batches using Guppy v3.2.8 with default parameters during sequencing.

Oxford nanopore library preparation and sequencing for SRE PCR products

Fifteen SRE PCR products, amplified using Phanta Max Super-Fidelity DNA Polymerase (Vazyme, P505), were purified with AMPure XP Beads (Beckman Coulter, A63881) and quantified using the Qubit® DNA Assay Kit and Qubit® 3.0 Fluorometer (Life Technologies, CA, USA). For library construction of each sample, in a clean 0.2ml PCR tube, 400 ng of sample, corresponding buffer, 0.75 μL Ultra II End-prep Enzyme Mix (NEB, E7546L), and 0.5 μL NEBNext FFPE DNA Repair Mix (NEB, M6630L) were added, and the volume was brought to 15 μL with nuclease-free water. After gentle mixing and a brief spin, the reaction was incubated at 20 °C for 5 minutes and then at 65 °C for 5 minutes to complete end repair. Each end-prep reaction was purified with 1× volume of AMPure XP Beads (AXP) and washed twice with 80% ethanol. For barcode ligation, the purified sample was mixed with 2.5 μL Native Barcode (NB01-24), 10 μL Blunt/TA Ligase Master Mix (NEB, M0367L) in a 0.2ml PCR tube. After gentle mixing and spinning down, the reaction was incubated at room temperature for 20 minutes and then terminated with 2 μL EDTA. All barcoded samples were mixed and purified with 0.4× volume of AMPure XP Beads (AXP) and washed twice with 80% ethanol. For adapter ligation, the pooled barcoded sample was mixed with 5 μL Native Adapter (NA), 10 μL NEBNext Quick Ligation Reaction Buffer (5X), and 5 μL Quick T4 DNA Ligase (NEB, E6056L) in a 1.5 mL Eppendorf LoBind tube. After gentle mixing and a brief spin, the reaction was incubated at room temperature for 20 minutes. The reaction was purified with 20 μL resuspended AMPure XP Beads and washed with 125 μL Long Fragment Buffer (LFB). Finally, the library was eluted with 12 μL Elution Buffer (EB) to obtain the sequencing-ready library. We loaded 50 fmol of the library onto R10.4.1 flow cells (FLO-MIN114) and sequencing on the GridION X5 platform following the manufacturer's instructions. Basecalling was performed in batches by ONT basecalling software version 7.1.4 with default parameters during sequencing.

Library preparation for Qitan Tech nanopore sequencing

Twenty-three SRE PCR products amplified using Phanta Max Super-Fidelity DNA Polymerase (Vazyme, P505) were purified with AMPure XP Beads (Beckman Coulter, A63881) and quantified using the Qubit® DNA Assay Kit and Qubit® 3.0 Fluorometer (Life Technologies, CA, USA). For end repair of the DNA, each 200 fmol sample was mixed with 2.5 μL of DNA repair buffer, 1 μL of DNA repair enzyme (DRM), 1 μL of end repair enzyme (EPM), and nuclease-free water to a final volume of 20 μL. The reaction mixture was incubated at 30 °C for 10 minutes, followed by 65 °C for 10 minutes. For barcode adapter ligation, the end-repaired DNA was combined with 2 μL of barcode, 7.5 μL of 4× ligation buffer, and 1.5 μL of DNA ligase, and the reaction was carried out at room temperature for 10 minutes. The reaction was then terminated with 2 μL of stop reaction buffer (TRB). All barcoded samples were pooled, purified with 0.4× volume of AMPure XP Beads, and washed twice with 80% ethanol. Subsequently, the sample was subjected to an additional purification step with 1× volume of AMPure XP Beads and eluted in 35 μL of nuclease-free water. For sequencing adapter ligation, in a 1.5 mL Eppendorf LoBind tube, 200 fmol of the purified product was mixed with 3.5 μL of sequencing adapter (SAC), 12.5 μL of 4× ligation buffer, 2 μL of DNA ligase (DLE), and nuclease-free water to a final volume of 50 μL. The reaction was performed at room temperature for 10 minutes, after which it was purified with 0.4× volume of AMPure XP Beads, washed with long fragment wash buffer (LWB), and finally eluted in 16 μL of elution buffer.

The final libraries were quantified via Qubit® 3.0 Fluorometer (Life Technologies, CA, USA) and loaded onto a QCell-384 V1.0 flow cell (Qitan Tech, Q-001-001-01). The sequencing was carried out using a QNome-3841 platform (Qitan Tech, China), strictly adhering to the manufacturer's protocols. Base calling was performed using QPreasy v3.2.1 with default parameters in real time during sequencing.

Long-read whole-genome sequencing data preprocessing

(1) FASTQ file filtering

We performed quality filtering on the raw FASTQ files from both tumor and blood samples using NanoFilt software (version 2.8.0), with parameters "-q 7 -l 1000". The filtering criteria retained reads with a sequencing quality score greater than 7 and a sequence length greater than 1000 bp. Through this process, we obtained clean FASTQ files, which contained sequence data that met the quality standards.

(2) Quality fontrol of flean FASTQ files

After the initial filtering of the clean FASTQ files, we further conducted quality control using NanoPlot software (version 1.39.0) with parameters "--maxlength 40000 --plots hex dot". During this process, we filtered out reads shorter than 40,000 bp and performed a detailed statistical analysis on these reads, including calculating key sequencing information such as the N50 value and total base count.

(3) Alignment to the reference genome

We used minimap2 (version 2.26) to align the clean FASTQ files from both tumor and blood samples to the hg38 reference genome from NCBI, generating SAM files with parameters "-MD -ax map-ont -L". Subsequently, we utilized SAMtools(version 1.9) to convert the SAM files into BAM files and sorted the BAM files to obtain sorted BAM files (sort BAM). Then, with the help of SAMtools(version 1.9) and awk commands, we added "tumor" and "blood" tags to each read in the sort BAM files for tumor and blood samples, respectively, ultimately generating sort tag BAM files.

Analysis and visualization of nanopore sequencing data for PCR products

After nanopore sequencing of the PCR products, we utilized the NanoFilt software (version 2.8.0) with parameters "-q 7 -l 4800" to filter reads that could fully span the repeat regions. Subsequently, we aligned the filtered reads to the hg38 reference genome from NCBI using minimap2 (version 2.26), generating SAM files with parameters "-MD -ax map-ont -L". Thereafter, we

converted the SAM files to BAM files using SAMtools(version 1.9). To avoid alignment errors, we excluded reads containing DEL longer than 50 bp through an in-house bioinformatics pipeline, considering that INS in such reads were likely due to alignment artifacts. Finally, we visualized the SRE-containing paired samples using the Integrative Genomics Viewer (IGV, version 2.16.0).

SomaGauss-SV workflow

(1) Merging BAM files and detecting structural variations

Using SAMtools(version 1.9), we merged the sorted tag BAM files of paired samples to generate a merged BAM file. Subsequently, we utilized Sniffles2 (version 2.0.6) to analyze the merged BAM file with parameters "-t 100 --minsupport 3 --mapq 50 --min-alignment-length 1000 --output-rnames --allow-overwrite --long-ins-length 100000 --cluster-binsize 500 --minsvlen 50". We filtered SVs based on the following criteria: read alignment quality greater than 50, supporting read count exceeding 3, SV length greater than 50 bp, and minimum alignment length greater than 1000 bp. The filtered SV information were then recorded in the merge.vcf file.

(2) Screening for candidate somatic structural variations

To accurately identify candidate somatic SVs, we implemented a multi-step screening strategy. First, an in-house script was used to preliminarily filter SVs reported by Sniffles2, retaining only those supported by reads tagged with "tumor", which were initially considered as candidate somatic SVs.

Based on the candidate somatic SVs, we further refined the screening process for DEL as follows:

- Region amplification and small BAM file generation: Using the start position of the candidate DEL as the center, we amplified a 500 bp region on both sides. Reads from these regions were extracted from the complete tumor and blood BAM files to generate corresponding small BAM files.

- DEL detection and preliminary screening: Using the pysam tool, we scanned each read in these small BAM files to find deletion events greater than 50 bp in length and similar to the DEL length reported by Sniffles2 (difference less than 20 bp). If no DEL meeting these criteria was found in the blood BAM, the candidate DEL was automatically determined as a genuine somatic DEL.
- Application of the mixed Gaussian distribution model: If DEL meeting the above conditions were found in the blood BAM, we input these DEL length data into the mixed Gaussian distribution model for analysis. We then distinguished DEL supported only by tumor reads (supporting reads greater than 3) and identified these as genuine somatic DEL.
- Overfitting issue resolution: To address potential overfitting in the mixed Gaussian model, we introduced an additional Student's *t*-test to compare the differences in DEL lengths between blood and tumor. Only when there was a significant difference in DEL lengths between blood and tumor, and the DEL was supported by with more than three tumor reads, was it ultimately identified as a genuine somatic DEL.

For candidate somatic INS/DUP, we employed the following screening strategy:

- Region amplification and re-detection: Using the start position of the candidate INS/DUP as the center, we amplified a 500 bp region on both sides to form a region list. INS variants in these regions were then re-detected using Straglr, a length-based Gaussian modeling approach with proven efficacy in repetitive regions, with parameters "--genotype_in_size --min_support 1 --max_str_len 50 --nprocs 200 --min_ins_size 50 --max_num_clusters 9".
- Preliminary screening: If an INS/DUP greater than 50 bp in length was also detected in the same region in the blood sample, and this INS was grouped with the INS in the tumor, the candidate INS/DUP was excluded. Otherwise, it was retained. After this step, the remaining candidate INS/DUP were identified as genuine somatic INS/DUP.

Through this rigorous screening process, we effectively reduce false positive caused by alignment errors and Sniffles2 clustering algorithms, thereby improving the accuracy and reliability of somatic SV identification.

(3) Normal population structural variation filtering

In the analysis of clinical sample pairs, we employed an additional screening step to further optimize somatic SV identification, which was not applied to cell line samples. Using Jasmine (version 1.1.5), we wrote the absolute paths of the VCF files obtained from the previous step, as well as the absolute paths of the structural variation VCF files of the Chinese normal population, as reported in previous literature, into a txt file (named "sample-VCF.txt") as input.cific steps are as follows:

- Generating the out_vcf file: Jasmine was used to integrate the two VCF files, generating a new VCF file (named "out_vcf") with parameters "threads=30 min_seq_id=0.9 k_jaccard=9 --dup_to_ins ignore_strand max_dist=500".
- Final screening and result determination: Further screening of the out_vcf file was performed using an in-house script. Specifically, SVs identical to those found in the Chinese normal population were removed, and the remaining variants were retained. These retained variations were ultimately identified as somatic structural variations in LSCC samples.

Through this series of steps, we can effectively distinguish somatic SVs in LSCC samples from common variations present in the normal population, thereby enhancing the accuracy and specificity of somatic SV identification.

(4) Manual screening for INV and BND

To enhance the precision of detecting INV and BND, we employed a visual analysis approach. Specifically, we utilized the Integrative Genomics Viewer (IGV) software to generate images of INV and BND detected across multiple samples. These images provide an intuitive display of the variations' positions in the genome, the distribution of reads involved, and the characteristics of the variations, facilitating detailed observation and comparison of these complex

variations.

Through manual screening, we meticulously analyzed these IGV images. During the screening process, we relied on the characteristics of the variations (such as the number of supporting reads, alignment quality, and boundaries of the variations) and their commonalities across different samples to select high-confidence INV and BND from the images. This process ensures the accuracy and reliability of the detection results, avoiding false positives due to algorithmic errors or data noise.

By combining visualization with manual screening, we can more accurately identify and validate INV and BND variations, thus providing higher-quality data support for subsequent biological analyses.

Detection of somatic structural variations with nanomonsv

According to the nanomonsv user manual, we processed the tumor BAM and blood BAM files obtained from the cell line's nanopore sequencing data, and set the parameters "--single_bnd --use_racon --threads 200 --control_panel_prefix" for analysis. Ultimately, we obtained the VCF file of somatic SVs detected by Nanomonsv.

Detection of somatic SVs with Severus

According to the Severus usage instructions, we processed the tumor BAM and blood BAM files obtained from the cell line's nanopore sequencing data and applied the parameters "-t 30 --output-read-ids --vntr-bed $vntr-bed" for analysis. This resulted in the VCF file of somatic SVs detected by Severus. The vntr file was sourced from https://github.com/KolmogorovLab/Severus/blob/main/vntrs/human_GRCh38_no_alt_analysis_set.trf.bed.

Detection of somatic SVs with SVision-pro

According to the SVision-prousage instructions, we processed the tumor BAM and blood BAM files obtained from the cell line's nanopore sequencing data and applied specific parameters "--preset error-prone --min_supp 3 --max_sv_size 1000000 --sample_name sampleid−−processnum60−−detectmodesomatic−−modelpathsampleid --process_num 60 --detect_mode somatic --model_path sampleid−−processn um60−−detectm odesomatic−−modelp athmodel_liteunet_1024_8_16_32_32_32.pth" for analysis. Ultimately, we obtained the VCF file of somatic SVs detected by Svision-pro. The model file was sourced from https://github.com/songbowang125/SVision-pro/tree/main/src/pre_process/model_liteunet_1024_8_16_32_32_32.pth.

Evaluating somatic SVs software

According to the method for SV assessment described in the reference literature, we chose to use Truvari as the assessment tool to compare the results of somatic SV detection with the gold standard. During the assessment process, we adopted the parameter settings "bench --pctseq 0" reported in the previous literature. Additionally, we employed minda as a complementary evaluation tool, using the parameters "truthset --tolerance 1000 --multimatch".

Correction and annotation of somatic INS consensus sequences

(1) Correction of consensus sequences

To enhance the accuracy of somatic INS sequences, we initially constructed new somatic INS VCF files based on the VCF list of authentic somatic INS. Subsequently, we employed Iris (version 1.0.4) to correct the consensus sequences provided by Sniffles2, with parameters set as "genome_buffer=1000 --keep_long_variants

threads=20". Iris optimizes the consensus sequences by integrating information from the genomic reference sequence, VCF files, and BAM files, thereby reducing sequence biases that may arise from sequencing or alignment errors.For sequence annotation and refinement, the consensus sequences of somatic insertions (INS) were first corrected using Iris and then annotated for repetitive elements using RepeatMasker. To characterize the genomic context of somatic deletions (DELs), their breakpoint regions were intersected with a RepeatMasker-annotated genome using BEDTools to identify overlaps with repetitive sequences. Detailed parameters for each tool are provided in the Supplemental Methods.

(2) Annotation of consensus sequences

Building upon the corrected consensus sequences, we further annotated them using RepeatMasker (version 4.1.2). RepeatMasker is a widely utilized tool capable of identifying and annotating repetitive sequences in the genome, such as transposons and satellite DNA. By annotating the corrected consensus sequences, we can better understand the origins and potential functional impacts of insertional variants. The parameters for RepeatMasker were set as "-species human -engine RMBlast -q -parallel 4".

Annotation of somatic DEL sequences

To further analyze the genomic context of somatic DEL, we employed BEDTools (version 2.30.0) to perform intersection analysis between the filtered somatic DEL breakpoints and the BED file of the hg38 reference genome annotated by RepeatMasker. This process aims to determine whether the deletion regions overlap with repetitive sequence regions in the genome and to obtain corresponding annotation information. The specific steps are as follows:

(1) Intersection analysis

Utilizing the intersect function of BEDTools, we conducted an intersection analysis between the breakpoints of somatic DEL and the BED file of the hg38

reference genome annotated by RepeatMasker. This step identifies the overlapping parts between the deletion regions and repetitive sequence regions.

(2) Annotation information merging

When a deletion region intersects with multiple annotated regions, to integrate these annotation informations, we used the "|" symbol to merge the annotation contents. For instance, if a deletion region overlaps with several different types of repetitive sequences (such as SINE elements and LINE1 elements), these annotation informations will be merged into "SINE|LINE1".

Statistical analysis of enrichment site regions

1) Data Acquisition: Retrieve BED files for gene elements and candidate *cis*-regulatory elements (CCRE) from the UCSC database.

2) Intersection Analysis: Employ BEDTools (version 2.30.0) to conduct an intersection analysis between the somatic SV loci of each sample and the aforementioned BED files. Summarize and tally the number of somatic SV loci within each element and the length of these elements.

3) Calculation of Enrichment Fold and Significance: Using the total count of somatic SV s and the genome's total length, compute the enrichment fold.

4) Chi-Square Test: With the aid of the fisher_exact package in Python, construct a contingency table based on the total number of somatic SVs, variations within elements, variations outside elements, and the genomic background. Perform a chi-square test to determine if there is a significant enrichment of sites in that region.

Sample enrichment analysis

Based on previous literature reports and the visualization of alignment data (Supplemental Fig. S1), we observed that alignment in repetitive regions, such as

simple repeat sequences, may lead to ambiguity. Despite using relevant software to correct for these areas, accurately calculating somatic SV loci within repetitive regions remains challenging. To address this issue, we created non-uniform window BED files specifically for sample enrichment statistics.

(1) Creation of non-uniform window BED files

The specific process is as follows:

- Extract simple repeat regions: Extract regions annotated as "Simple repeat" from the RepeatMasker annotation file of the reference genome.
- Segment the entire genome: Utilize these "Simple repeat" regions to segment the entire genome.
- Divide non-repetitive regions: Segment the genome, excluding "Simple repeat" regions, into windows of 500 bp/5 kb.
- Merge BED files: Combine the BED files of the "Simple repeat" regions with those of the evenly divided windows to obtain the final non-uniform window BED file.

(2) Enrichment statistics of somatic structural variations

- Intersection analysis: Use BEDTools (version 2.30.0) to perform intersection analysis between the somatic structural variations of each sample and the non-uniform window BED files created above.
- Summary statistics: Aggregate and count the samples and the number of samples present in each region to assess the enrichment of somatic structural variations across different regions.
- Merging of adjacent enriched regions: Because a single SV can span multiple predefined windows, we added a post-processing step to merge adjacent enriched regions. Specifically, if two regions that were shared by multiple samples were directly adjacent (0 bp apart), they were merged into a single continuous region prior to downstream analysis.

By employing this method, we can more accurately determine the distribution of somatic SVs in both repetitive and non-repetitive regions, providing more reliable data support for subsequent biological analyses.
