# Supplementary material for: Nanopore sequencing identifies high-frequency somatic structural variations in laryngeal squamous cell carcinoma genomes
Source: Genome Res. 2026 Nov;36(11):2307–21. doi: 10.1101/gr.281046.125 (PMC13739014; doi:10.1101/gr.281046.125)
Supplement: Supplementary file 2 [file Supplementary_Figures.pdf]

# Supplementary Figures for

## Nanopore Sequencing Unveils Somatic Structural Variations as Biomarkers in Laryngeal squamous cell carcinoma Genomes

Xuyan Liu et al.

*Corresponding author. Email: danxie@scu.edu.cn

**This PDF file includes:**

Supplemental Figures S1 – S23

## Contents

Supplemental Figure S1. IGV plot showing alignment errors in a simple tandem repeat region Chr6:515,243-516,068. .......... 2
Supplemental Figure S2. Somatic SV detection softwares evaluation: distinguishing SV types. .......... 3
Supplemental Figure S3. Relationship between somatic deletions and other pathological information. .......... 5
Supplemental Figure S4. Overview of SV feature distribution. .......... 7
Supplemental Figure S5. Oncoplot of high-frequency INS identified bySeverus and SomaGauss-SV. .......... 9
Supplemental Figure S6. Overview of sample enrichment regions and related genes. .......... 10
Supplemental Figure S7. Overview of INV hotspot (Chr7:77,175,000 – 102,599,637). .......... 12
Supplemental Figure S8. Overview of SRE details. .......... 13
Supplemental Figure S9. Somatic SREs IGV plots of sample LSCC 16, 17 and 18. .......... 15
Supplemental Figure S10.Somatic SREs IGV plots of sample LSCC 19. .......... 17
Supplemental Figure S11. Validation of SRE authenticity using additional clinical samples. .......... 19
Supplemental Figure S12. IGV plots of Samples LSCC 21 and 22. .......... 21
Supplemental Figure S13. IGV plots of Samples LSCC 23 and 25. .......... 23
Supplemental Figure S14. IGV plots of Samples LSCC 23 and 25. .......... 26
Supplemental Figure S15. IGV plots of Samples LSCC 28. .......... 27
Supplemental Figure S16. IGV plots of Samples LSCC 29 and 36. .......... 28
Supplemental Figure S17. IGV plots of Samples LSCC 37 and 39. .......... 30
Supplemental Figure S18. IGV plots of Samples LSCC 40 and 41. .......... 32
Supplemental Figure S19. IGV plots of Samples LSCC 42. .......... 35
Supplemental Figure S20. Functional investigation of SRE in cell lines. .......... 36
Supplemental Figure S21. Summary statistics of complex SVs. .......... 39
Supplemental Figure S22. Complex SV (INV+DUP) in LSCC3. .......... 40
Supplemental Figure S23. Benchmarking and staged evaluation of somatic SV detection workflows. .......... 41

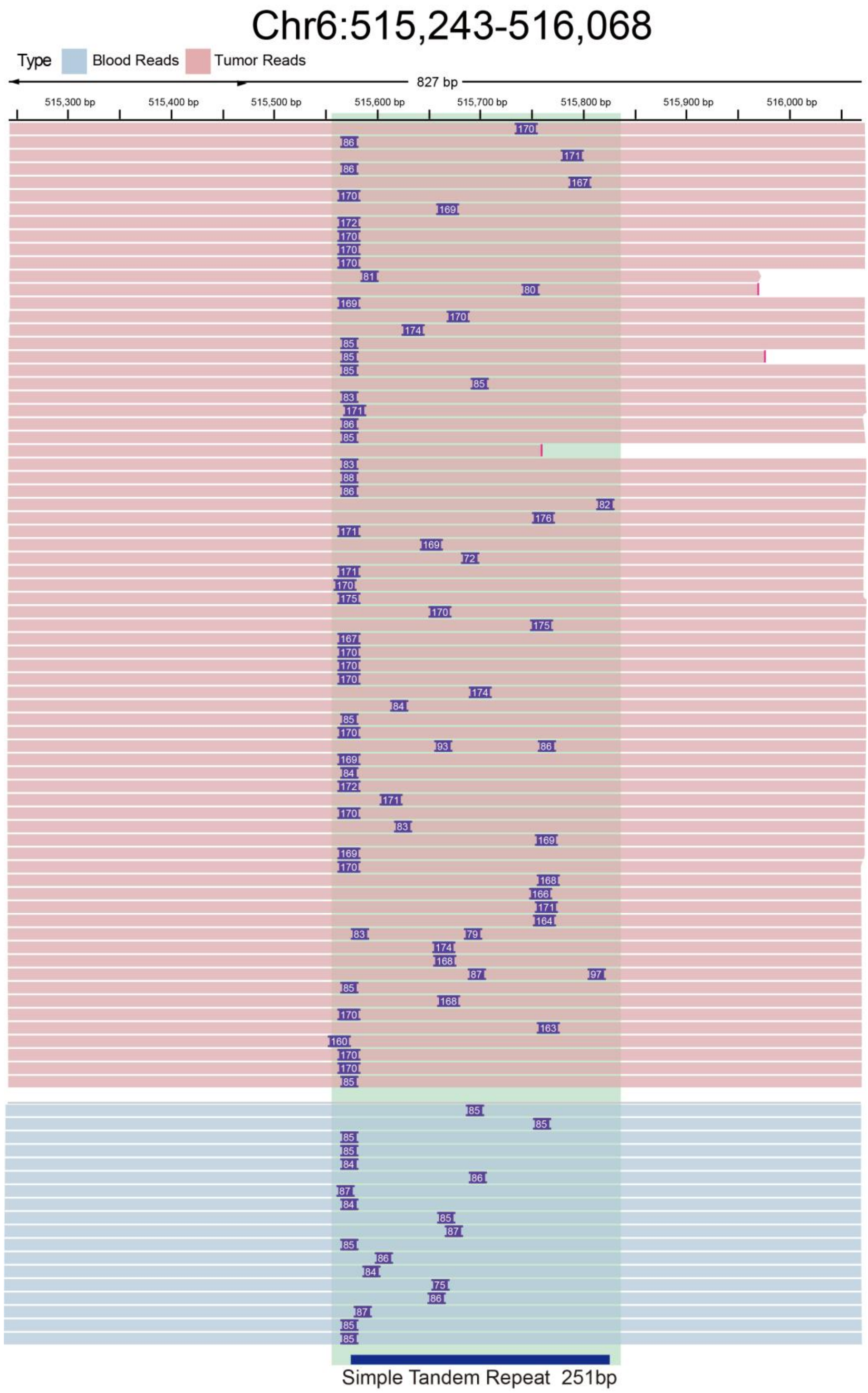


**Supplemental Figure S1. IGV plot showing alignment errors in a simple tandem repeat region Chr6:515,243-516,068.**

Insertions are scattered within the repeat region, making it difficult to determine whether they are somatic insertions. Pink fragments represent tumor sample reads, light blue fragments represent reads from the paired normal sample, and dark blue fragments highlight the repeat region.

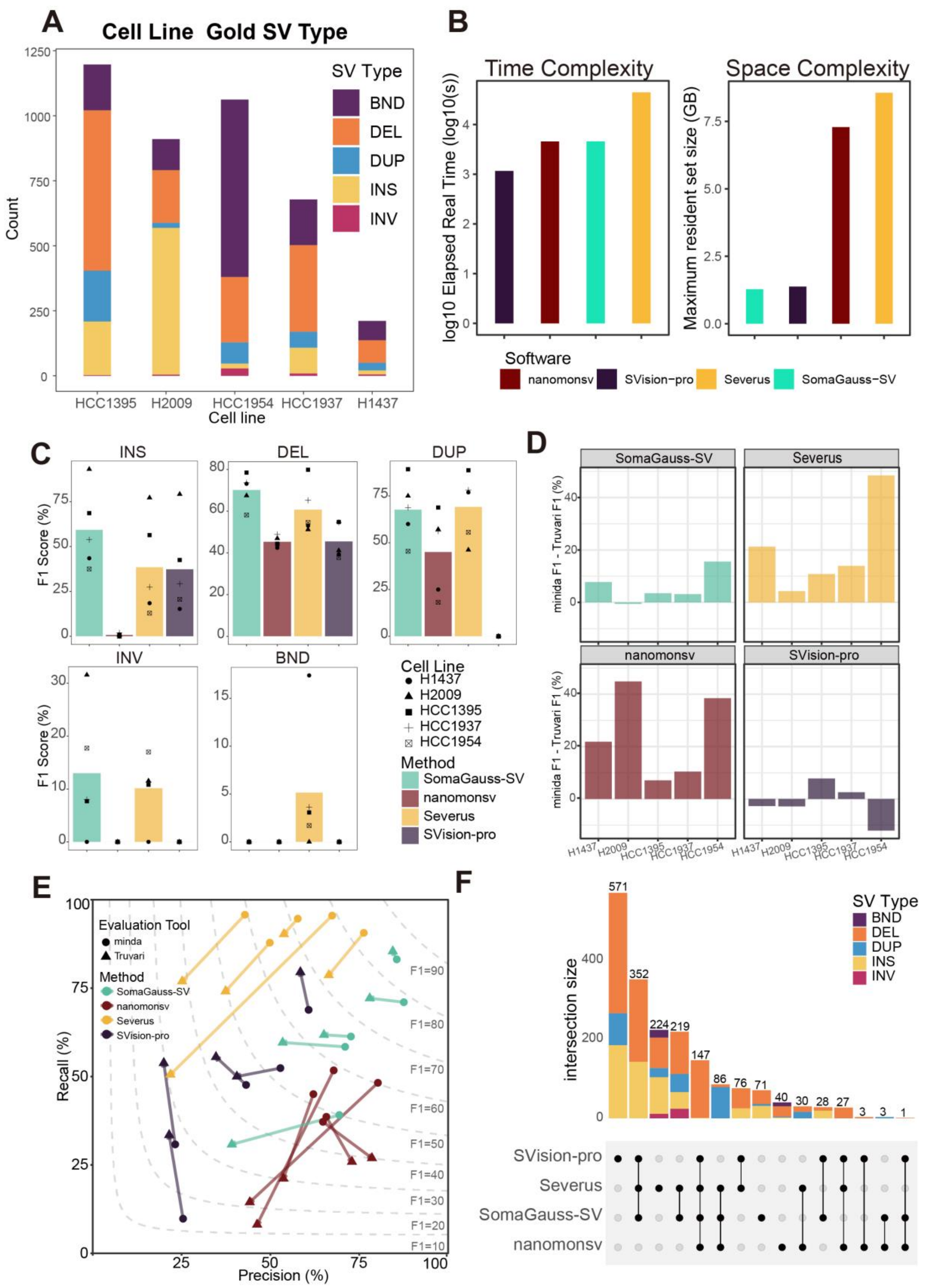


**Supplemental Figure S2. Somatic SV detection softwares evaluation: distinguishing SV types.**

**(A).**Stacked bar chart of gold-standard SV types for different cell lines, with colors indicating different SV types.**(B)**.Bar plots of software space complexity (left) and time complexity (right).**(C).**Bar chart of F1 scores for software distinguishing SV types. The height of the bars represents the average F1 score for each software, while the points indicate the F1 scores for

individual cell lines. **(D).**Bar plot of F1 score differences across different evaluation strategies.**(E)**.Contour plot of different assessment strategies in software. The X-axis represents precision, the Y-axis represents recall, the contours in the plot represent F1 scores, and software and cell lines are distinguished by the color and shape of the points, respectively.Bar plot of F1 score differences across different evaluation strategies.**(F).**UPset plot of SV proportions for different software in the HCC1395 cell line, while colors representing different SV types. Black circles below represent intersections between software.

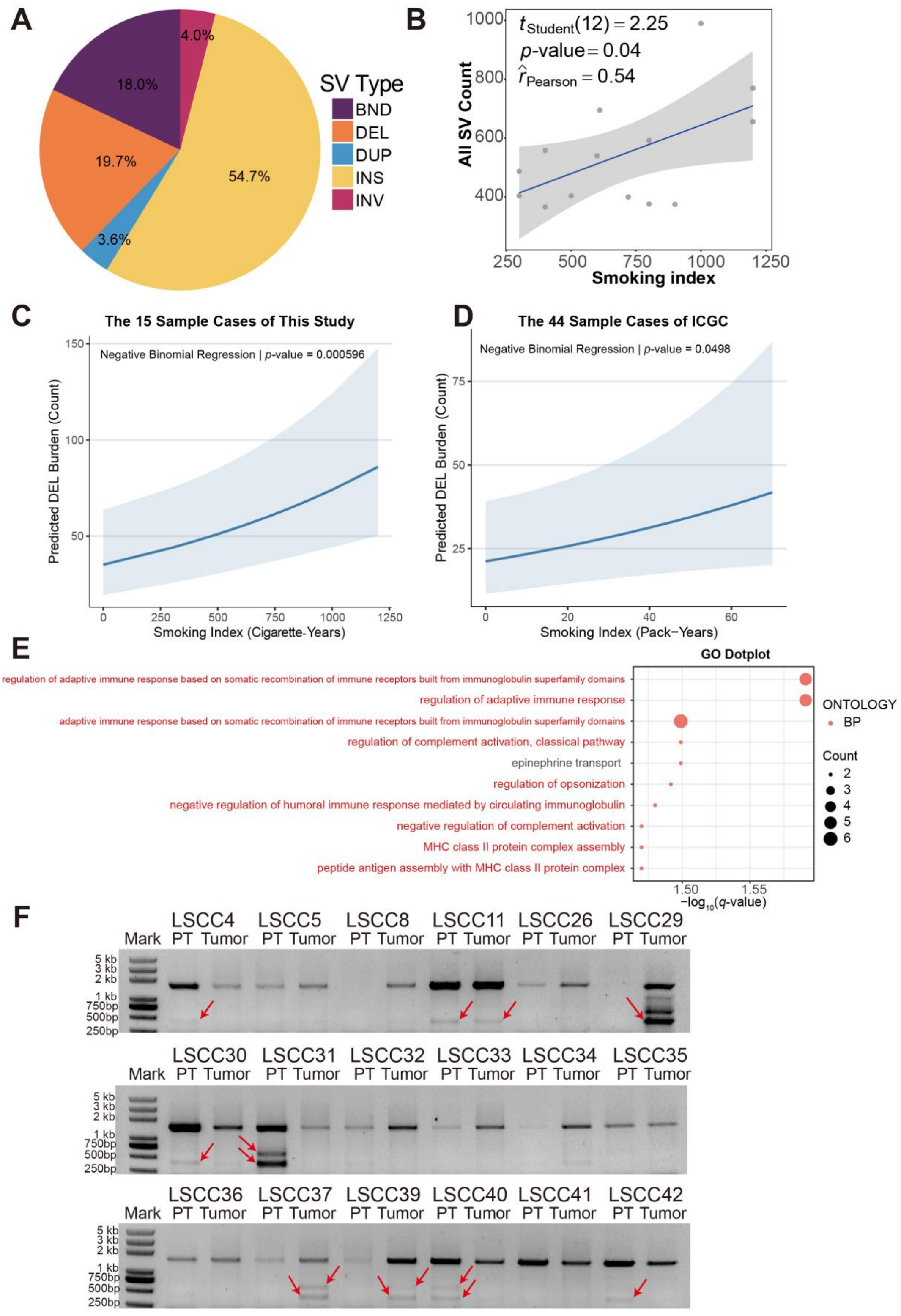


**Supplemental Figure S3. Relationship between somatic deletions and other pathological information.**

**(A).**Pie chart of the proportion of overall somatic structural variations. **(B).**Correlation plot of total somatic SVs and smoking index. The X-axis represents the smoking index, and the Y-axis represents the total number of somatic structural variations, with degrees of freedom, *p*-value, and Pearson correlation coefficient marked on the plot. **(C).**Negative binomial

regression plot validating the association between smoking index and deletion burden in 15 samples from this study, *p*-value marked on the plot. **(D).**Negative binomial regression plot validating the association between smoking index and deletion burden in 44 samples from ICGC, *p*-value marked on the plot. **(E).** GO enrichment plot for genes affected by common DELs in multiple samples from smokers. The X-axis represents $-\log_{10}(q\text{-value})$, and the Y-axis represents the names of enriched biological processes, with immune-related processes highlighted in red. Circle size indicates the number of enriched genes($q$-value $< 0.05$).**(F).**Gel electrophoresis of MUC3A cDNA; red arrows indicate fragments containing deletions. The expected full-length MUC3A PCR product is 1469 bp. The shorter fragments (346–349 bp; indicated by red arrows) correspond to transcripts carrying the somatic deletion.

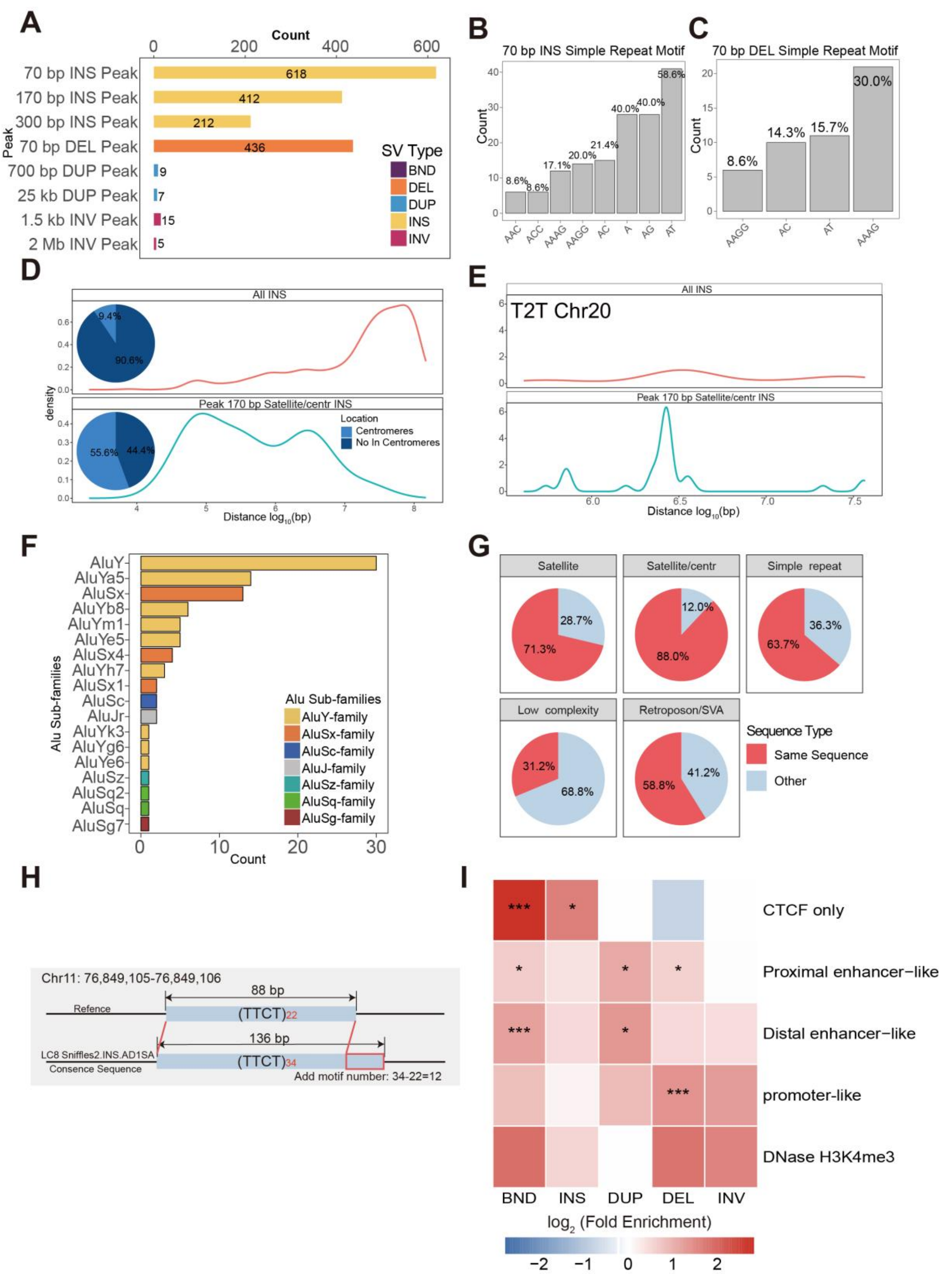


**Supplemental Figure S4. Overview of SV feature distribution.**

**(A).**Bar chart of SV counts at length peaks. The X-axis represents the count, and the Y-axis labels the type and peak. **(B).**Bar chart of INS motif counts annotated as Simple repeat at 70 bp. The X-axis labels the normalized motif types, and the Y-axis represents the number of INS. **(C).**Bar chart of DEL motif counts annotated as Simple repeat at 70 bp. The X-axis labels the normalized motif types, and the Y-axis represents the number of DEL. **(D).**Distribution plot of overall INS and INS annotated as Satellite/centr at 170 bp with respect to the centromeric region. The pie chart indicates the proportion of INS located in the centromeric region. Light blue represents INS within the centromeric region, and dark blue represents INS outside the

centromeric region. The distribution of distances from the centromere for INS outside the centromeric region is shown in the lower plot. **(E).**Density distribution of distance to centromere for overall insertions and Satellite/centromeric insertions at the 170 bp peak using T2T Chr20 reference genome.**(F).**Bar chart of INS *Alu* subfamily counts annotated as SINE/*Alu* at 300 bp. The X-axis represents the number of INS, and the Y-axis represents the names of *Alu*. Colors indicate the *Alu* subfamilies. **(G).**Pie chart of sequence composition of INS in repeat regions. Red indicates INS containing the same repeat sequence, and blue indicates other sequences. **(H).**Schematic of somatic insertions in Simple repeat regions. The top shows the reference genome repeat motif and motif number, and the bottom shows the repeat motif and motif number of the somatic INS consensus sequence in the tumor sample. **(I).**Heatmap of regulatory element enrichment in gene-proximal regions. Each row represents a different SV type, and each column represents different regulatory elements and their ±300 bp range, with color intensity indicating $\log_2$(Fold Enrichment) (Fisher's exact test, **p*-value < 0.05, ***p*-value < 0.01, ****p*-value < 0.001).

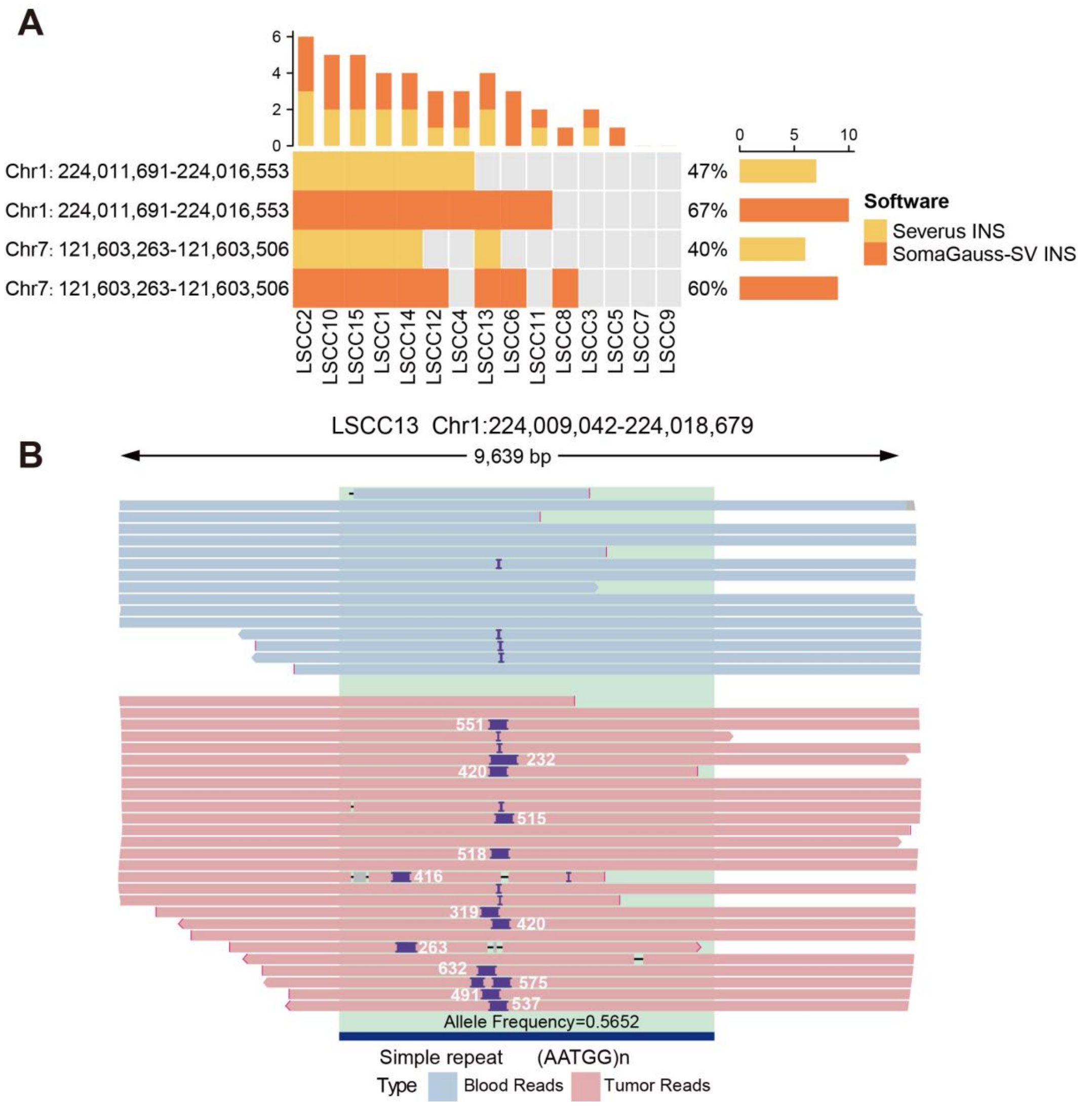


**Supplemental Figure S5. Oncoplot of high-frequency INS identified bySeverus and SomaGauss-SV.**

**(A).** Sample distribution of high-frequency INS (shared by more than five samples) concordantly identified by both Severus and SomaGauss-SV. Yellow indicates samples detected by Severus, and orange indicates samples detected by SomaGauss-SV. For INS detected exclusively by SomaGauss-SV, manual validation was performed using IGV. **(B).**Representative IGV validation of an INS exclusively detected by SomaGauss-SV, exemplified by the locus Chr1:224,009,042–224,018,679 in sample LSCC13. As shown in (A), this INS was not reported by Severus in LSCC13.

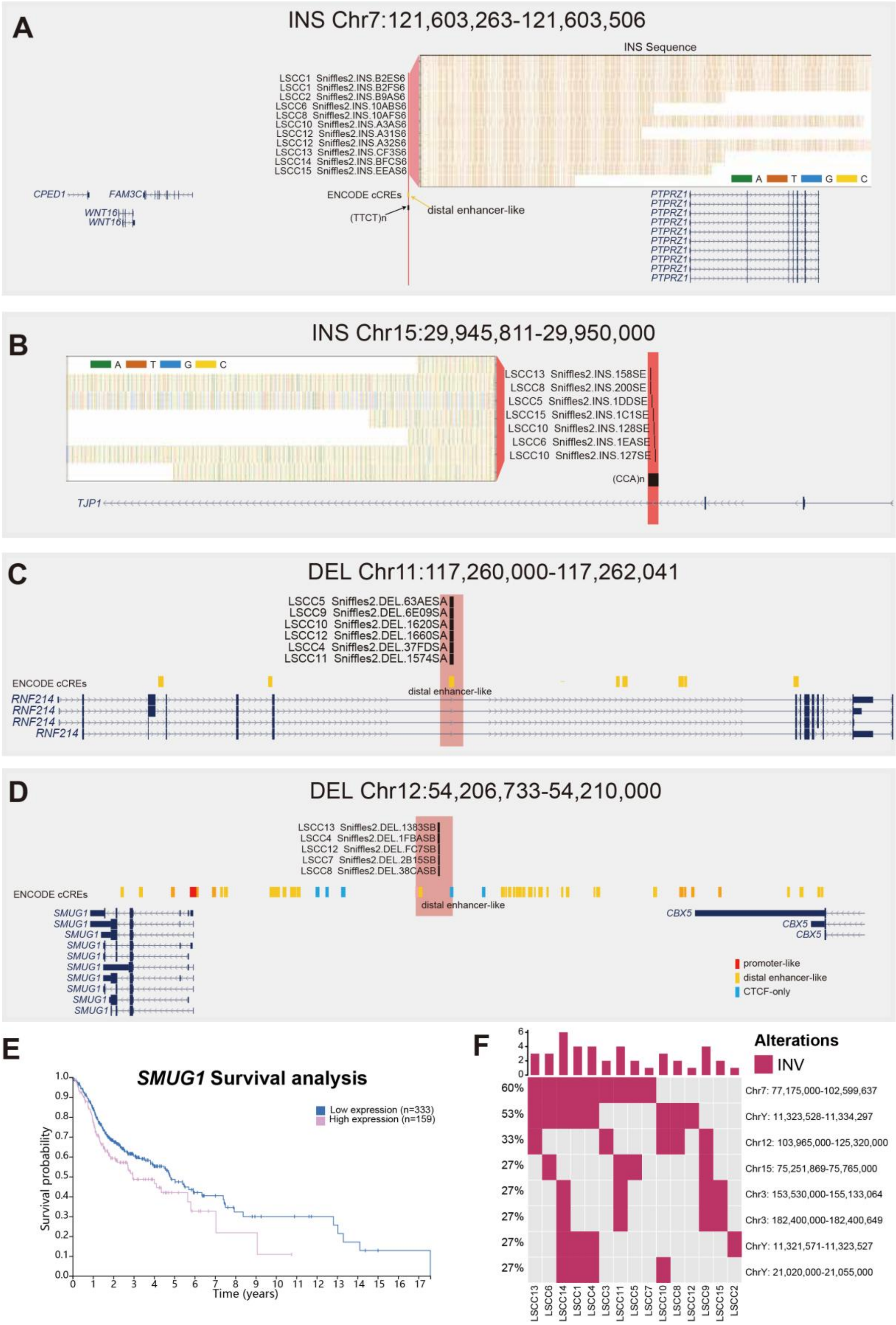


**Supplemental Figure S6. Overview of sample enrichment regions and related genes.**

**(A).**Browser plot of somatic INS (Chr7:121,603,263-121,603,506) illustrating the following epigenomic signals: a stacked bar chart of INS positions and inserted sequences, with different colors representing different bases; Candidate *cis*-regulatory Elements (cCREs) predicted by ENCODE and ENCODE-annotated gene transcripts. The plot concludes with the TTCT motif of the repeat. **(B).**Browser plot of somatic INS (Chr15:29,945,811-29,950,000) illustrating the

following epigenomic signals: a stacked bar chart of INS positions and inserted sequences, with different colors representing different bases. The plot concludes with the CCA motif of the repeat and the ENCODE-annotated gene transcript. **(C).**Browser plot of somatic DEL (Chr11:117,260,000-117,260,041) illustrating the following epigenomic signals: DEL positions, Candidate *cis*-regulatory Elements (cCREs) predicted by ENCODE, and ENCODE-annotated gene transcripts. **(D).**Browser plot of somatic DEL (Chr12:54,206,733-54,210,000) illustrating the following epigenomic signals: DEL positions, Candidate *cis*-regulatory Elements (cCREs) predicted by ENCODE, and ENCODE-annotated gene transcripts. **(E).**Kaplan-Meier survival curve for the SMUG1 gene. Blue represents low expression of the SMUG1 gene, and pink represents high expression. **(F).**Waterfall plot of INV sample enrichment regions. Rows represent samples, and columns represent regions.

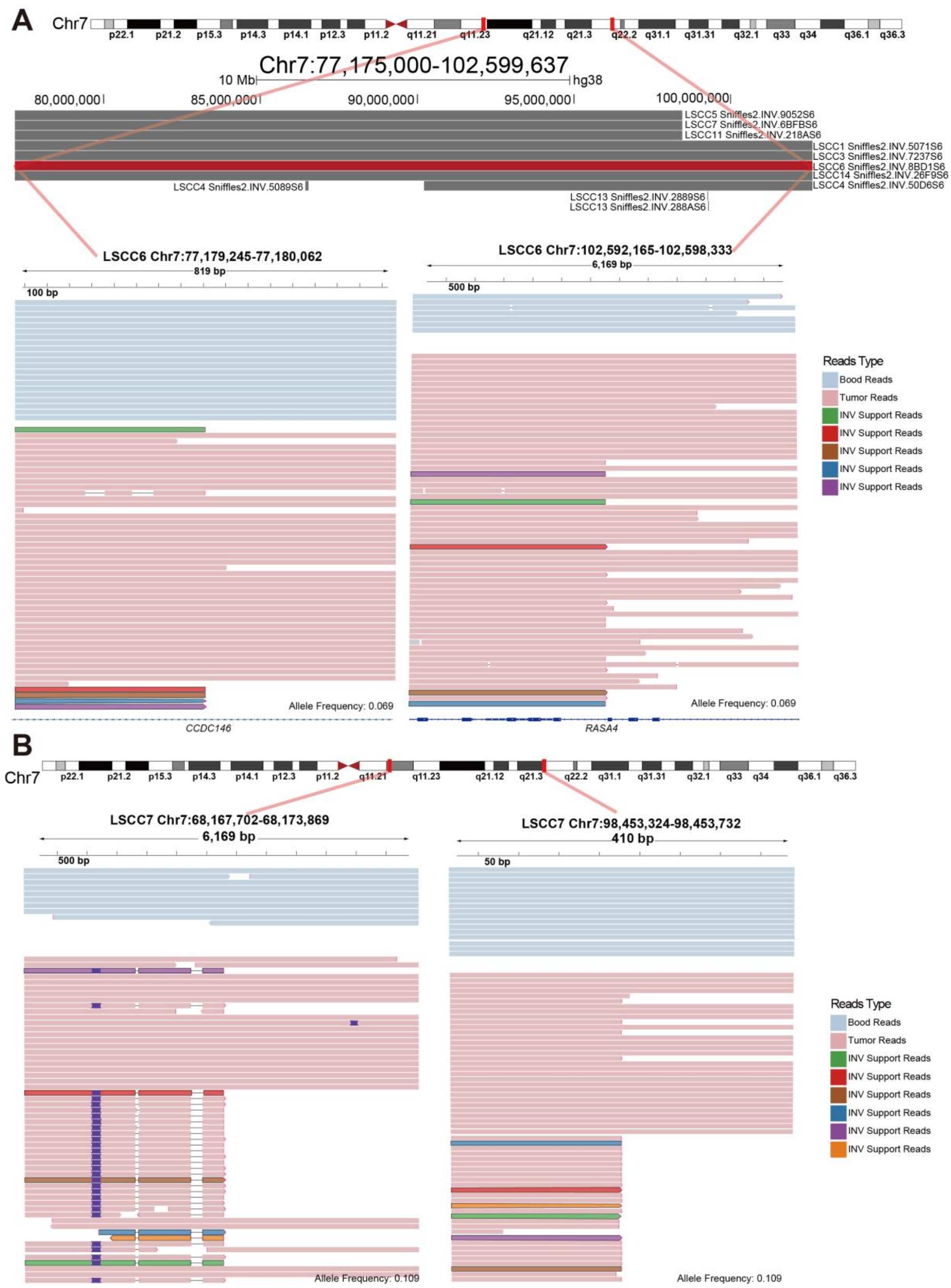


**Supplemental Figure S7. Overview of INV hotspot (Chr7:77,175,000 – 102,599,637).**

**(A).** Genome browser view of the INV region. Top: position of the INV on Chromosome 7. Middle: INV coverage across samples. Bottom: IGV alignments flanking the INV in LSCC6; supporting reads are color-coded. **(B).** IGV plot of the INV hotspot in LSCC7 (Chr7:77,175,000–102,599,637). Top: position of the INV on Chromosome 7. Bottom: IGV alignments flanking the INV in LSCC7; supporting reads are color-coded.

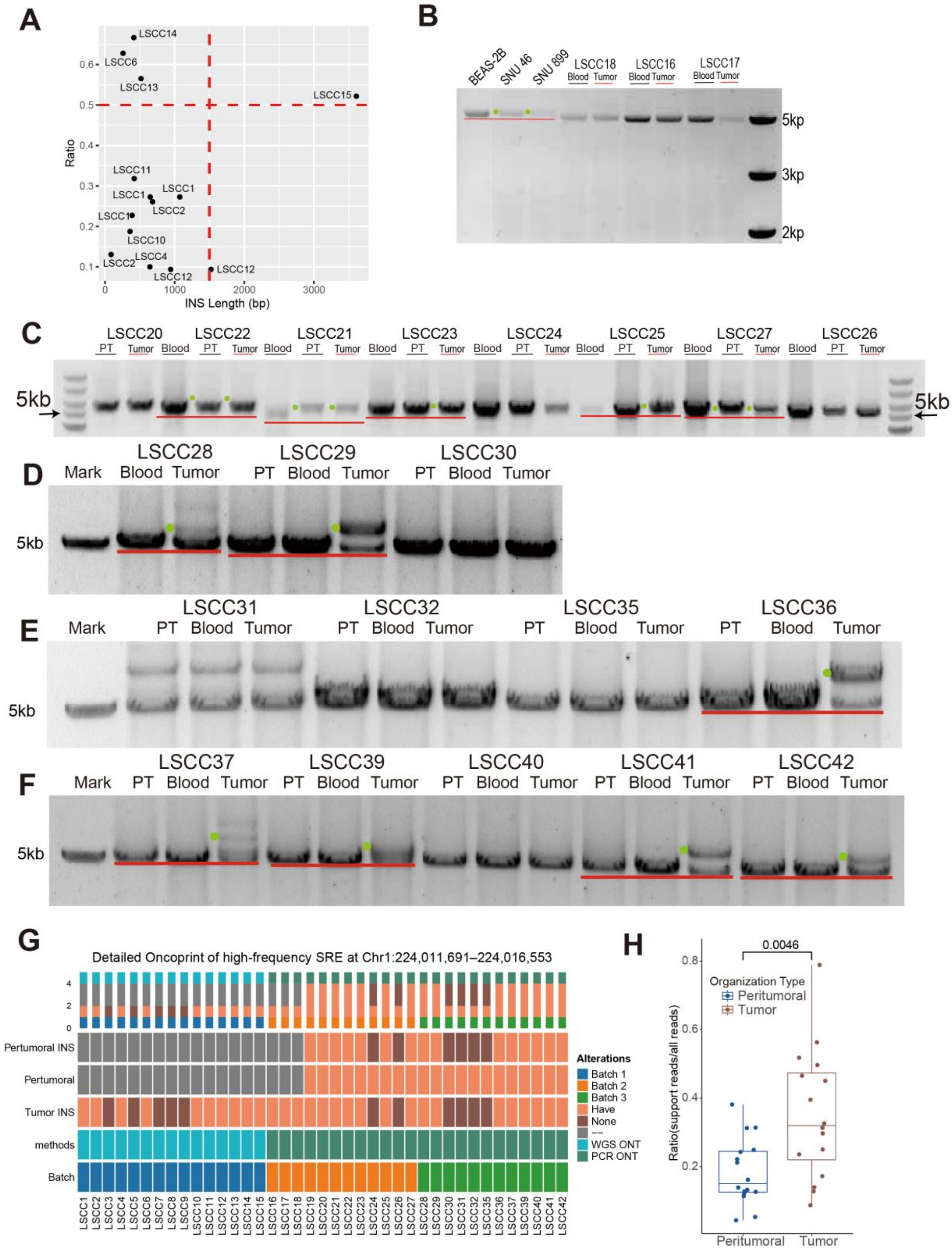


**Supplemental Figure S8. Overview of SRE details.**

**(A).**Scatter plot of somatic SRE length and ratio distribution. The X-axis represents SRE length, and the Y-axis represents SRE ratio. The red dashed line parallel to the X-axis indicates a Ratio of 0.5, and the red dashed line parallel to the Y-axis indicates an SRE length of 1500 bp. **(B).**Horizontal gel electrophoresis images showing DNA fragments from blood and tumor tissues of LSCC samples and cell lines. **(C-F).**Horizontal gel electrophoresis images showing DNA fragments from blood and tumor tissues of LSCC samples and cell lines. **(G).** Sample validation waterfall plot of high-frequency somatic SRE (Chr1: 224,011,691–224,016,553). Batch indicates the collection batch: Batch1 represents the 15 samples from ONT whole-genome sequencing, Batch2 represents the 12 samples from the second collection for

SRE validation, and Batch3 represents the 12 samples collected for this revision; Methods indicate the SRE validation methods (WGS ONT and PCR ONT); Tumor INS indicates the presence of this SRE in the tumor sample; Pertumoral indicates whether paired adjacent pertumoral tissue samples were collected (gray indicates not collected, orange indicates collected). Pertumoral INS indicates the presence of SRE in the pertumoral tissue sample, with None (brown) indicating absence and Have (orange) indicating presence.**(H).**Box plot of allele frequencies in tumor and peritumoral tissues (*t*-test).

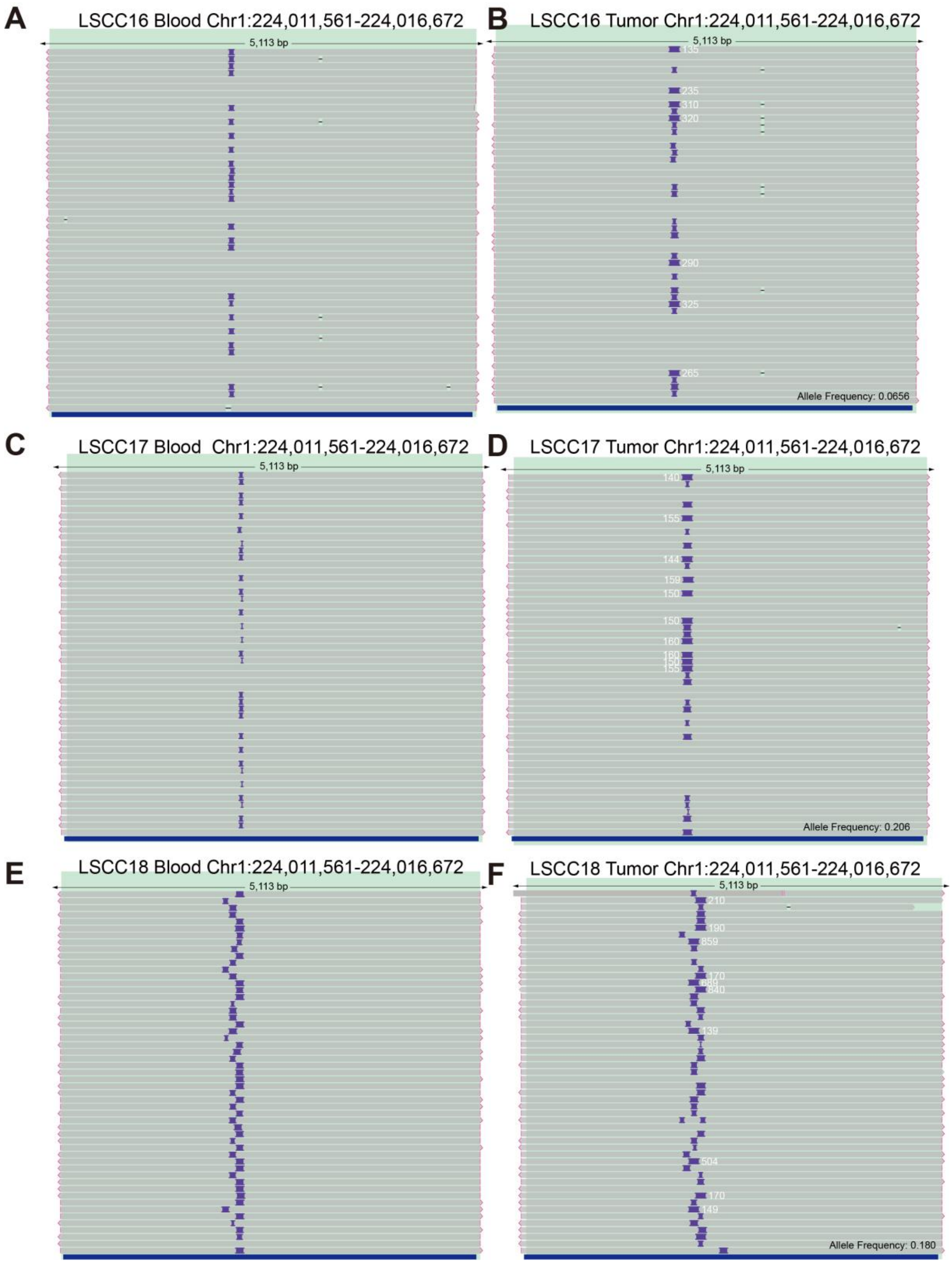


**Supplemental Figure S9. Somatic SREs IGV plots of sample LSCC 16, 17 and 18.**

**(A).**IGV plot of LSCC 16 blood (Chr1:224,009,042-224,018,679). Dark blue indicates the Simple repeat region with the AATGG motif. **(B).**IGV plot of LSCC 16 tumor (Chr1:224,009,042-224,018,679). Dark blue indicates the Simple repeat region with the AATGG motif. White numbers in the plot mark insertions greater than 70 bp.**(C).**IGV plot of LSCC 17 blood (Chr1:224,009,042-224,018,679). Dark blue indicates the Simple repeat region with the AATGG motif. **(D).**IGV plot of LSCC 17 tumor (Chr1:224,009,042-224,018,679). Dark blue indicates the Simple repeat region with the AATGG motif. White numbers in the plot mark insertions greater than 70 bp.**(E).**IGV plot of

LSCC 18 blood (Chr1:224,009,042-224,018,679). Dark blue indicates the Simple repeat region with the AATGG motif. **(F).**IGV plot of LSCC 18 tumor (Chr1:224,009,042-224,018,679). Dark blue indicates the Simple repeat region with the AATGG motif. White numbers in the plot mark insertions greater than 70 bp.

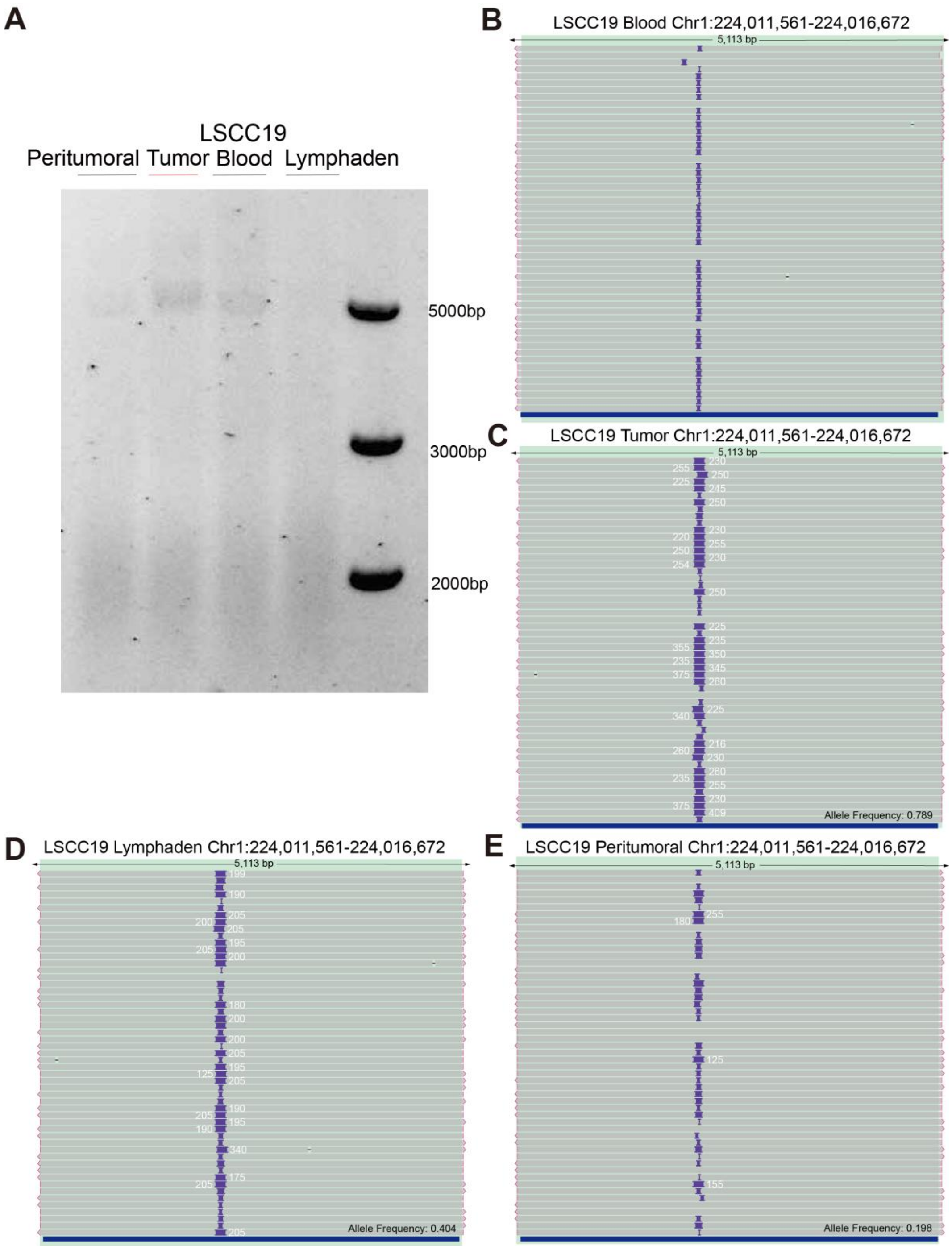


**Supplemental Figure S10.Somatic SREs IGV plots of sample LSCC 19.**

**(A).**Horizontal gel electrophoresis images showing DNA fragments from blood, tumor, peritumoral, and lymphaden tissues of LSCC 19 samples. **(B).**IGV plot of LSCC 19 blood (Chr1:224,009,042-224,018,679). Dark blue indicates the Simple repeat region with the AATGG motif. **(C).**IGV plot of LSCC 19 tumor (Chr1:224,009,042-224,018,679). Dark blue indicates the Simple repeat region with the AATGG motif. White numbers in the plot mark insertions greater than 70 bp. **(D).**IGV plot of LSCC 19 lymphaden (Chr1:224,009,042-224,018,679). Dark blue indicates the Simple repeat region with the AATGG motif. White numbers in the plot mark insertions greater than 70 bp. **(E).**IGV plot

of LSCC 19 peritumoral (Chr1:224,009,042-224,018,679). Dark blue indicates the Simple repeat region with the AATGG motif. White numbers in the plot mark insertions greater than 70 bp.

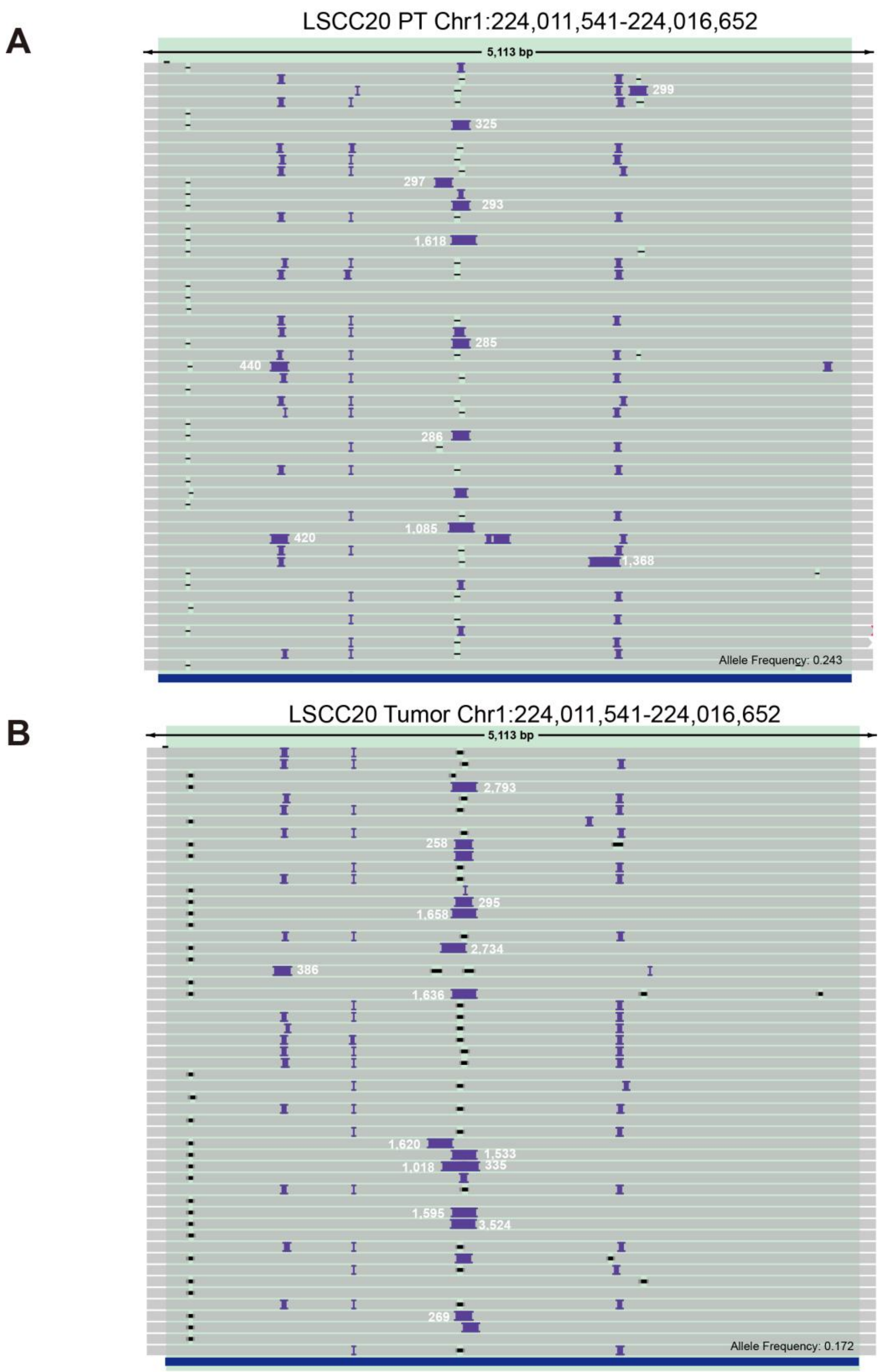


**Supplemental Figure S11. Validation of SRE authenticity using additional clinical samples.**

**(A).**IGV plot of LSCC 20 peritumoral (Chr1:224,009,042-224,018,679). Dark blue indicates the Simple repeat region with the AATGG motif. White numbers in the plot mark insertions greater than 70 bp. **(B).**IGV plot of LSCC 20 tumor (Chr1:224,009,042-224,018,679). Dark blue indicates the Simple repeat region with the AATGG motif. White numbers in the plot mark insertions greater than 70 bp.

196

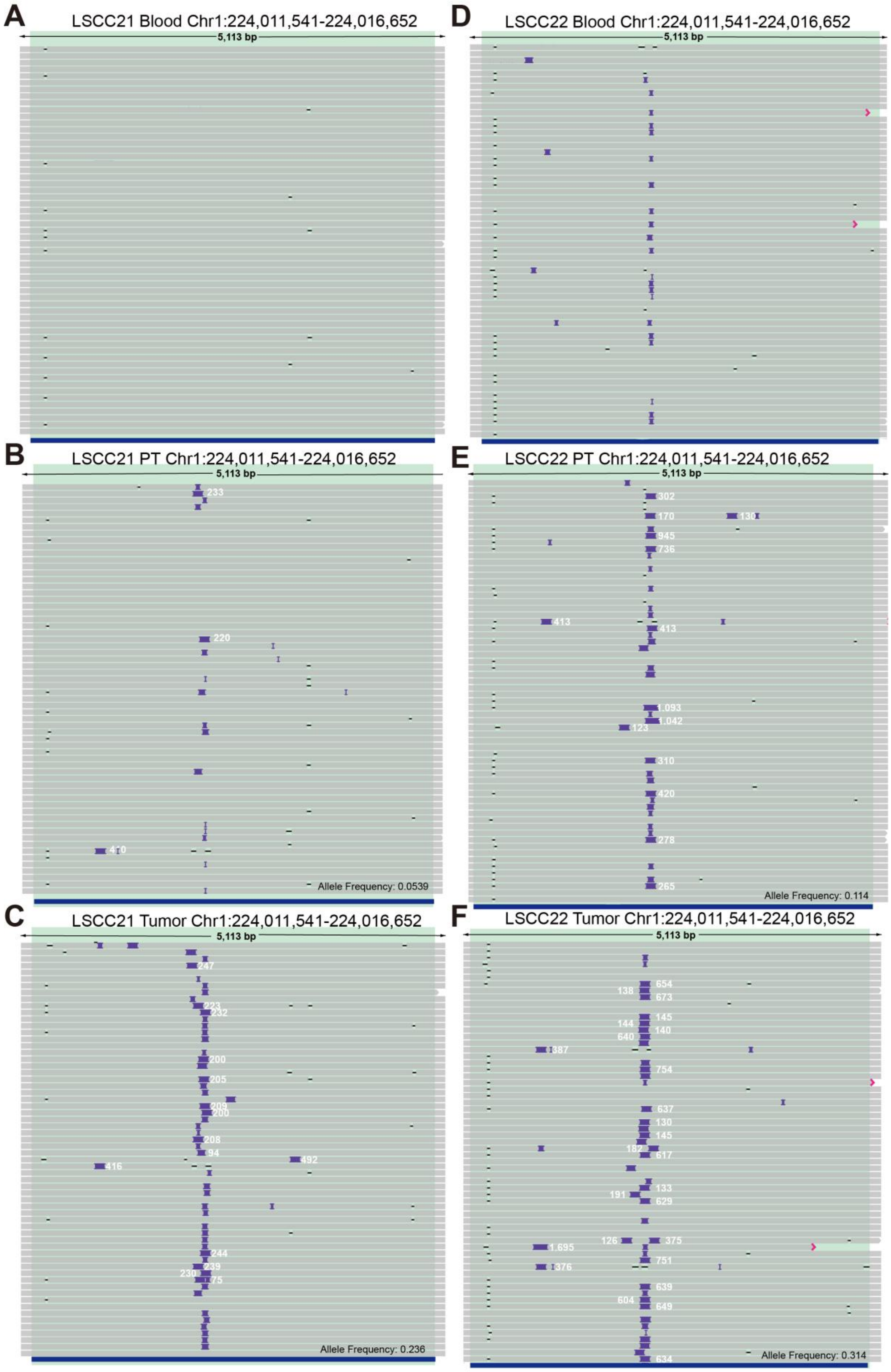


**Supplemental Figure S12. IGV plots of Samples LSCC 21 and 22.**

**(A).**IGV plot of LSCC 21 blood (Chr1:224,009,042-224,018,679). Dark blue indicates the Simple repeat region with the AATGG motif. **(B).**IGV plot of LSCC 21 peritumoral (Chr1:224,009,042-224,018,679). Dark blue indicates the Simple repeat region with the

AATGG motif. White numbers in the plot mark insertions greater than 70 bp. **(C).**IGV plot of LSCC 21 tumor (Chr1:224,009,042-224,018,679). Dark blue indicates the Simple repeat region with the AATGG motif. White numbers in the plot mark insertions greater than 70 bp. **(D).**IGV plot of LSCC 22 blood (Chr1:224,009,042-224,018,679). Dark blue indicates the Simple repeat region with the AATGG motif.**(E).**IGV plot of LSCC 22 peritumoral (Chr1:224,009,042-224,018,679). Dark blue indicates the Simple repeat region with the AATGG motif. White numbers in the plot mark insertions greater than 70 bp.**(F).**IGV plot of LSCC 22 tumor (Chr1:224,009,042-224,018,679). Dark blue indicates the Simple repeat region with the AATGG motif. White numbers in the plot mark insertions greater than 70 bp.

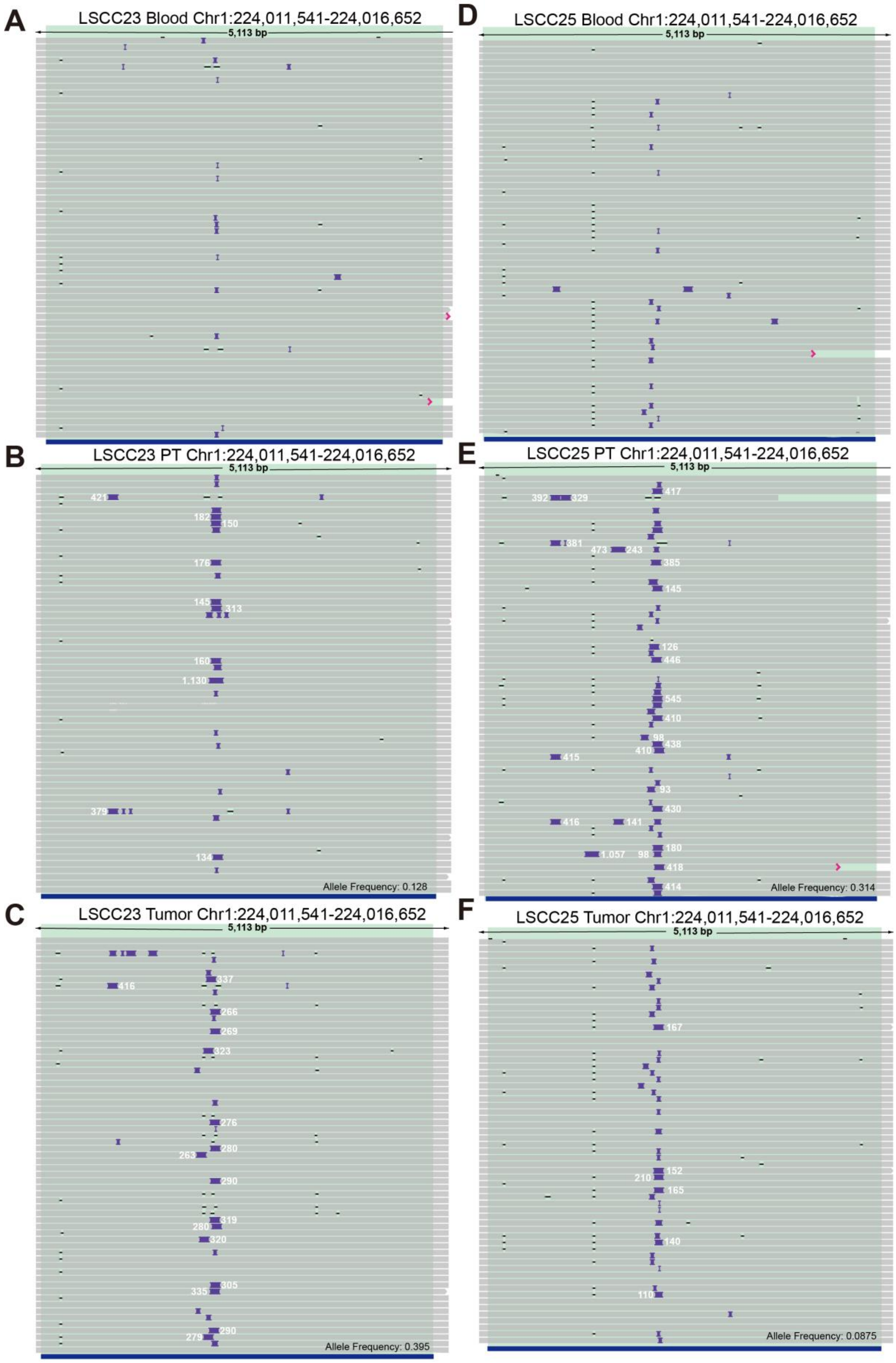


**Supplemental Figure S13. IGV plots of Samples LSCC 23 and 25.**

**(A).**IGV plot of LSCC 23 blood (Chr1:224,009,042-224,018,679). Dark blue indicates the Simple repeat region with the AATGG motif. **(B).**IGV plot of LSCC 23 peritumoral

(Chr1:224,009,042-224,018,679). Dark blue indicates the Simple repeat region with the AATGG motif. White numbers in the plot mark insertions greater than 70 bp.**(C).**IGV plot of LSCC 23 tumor (Chr1:224,009,042-224,018,679). Dark blue indicates the Simple repeat region with the AATGG motif. White numbers in the plot mark insertions greater than 70 bp. **(D).**IGV plot of LSCC 25 blood (Chr1:224,009,042-224,018,679). Dark blue indicates the Simple repeat region with the AATGG motif. **(E).**IGV plot of LSCC 25 peritumoral (Chr1:224,009,042-224,018,679). Dark blue indicates the Simple repeat region with the AATGG motif. White numbers in the plot mark insertions greater than 70 bp. **(F).**IGV plot of LSCC 25 tumor (Chr1:224,009,042-224,018,679). Dark blue indicates the Simple repeat region with the AATGG motif. White numbers in the plot mark insertions greater than 70 bp.

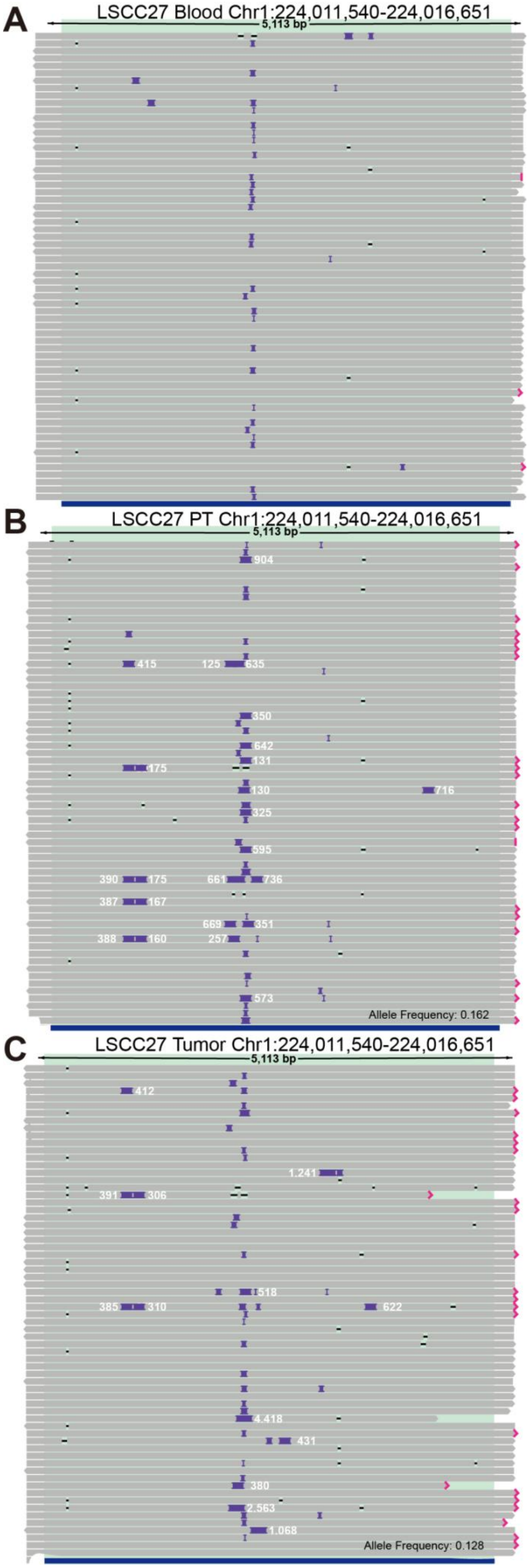
A
LSCC27 Blood Chr1:224,011,540-224,016,651
5,113 bp
B
LSCC27 PT Chr1:224,011,540-224,016,651
5,113 bp
Allele Frequency: 0.162
C
LSCC27 Tumor Chr1:224,011,540-224,016,651
5,113 bp
Allele Frequency: 0.128

**Supplemental Figure S14. IGV plots of Samples LSCC 23 and 25.**

**(A).**IGV plot of LSCC 27 blood (Chr1:224,009,042-224,018,679). Dark blue indicates the Simple repeat region with the AATGG motif. **(B).**IGV plot of LSCC 27 peritumoral (Chr1:224,009,042-224,018,679). Dark blue indicates the Simple repeat region with the AATGG motif. White numbers in the plot mark insertions greater than 70 bp.**(C).**IGV plot of LSCC 27 tumor (Chr1:224,009,042-224,018,679). Dark blue indicates the Simple repeat region with the AATGG motif. White numbers in the plot mark insertions greater than 70 bp.

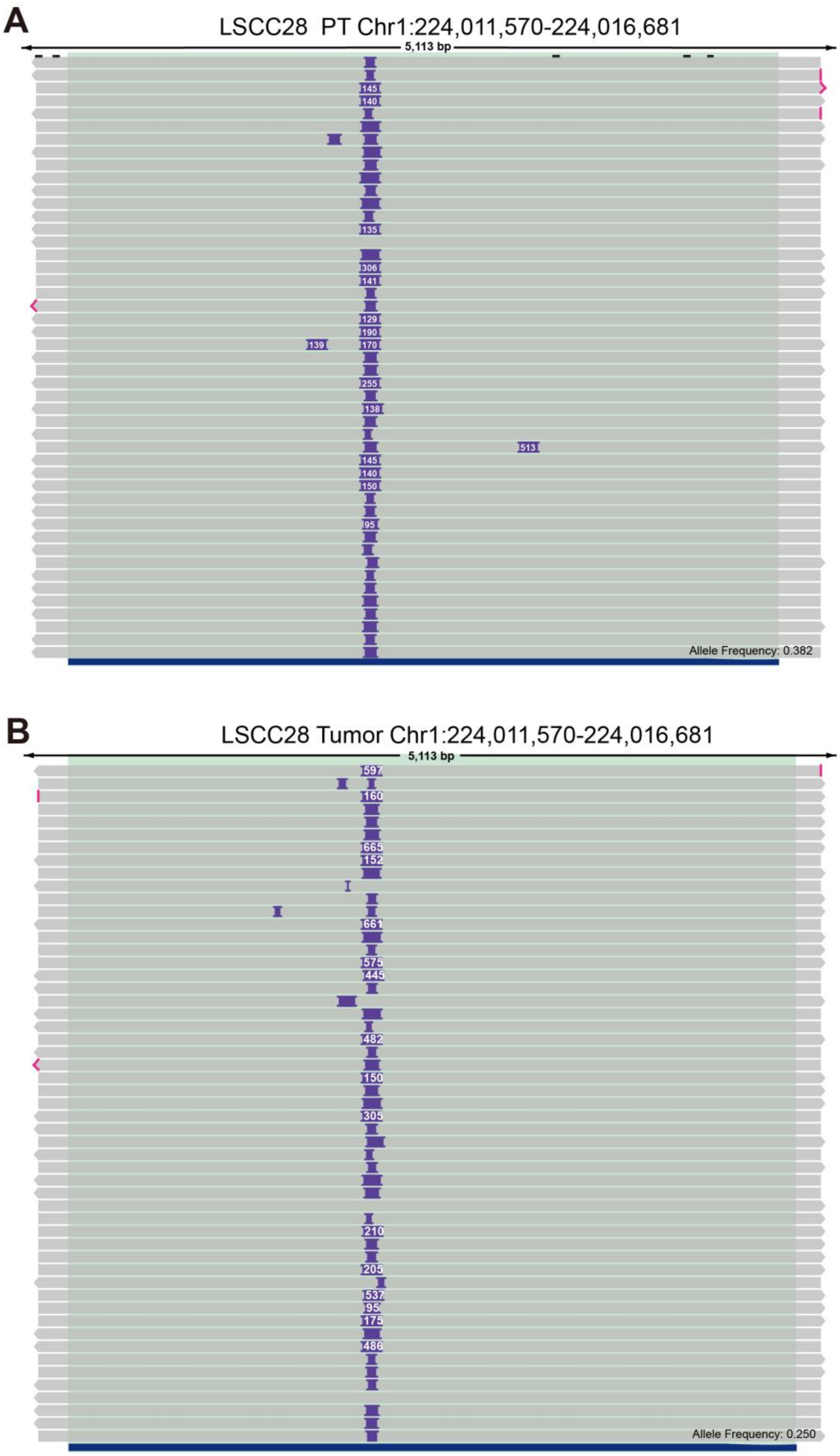


**Supplemental Figure S15. IGV plots of Samples LSCC 28.**

**(A).**IGV plot of LSCC 28 peritumoral (Chr1:224,009,042-224,018,679). Dark blue indicates the Simple repeat region with the AATGG motif. **(B).**IGV plot of LSCC 28 tumor (Chr1:224,009,042-224,018,679). Dark blue indicates the Simple repeat region with the AATGG motif. White numbers in the plot mark insertions greater than 70 bp.

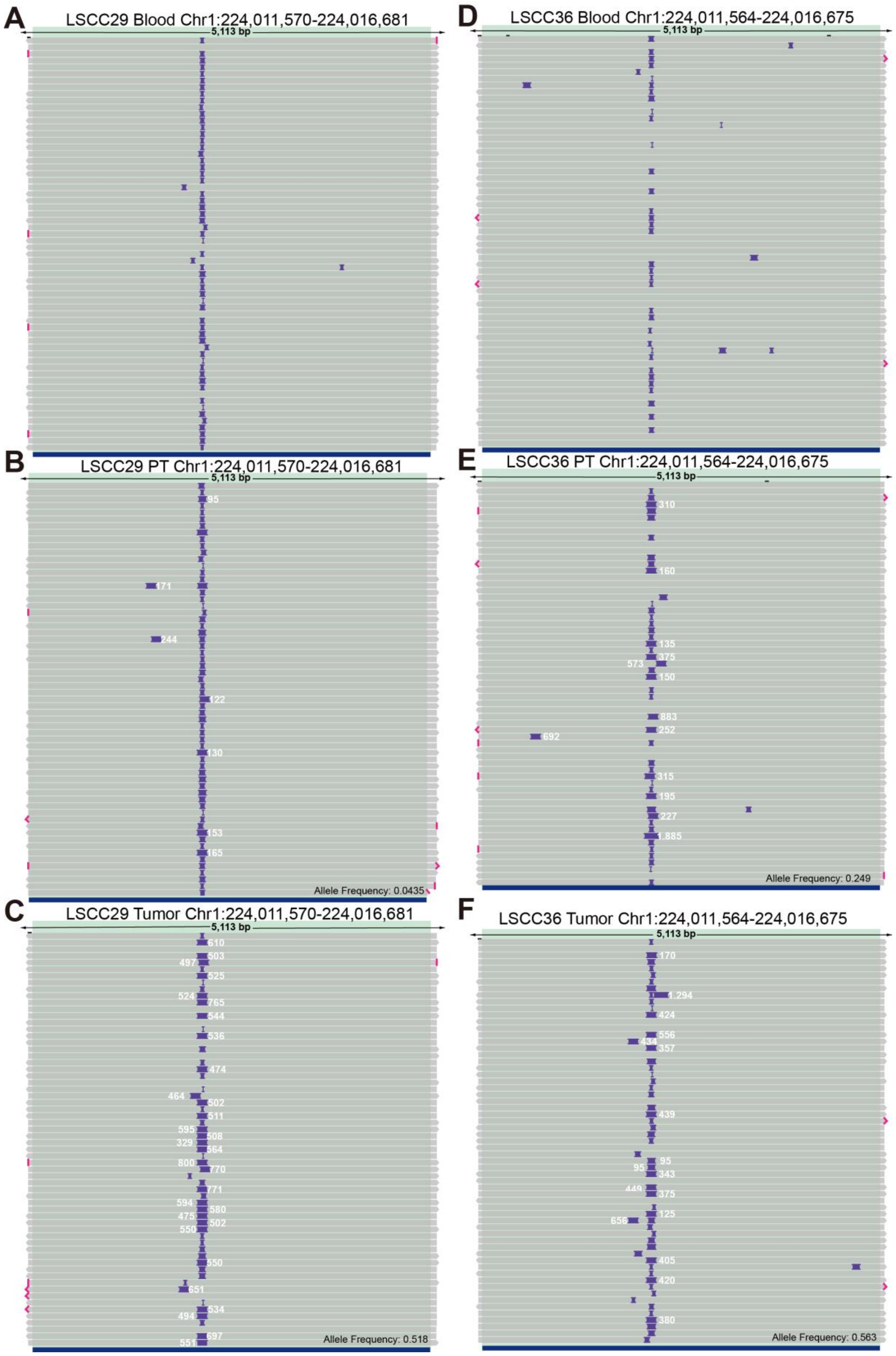


**Supplemental Figure S16. IGV plots of Samples LSCC 29 and 36.**

**(A).**IGV plot of LSCC 29 blood (Chr1:224,009,042-224,018,679). Dark blue indicates the Simple repeat region with the AATGG motif. **(B).**IGV plot of LSCC 29 peritumoral (Chr1:224,009,042-224,018,679). Dark blue indicates the Simple repeat region with the AATGG motif. White numbers in the plot mark insertions greater than 70 bp.**(C).**IGV plot of

LSCC 29 tumor (Chr1:224,009,042-224,018,679). Dark blue indicates the Simple repeat region with the AATGG motif. White numbers in the plot mark insertions greater than 70 bp. **(D).**IGV plot of LSCC 36 blood (Chr1:224,009,042-224,018,679). Dark blue indicates the Simple repeat region with the AATGG motif. **(E).**IGV plot of LSCC 36 peritumoral (Chr1:224,009,042-224,018,679). Dark blue indicates the Simple repeat region with the AATGG motif. White numbers in the plot mark insertions greater than 70 bp. **(F).**IGV plot of LSCC 36 tumor (Chr1:224,009,042-224,018,679). Dark blue indicates the Simple repeat region with the AATGG motif. White numbers in the plot mark insertions greater than 70 bp.

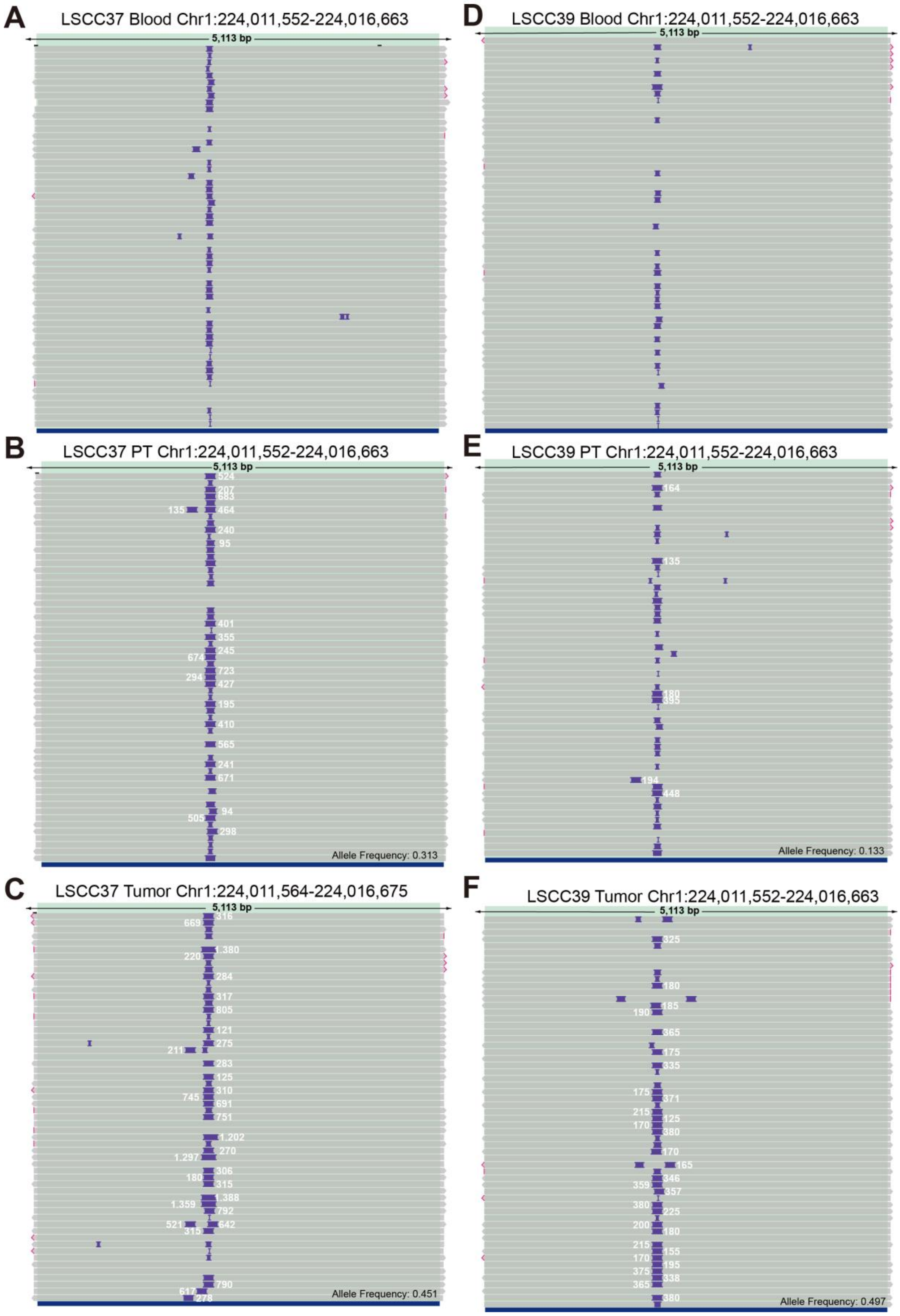


**Supplemental Figure S17. IGV plots of Samples LSCC 37 and 39.**

**(A).**IGV plot of LSCC 37 blood (Chr1:224,009,042-224,018,679). Dark blue indicates the Simple repeat region with the AATGG motif. **(B).**IGV plot of LSCC 37 peritumoral (Chr1:224,009,042-224,018,679). Dark blue indicates the Simple repeat region with the AATGG motif. White numbers in the plot mark insertions greater than 70 bp.**(C).**IGV plot of LSCC 37 tumor (Chr1:224,009,042-224,018,679). Dark blue indicates the Simple repeat

region with the AATGG motif. White numbers in the plot mark insertions greater than 70 bp. **(D).**IGV plot of LSCC 39 blood (Chr1:224,009,042-224,018,679). Dark blue indicates the Simple repeat region with the AATGG motif. **(E).**IGV plot of LSCC 39 peritumoral (Chr1:224,009,042-224,018,679). Dark blue indicates the Simple repeat region with the AATGG motif. White numbers in the plot mark insertions greater than 70 bp. **(F).**IGV plot of LSCC 39 tumor (Chr1:224,009,042-224,018,679). Dark blue indicates the Simple repeat region with the AATGG motif. White numbers in the plot mark insertions greater than 70 bp.

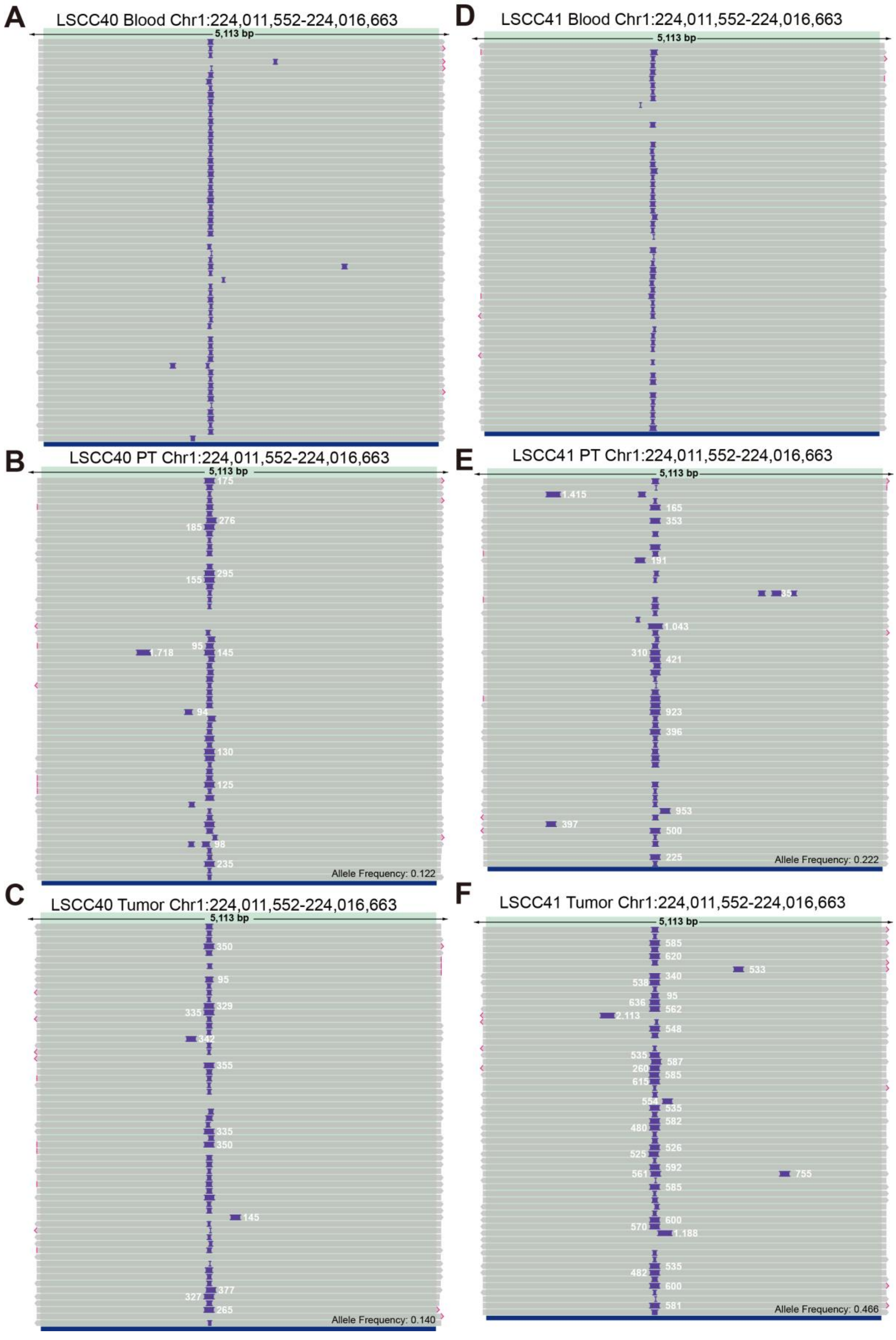


**Supplemental Figure S18. IGV plots of Samples LSCC 40 and 41.**

**(A).**IGV plot of LSCC 40 blood (Chr1:224,009,042-224,018,679). Dark blue indicates the Simple repeat region with the AATGG motif. **(B).**IGV plot of LSCC 40 peritumoral (Chr1:224,009,042-224,018,679). Dark blue indicates the Simple repeat region with the AATGG motif. White numbers in the plot mark insertions greater than 70 bp.**(C).**IGV plot of

LSCC 40 tumor (Chr1:224,009,042-224,018,679). Dark blue indicates the Simple repeat region with the AATGG motif. White numbers in the plot mark insertions greater than 70 bp. **(D).**IGV plot of LSCC 41 blood (Chr1:224,009,042-224,018,679). Dark blue indicates the Simple repeat region with the AATGG motif. **(E).**IGV plot of LSCC 41 peritumoral (Chr1:224,009,042-224,018,679). Dark blue indicates the Simple repeat region with the AATGG motif. White numbers in the plot mark insertions greater than 70 bp. **(F).**IGV plot of LSCC 41 tumor (Chr1:224,009,042-224,018,679). Dark blue indicates the Simple repeat region with the AATGG motif. White numbers in the plot mark insertions greater than 70 bp.

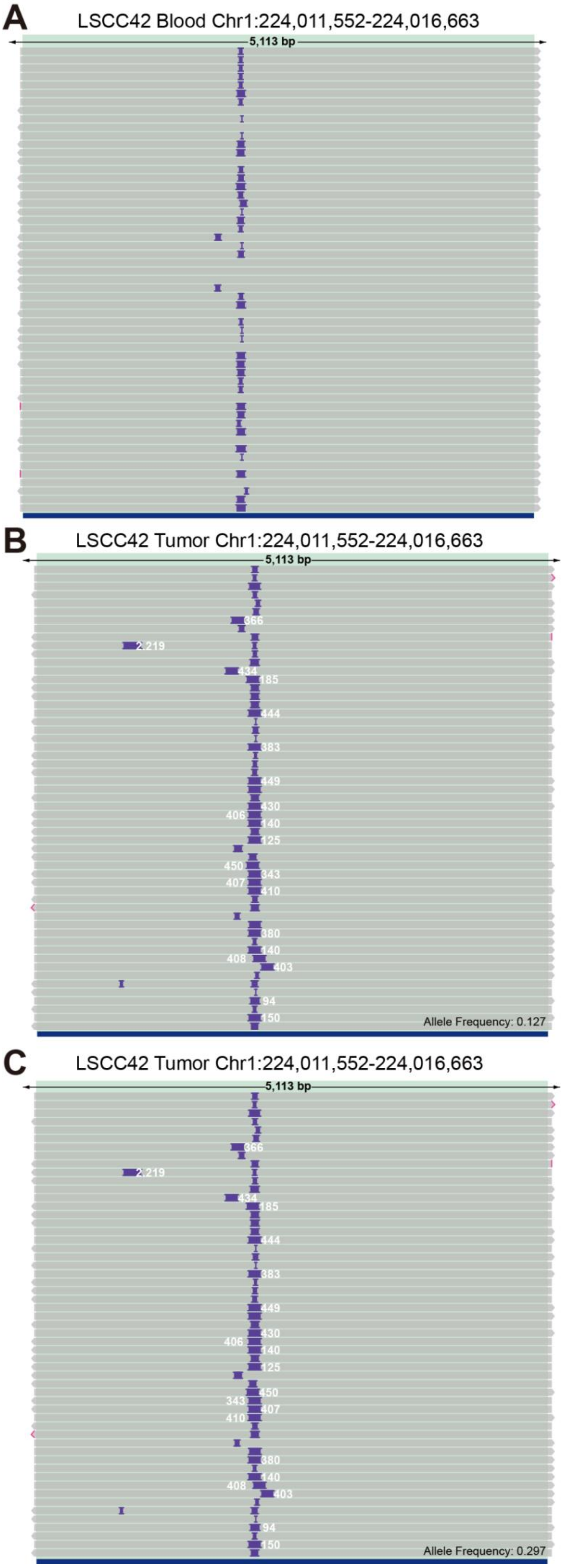
A
LSCC42 Blood Chr1:224,011,552-224,016,663
5,113 bp
B
LSCC42 Tumor Chr1:224,011,552-224,016,663
5,113 bp
Allele Frequency: 0.127
C
LSCC42 Tumor Chr1:224,011,552-224,016,663
5,113 bp
Allele Frequency: 0.297

**Supplemental Figure S19. IGV plots of Samples LSCC 42.**

**(A).**IGV plot of LSCC 42 blood (Chr1:224,009,042-224,018,679). Dark blue indicates the Simple repeat region with the AATGG motif. **(B).**IGV plot of LSCC 42 peritumoral (Chr1:224,009,042-224,018,679). Dark blue indicates the Simple repeat region with the AATGG motif. White numbers in the plot mark insertions greater than 70 bp.**(C).**IGV plot of LSCC 42 tumor (Chr1:224,009,042-224,018,679). Dark blue indicates the Simple repeat region with the AATGG motif. White numbers in the plot mark insertions greater than 70 bp.

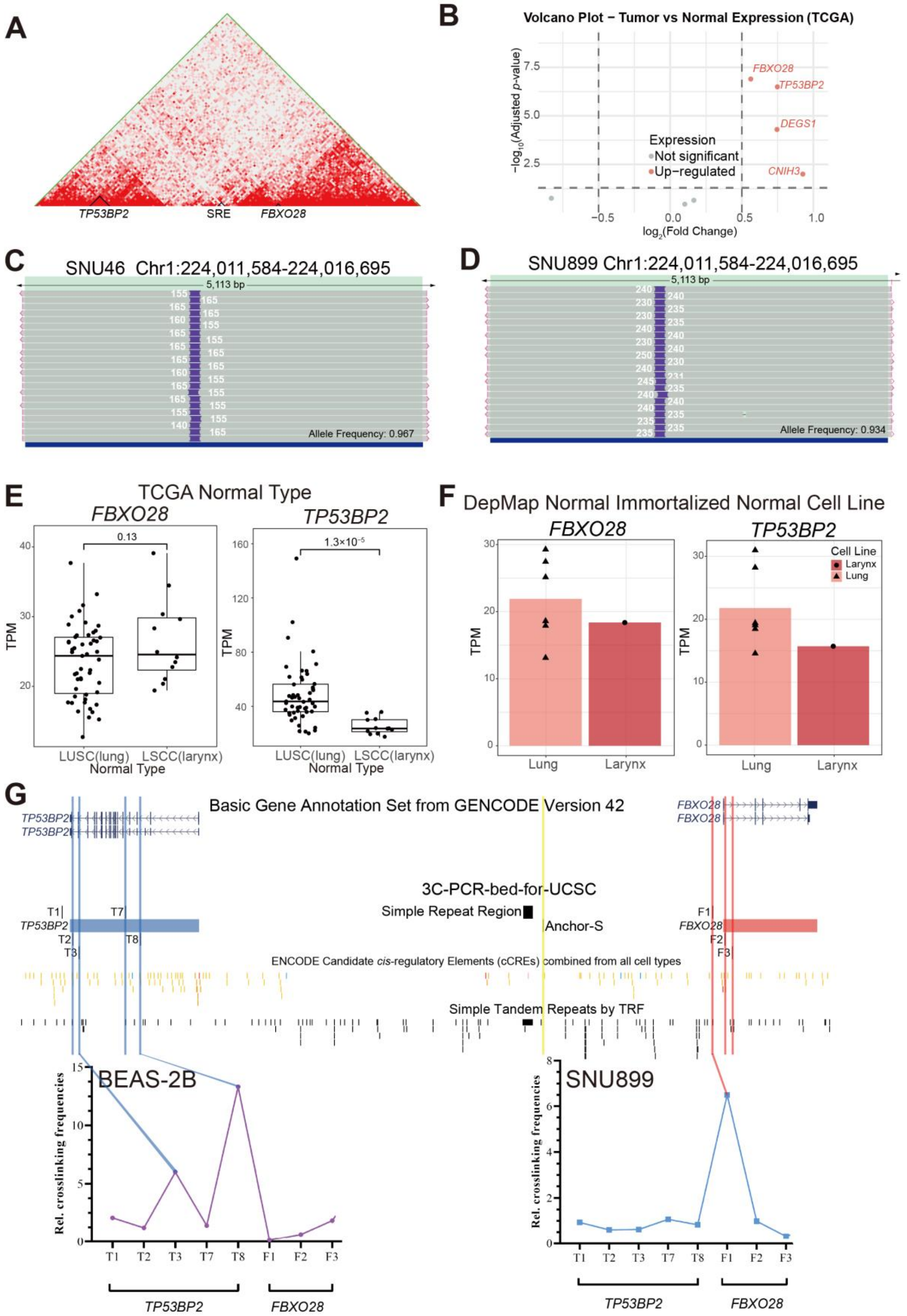


**Supplemental Figure S20. Functional investigation of SRE in cell lines.**

**(A).**Hi-C interaction matrices around the LSCC-enriched SRE and *TP53BP2*, *FBXO28* (50 kb resolution). **(B).**Volcano plot of genes near the SRE based on TCGA paired LSCC RNA-seq data. Differential expression analysis was performed on seven genes located within a 0.5 Mb window flanking the SRE. FBXO28 and TP53BP2, the two genes closest to the SRE, exhibited the most significant upregulation in tumor tissues compared to matched normal tissues (*t*-test, *q*-value < 0.05). **(C).**IGV plot of cell line SNU46

(Chr1:224,009,042-224,018,679). Dark blue indicates the Simple repeat region with the AATGG motif. White numbers in the plot mark insertions greater than 70 bp. **(D).**IGV plot of cell line SNU899 (Chr1:224,009,042-224,018,679). Dark blue indicates the Simple repeat region with the AATGG motif. White numbers in the plot mark insertions greater than 70 bp. **(E).**Box plot of *FBXO28* and *TP53BP2* expression levels in normal LSCC and LUSC samples from the TCGA database. The X-axis represents different normal samples from distinct tissue sites, where LSCC indicates normal laryngeal sample expression and LUSC indicates normal lung sample expression. **(F).** Bar plot of *FBXO28* and *TP53BP2* expression levels in immortalized laryngeal and lung epithelial cell lines. **(G).**3C-PCR results. Top: schematic genome browser view showing (from top to bottom) gene transcripts, 3C-PCR anchor-S position (4644 bp downstream of the simple repeat region), ENCODE Candidate *cis*-regulatory Elements (cCREs) combined from all cell types, and simple tandem repeats by TRF. Bottom: 3C-PCR results annotated with relative distances between Anchor-S and T1–T8 as well as F1–F2.

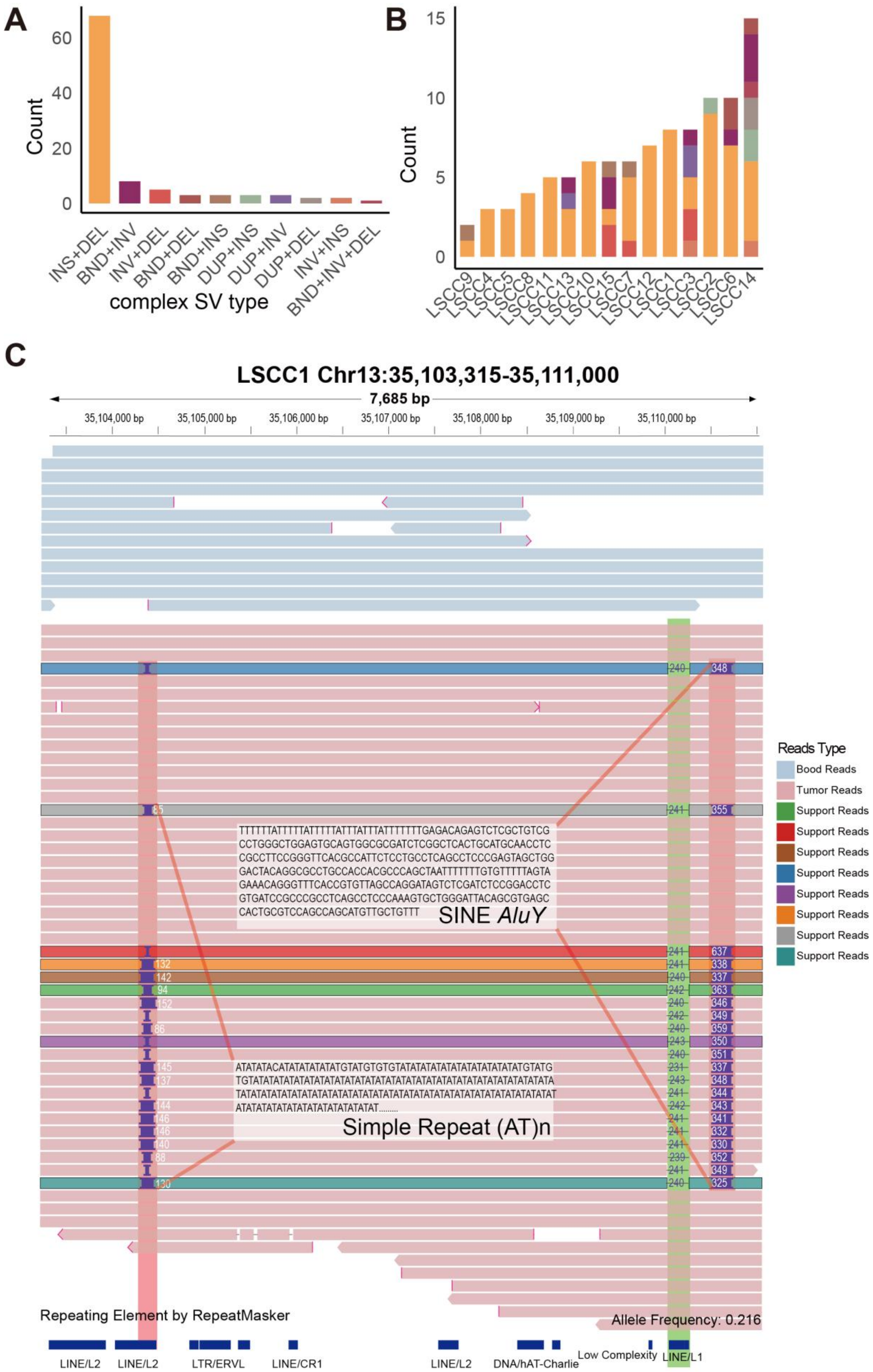

A
Count
complex SV type
INS+DEL
BND+INV
INV+DEL
BND+DEL
BND+INS
DUP+INS
DUP+INV
DUP+DEL
INV+INS
BND+INV+DEL
B
LSCC9
LSCC4
LSCC5
LSCC8
LSCC11
LSCC13
LSCC10
LSCC15
LSCC7
LSCC12
LSCC1
LSCC3
LSCC2
LSCC6
LSCC14
C
LSCC1 Chr13:35,103,315-35,111,000
7,685 bp
35,104,000 bp
35,105,000 bp
35,106,000 bp
35,107,000 bp
35,108,000 bp
35,109,000 bp
35,110,000 bp
TTTTTTATTTTTATTTTTATTTATTTATTTTTTTGAGACAGAGTCTCGCTGTCG
CCTGGGCTGGAGTGCAGTGGCGCGATCTCGGCTCACTGCATGCAACCTC
CGCCTTCCGGGTTCACGCCATTCTCCTGCCTCAGCCTCCCGAGTAGCTGG
GACTACAGGCGCCTGCCACCACGCCCAGCTAATTTTTTTGTGTTTTTAGTA
GAAACAGGGTTTCACCGTGTTAGCCAGGATAGTCTCGATCTCCGGACCTC
GTGATCCGCCCGCCTCAGCCTCCCAAAGTGCTGGGATTACAGCGTGAGC
CACTGCGTCCAGCCAGCATGTTGCTGTTT
SINE AluY
ATATATACATATATATATATGTATGTGTGTATATATATATATATATATATATGTATG
TGTATATATATATATATATATATATATATATATATATATATATATATATATATATATA
TATATATATATATATATATATATATATATATATATATATATATATATATATATATATAT
ATATATATATATATATATATATATATAT.........
Simple Repeat (AT)n
Reads Type
Bood Reads
Tumor Reads
Support Reads
Repeating Element by RepeatMasker
Allele Frequency: 0.216
LINE/L2
LTR/ERVL
LINE/CR1
DNA/hAT-Charlie
Low Complexity
LINE/L1

**Supplemental Figure S21. Summary statistics of complex SVs.**

**(A).**Bar plot of SV type co-occurrence patterns on individual reads. X-axis shows all possible co-occurring SV type pairs. **(B).**SV co-occurrence patterns across samples. X-axis represents different samples; colors indicate different SV co-occurrence types. **(C).**IGV plot of INS+DEL in LSCC1 (Chr13:35,103,315–35,111,000). Insertion and deletion sequence types are marked; reads supporting INS+DEL are color-coded.

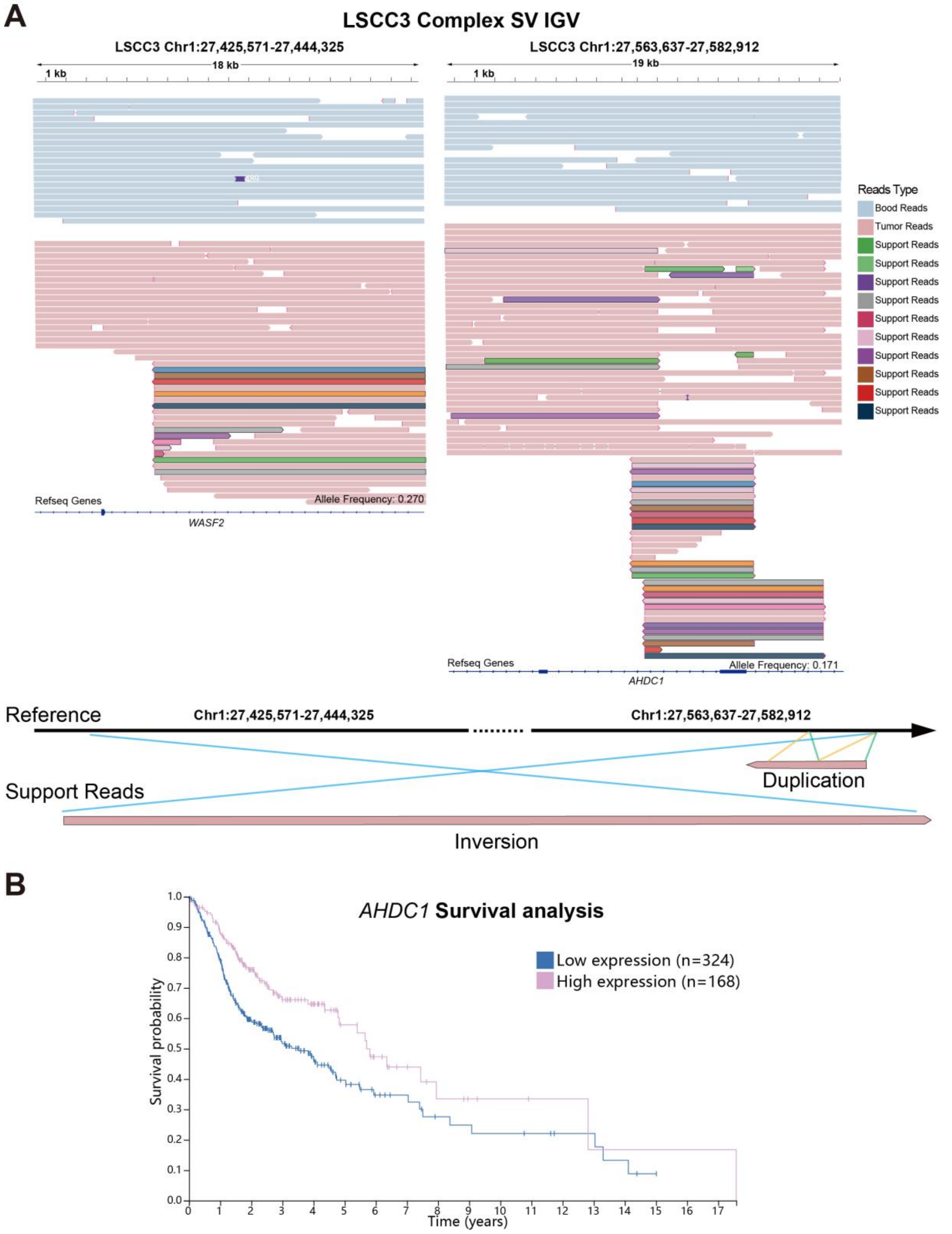


**Supplemental Figure S22. Complex SV (INV+DUP) in LSCC3.**

**(A).**IGV plot of LSCC3 INV+DUP (Chr1:27,425,571–27,582,912). Color-coded reads supporting INV+DUP and the gene AHDC1 at the INV breakpoint are shown. Schematic of read alignments for INV+DUP is shown below.**(B)**.Kaplan-Meier survival curve for the AHDC1 gene. Blue represents low expression of the AHDC1 gene, and pink represents high expression.

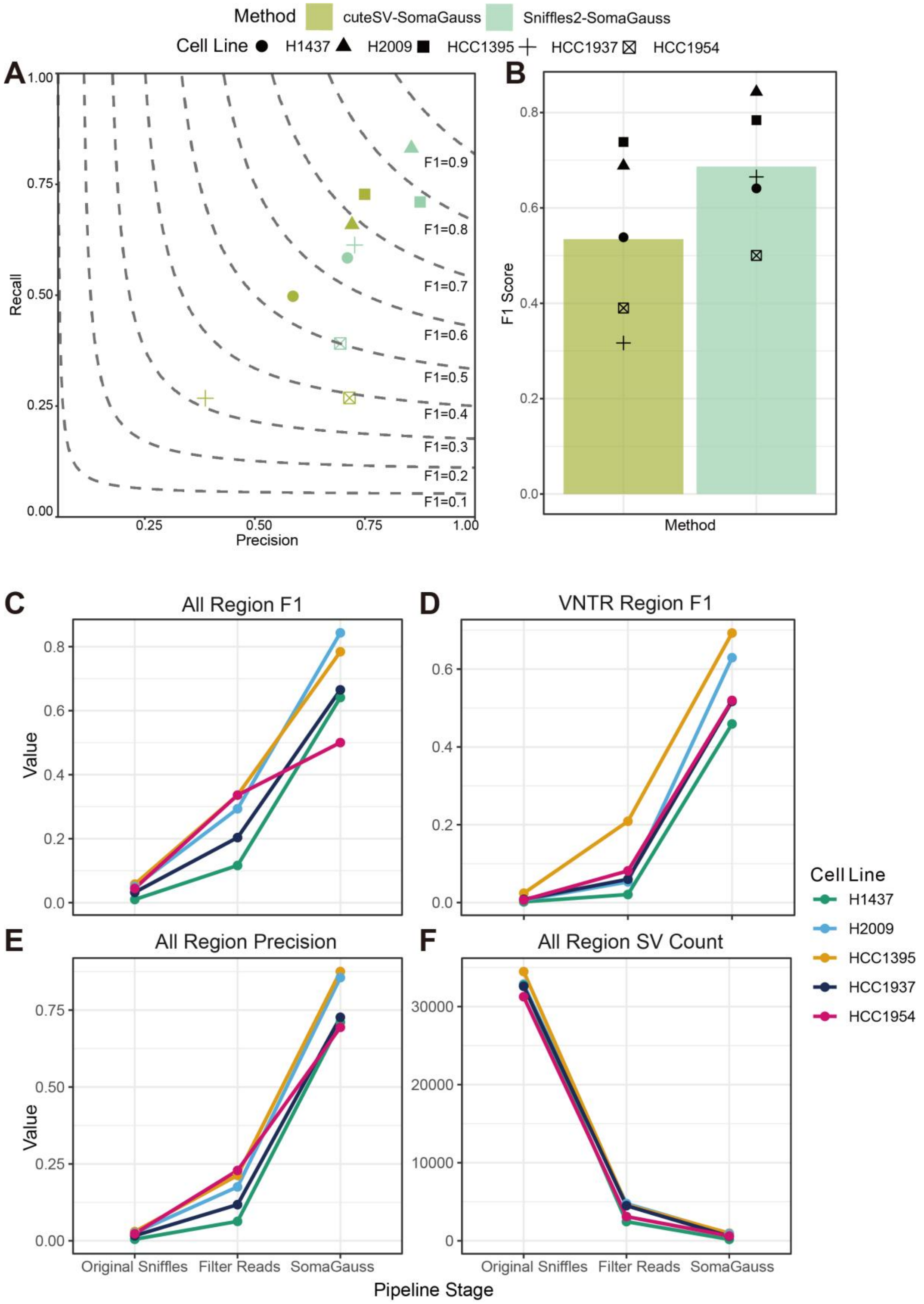


**Supplemental Figure S23. Benchmarking and staged evaluation of somatic SV detection workflows.**

**(A–B).** Performance comparison of candidate foundational callers. **(A)** Contour plot and **(B)** bar plot showing that Sniffles2 outperforms CuteSV across all tested cell lines, achieving a mean F1 score of 68.7%—a 15.3-percentage-point improvement. **(C–F).** Staged evaluation of the SomaGauss-SV pipeline. **(C)** Line plot of F1 scores across all regions, **(D)** line plot of F1 scores in VNTR regions, **(E)** line plot of precision scores across all regions, and **(F)** line plot

of SV counts across all regions. Three workflows were compared: Sniffles2 alone (Original Sniffles), Sniffles2 followed by germline read filtering (Filter Reads), and the full SomaGauss-SV pipeline (SomaGauss). The GMM-based filtering step in SomaGauss-SV substantially improved precision (from 14.5% to 71.51%) and F1 scores (overall +39.63%; VNTR regions +47.86%), demonstrating its critical role in eliminating false positives while preserving true variants.
